# A benchmark driven guide to binding site comparison: An exhaustive evaluation using tailor-made data sets (ProSPECCTs)

**DOI:** 10.1371/journal.pcbi.1006483

**Published:** 2018-11-08

**Authors:** Christiane Ehrt, Tobias Brinkjost, Oliver Koch

**Affiliations:** 1 Faculty of Chemistry and Chemical Biology, TU Dortmund University, Dortmund, Germany; 2 Department of Computer Science, TU Dortmund University, Dortmund, Germany; La Jolla Institute for Allergy and Immunology, UNITED STATES

## Abstract

The automated comparison of protein-ligand binding sites provides useful insights into yet unexplored site similarities. Various stages of computational and chemical biology research can benefit from this knowledge. The search for putative off-targets and the establishment of polypharmacological effects by comparing binding sites led to promising results for numerous projects. Although many cavity comparison methods are available, a comprehensive analysis to guide the choice of a tool for a specific application is wanting. Moreover, the broad variety of binding site modeling approaches, comparison algorithms, and scoring metrics impedes this choice. Herein, we aim to elucidate strengths and weaknesses of binding site comparison methodologies. A detailed benchmark study is the only possibility to rationalize the selection of appropriate tools for different scenarios. Specific evaluation data sets were developed to shed light on multiple aspects of binding site comparison. An assembly of all applied benchmark sets (ProSPECCTs–Protein Site Pairs for the Evaluation of Cavity Comparison Tools) is made available for the evaluation and optimization of further and still emerging methods. The results indicate the importance of such analyses to facilitate the choice of a methodology that complies with the requirements of a specific scientific challenge.

## Author summary

Binding site similarities are useful in the context of promiscuity prediction, drug repurposing, the analysis of protein-ligand and protein-protein complexes, function prediction, and further fields of general interest in chemical biology and biochemistry. Many years of research have led to the development of a multitude of methods for binding site analysis and comparison. On the one hand, their availability supports research. On the other hand, the huge number of methods hampers the efficient selection of a specific tool. Our research is dedicated to the analysis of different cavity comparison tools. We use several binding site data sets to establish guidelines which can be applied to ensure a successful application of comparison methods by circumventing potential pitfalls.

## Introduction

In parallel with the ever increasing number of available protein structures in the Protein Data Bank (PDB)[1], various *in silico* techniques were developed to apply this structural knowledge[2]. In addition to molecular docking, structure-based pharmacophore searches, and MD (molecular dynamics) simulations, the comparison of ligand binding sites of available protein structures became a promising tool to exploit accessible knowledge and apply it to a range of scientific problems. It has proven to be beneficial in numerous drug discovery projects[3–5] and has been successfully applied in protein function prediction[4] and polypharmacology prediction[6]. However, the number of available binding site comparison methods is still growing[6] and it is becoming increasingly difficult to choose the most suitable method for a specific area of research. Although binding site similarities can now be retrieved from elaborate similarity databases[7–10], it is often advisable to perform additional comparisons, or it may even be necessary if proprietary structures are used. The importance of selecting an appropriate program and underlying binding site similarity metric is comprehensively summarized in an article by Kellenberger *et al*.[11]. The impact of the similarity measure on the study outcome was recently analyzed for one cavity comparison method[12]. The authors conclude that an assessment of the similarity measures employed is essential for the evaluation and optimization of novel comparison approaches.

Binding site comparisons can be applied to investigate minor dissimilarities between evolutionarily related binding sites, as well as to reveal similarities between proteins that share no obvious global (sequence or structural) similarity. The latter kind of similarity between unrelated proteins is not only important for the analysis of off-target effects or in investigating polypharmacological activity of small molecules, but can also contribute at the very beginning of a drug discovery process by suggesting potential novel scaffolds and compound classes[13]. It is also possible to predict the function of uncharacterized proteins using structural data from well characterized proteins with a similar binding site. A detailed review of successful applications of various binding site comparison methods can be found elsewhere[5].

Usually, published binding site comparison algorithms have been benchmarked using specific data sets, which are highly correlated with distinct application domains. However, standardized benchmark data sets, as known for other *in silico* methodologies[14–16], have never been developed for cavity comparison tools. This often precludes the selection of a suitable tool. The analysis presented herein aims to enable interested scientists to choose an appropriate tool for comparing ligand binding sites for specific applications. We have designed various independent and objective data sets to elucidate strengths and weaknesses of different comparison tools. The impact of these data sets with regard to various applications for binding site comparison will be discussed in detail. The foremost question to be addressed when selecting a suitable tool is: “What is the aim of the binding site comparison?”

It has to be stated that binding site similarity “lies in the eye of the beholder”. As a matter of fact, a method that can be used for all conceivable application domains might never be developed. The selection of the most suitable method will always depend on the available resources, the application domain, and the individual needs of the researcher. Sometimes, a combination of different tools could lead to optimal outcomes. Our results provide a number of indicators for the weaknesses and putative application domains of selected binding site comparison approaches. Ultimately, data sets and decision criteria are provided, which enable researchers to rationalize the choice of a binding site comparison method. Such analyses will hopefully assist in the development of an appropriate workflow, which ensures meaningful results. The complete sets of similar and dissimilar protein cavity pairs (ProSPECCTs–Protein Site Pairs for the Evaluation of Cavity Comparison Tools) are available for further benchmark studies and the evaluation of alternative and novel tools.

### Brief introduction to binding site comparison methodologies

Due to the huge number of available binding site comparison algorithms[5], a large-scale analysis of all methods is beyond the scope of this article. We have therefore restricted the evaluation to a small, but still diverse subset of promising algorithms. These were derived from an analysis of successful applications within medicinal chemistry projects[5]. A comparison of web server-based tools became infeasible due to the number and size of analyzed data sets, so we restricted our evaluation to standalone tools. The methods analyzed herein and their fields of utilization are summarized in Table 1. Intriguingly, the success of nearly all of those studies resulted from the use of binding site comparison as part of a workflow combining different computational methods, e.g. MD simulations or molecular docking studies. A recent, impressive example shows that the combination of various tools of structure-based modeling and binding site comparison delivers insight into putative mechanisms of drug action[17].

**Table 1:** Summary of the methods analyzed in the present work and their respective fields of successful application.

method	application (note)	availability (URL)
residue-based		
Cavbase[20,21]	protein-ligand interactions[32], virtual screening[33], evolutionary relationships[34], drug repurposing[35]	available from the CCDC (https://www.ccdc.cam.ac.uk/)
RAPMAD[31]	(similar to Cavbase, histogram-based)	available from the CCDC (https://www.ccdc.cam.ac.uk/)
FuzCav[36]	protein-ligand interactions[37]	upon request (http://bioinfo-pharma.u-strasbg.fr/labwebsite/download.html)
PocketMatch[24]	function prediction[38], polypharmacology[39], evolutionary relationships[40]	download (http://proline.physics.iisc.ernet.in/pocketmatch/)
SiteAlign[18]	protein-ligand interactions[37,41]	upon request (http://bioinfo-pharma.u-strasbg.fr/labwebsite/download.html)
SMAP (based on SOIPPA[42])[43]	polypharmacology[44], drug repurposing[45,46]	download (http://compsci.hunter.cuny.edu/~leixie/smap/smap.html)
TM-align[27]	drug repurposing[47]	download (https://zhanglab.ccmb.med.umich.edu/TM-align/)
surface-based		
ProBiS[48]	function prediction[49], off-target prediction[50]	download (http://insilab.org/probis-algorithm/)
VolSite/Shaper[23]	protein-ligand interactions[37]	upon request (http://bioinfo-pharma.u-strasbg.fr/labwebsite/download.html)
SiteEngine[51]	protein-protein interactions[52]	download (http://bioinfo3d.cs.tau.ac.il/SiteEngine/)
SiteHopper[25]	evolutionary relationships[53]	OpenEye, available to academic users (https://www.eyesopen.com/sitehopper/)
interaction-based		
IsoMIF (based on IsoCleftFinder[54])[22]	drug repurposing[55], off-target prediction[17]	download (http://biophys.umontreal.ca/nrg/NRG/IsoMIF.html)
KRIPO[56]	off-target prediction[26,57,58], polypharmacology[26]	download (https://github.com/3D-e-Chem/kripodb, https://github.com/3D-e-Chem/kripo), KNIME nodes (https://www.knime.com/3d-e-chem-nodes-for-knime)
TIFP[19]	virtual screening[59]	upon request (http://bioinfo-pharma.u-strasbg.fr/labwebsite/download.html)
Grim[19]	(similar to TIFP, graph-based)	upon request (http://bioinfo-pharma.u-strasbg.fr/labwebsite/download.html)

Herein, we analyzed binding site comparison methods which are based on fingerprints (e.g., SiteAlign[18] and TIFP (Interaction Fingerprint Triplets)[19]), graphs (e.g., Cavbase[20,21] and IsoMIF[22]), grids (VolSite/Shaper[23]), and those that make use of alternative approaches (PocketMatch[24] and SiteHopper[25]). KRIPO (Key Representation of Interaction in Pockets), that was originally designed for sub-pocket matching to facilitate bioisosteric replacement, can also be applied for ligand binding site comparison[26] and was included in our analysis. Additionally, TM-align[27], which was developed to compare protein structures based on their overall structure, was evaluated as it was successfully applied in various medicinal chemistry scenarios. The freely available tools CMASA[28], COFACTOR[29], and PocketFEATURE[30] could not be analyzed. Both CMASA and COFACTOR can only be applied with pre-prepared data sets; they enable the user to compare the binding site of interest against sets of precomputed binding sites. PocketFEATURE is not publicly available.

Grim (Graph Interaction Matching)[19] and RAPMAD (Rapid Pocket Matching using Distances)[31] were included as they make use of binding site representations highly similar to TIFP and Cavbase. The impact of the underlying data structures was evaluated (fingerprints vs. graph models for TIFP and Grim, histograms vs. graph models for RAPMAD and Cavbase, respectively). While fingerprint- and histogram-based methods are usually characterized by low computational demands, graph models enable a more accurate binding site characterization accompanied by higher run times.

**Fig 1:**
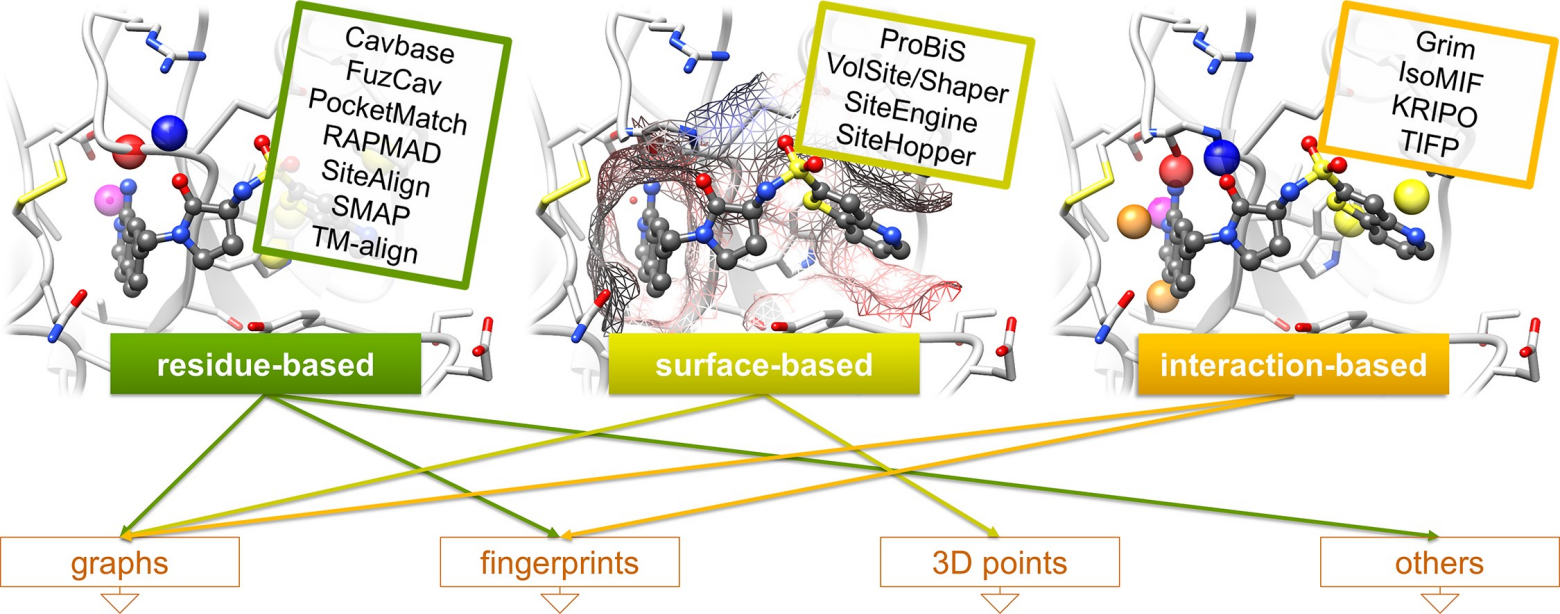
Binding site modeling approaches for different comparison algorithms. The binding site of coagulation factor Xa (PDB ID 1f0r, chain A) bound to the nanomolar inhibitor RPR208815 is shown together with a schematic representation of the ways in which binding site features are modeled. The methodologies are connected with the corresponding underlying data structures used for the comparison. Binding site visualizations were generated using UCSF Chimera[60].

The representation of the binding site for the subsequent comparison algorithm has a large impact on the outcome of the investigations. We therefore classified all approaches as depicted in Fig 1. While most tools encode the binding site features based on the underlying ligand-interacting residues, other approaches use surface-based binding site representations, e.g. through a projection of physicochemical site features onto the respective surface patches. Probably the highest level of abstraction is achieved by programs that encode protein-ligand interactions, i.e. they do not depend on a distinct set of residue classes or functional groups, but on different types of interactions. While the residue type is crucial for most residue- and surface-based methods, interaction-based methods often rely on the nature of the bound ligand.

However, this classification only holds true for binding site modeling. The final scoring scheme applied to a binding site pair match might also include other properties, for example, surface similarity. Moreover, the data structure for comparison differs for methods within a category. While residue-based comparisons are achieved using versatile approaches, trends can be derived for surface- and interaction-based methods. Interaction-based methods make use of graph and fingerprint representations. Graph and 3D point approaches are often used for surface-based comparisons.

Brief descriptions of the methods and explanations of the scoring schemes are given in the SI (S1 Text) to outline the general concept underlying each method. This information on different scoring measures and comparison approaches is included to support the understanding of the outcomes presented herein. The explanations are by no means complete, but are focused on the most essential points. Although these descriptions are sufficient for the scope of this study, we encourage readers to refer to the methods’ publications to gain further insight into usage and parameter details. In most cases, default settings were applied in our work, hence parameter optimization might lead to improved performance.

## Results

### Benchmark data sets

As for many other computational methods, the success of binding site comparison methods has to be evaluated using benchmark data sets. Some evaluation sets previously created to test the tools analyzed herein are summarized in the SI (S1 Table). Although we do not claim that our collection is comprehensive, there are no commonly used, state-of-the-art evaluation data sets available. Standardized benchmark sets are accessible for broadly applied modeling approaches such as pharmacophore searches[61] and molecular docking[16,62]. In contrast, the high diversity of the applied benchmark sets for binding site comparison makes it difficult to draw definitive conclusions in comparing the different tools.

There is a need for a common evaluation scheme to assess the applicability of highly diverse methodologies. Even knowledge of the underlying concept is not sufficient to make a confident choice of an appropriate tool. For example, Cavbase and SiteAlign make use of similar binding site representations, but the underlying comparison algorithms and scoring schemes differ. It is not obvious whether a certain method should be used preferentially. The evaluation of SiteAlign (S1 Table) would suggest that the method is able to detect evolutionary relationships by identifying similarities between binding sites with similar biological functions. However, it has been successfully applied for the investigation of protein-ligand interactions (Table 1). TM-align was never evaluated as a cavity comparison tool, but it has given hints on interesting binding site relationships (Table 1).

The high number of application domains encouraged us to develop novel, objective data sets with respect to specific aspects of binding site comparison. Pairs of similar and dissimilar binding sites enable an objective and detailed analysis of available tools. Various benchmark studies were performed for the methods mentioned above. Based on the results, we attempted to discern whether a suitable method, both in general and for specific application domains, can be selected.

The first data set (structures with identical sequences) was designed to evaluate the tools’ sensitivity to the binding site definition. This definition often depends on the size and location of bound ligands. Different ligands can address various regions of the binding site of interest (sub-pockets). Because of these different site definitions, similar sub-pockets are more difficult to match. Although binding sites can interact with a broad variety of ligands, they share common properties and distinct similarities. Thus, the scoring scheme has to be optimized for this scenario to enrich similar binding site pairs with different cavity definitions in a ranked list. Data set 1 contains structures with identical sequences, which bind to chemically different ligands located at identical sites, leading to diverse binding site definitions used for the comparisons.

The second data set assesses the tools’ performance with respect to the binding site flexibility. This is an important factor when comparing two similar pockets. The data set is based on protein models extracted from ligand-bound solution NMR structures with more than one model in the structure ensemble.

The next two data sets were used to elucidate the scoring metrics’ discrimination performance on nearly identical binding sites differing by a single substitution or by multiple mutations. Artificial protein structures were created by randomly picking binding site residues and substituting them with physicochemically different residues (decoy set 1) and residues that lead to a change in the binding sites’ size and physicochemical properties (decoy set 2). A comparison of binding site pairs of proteins on a data set containing both the original sequences and their artificially generated counterparts should lead to an enrichment of binding site pairs of proteins with the original sequences. Pairs of original and modified binding sites should obtain lower similarity scores.

Two pre-existing data sets were used to evaluate whether tools are able to differentiate between binding sites that bind different ligands and to identify similarities between binding sites occupied by identical or highly similar ligands. One such data set was described by Kahraman *et al*.[63]. This was originally designed to evaluate whether binding site shape and ligand shape are related. The authors state that the variability of binding site shape cannot solely be explained by the conformational variability of the ligand. Although the data set structures are derived from unrelated proteins, it was not investigated whether there are local similarities between the binding sites with similar ligands. In contrast, the data set of Barelier *et al*.[64] comprises 62 pairs of unrelated proteins binding to similar ligands. It includes 19 pairs of binding sites that show local similarities (as “observed” by the ligand atoms) whereas the remaining pairs do not display any obvious resemblance.

Finally, we established a data set of binding site pairs whose similarity was correctly identified by at least one binding site comparison tool as described in a recent perspective[5]. We combined those similar binding site pairs with a diverse data set of sc-PDB[65] derived binding sites. A comparison of the query structures (binding sites with known similarities) to the complete data set (data set of successful applications) can be performed. This analysis allows the assessment of whether the tools are able to enrich similar site pairs within the high-scoring hits. This benchmark set contains the most interesting pairs as their similarity proved useful in various medicinal chemistry projects.

Table 2 gives an overview of the benchmark data sets, their main goals, composition, and experimental quality (if applicable). For some data sets, we generated two versions (e.g. data set 1 and data set 1.2) to focus on specific aspects of binding site comparison. Details regarding the individual data sets can be found in the Methods section and S1 Fig and S2, S3 and S5–S11 Tables.

**Table 2:** Summary of benchmark data sets used to analyze the individual strengths and weaknesses of different binding site comparison tools. Similar and dissimilar binding site pairs are referred to as active and inactive pairs, respectively. The average overall G-factors were calculated with PROCHECK[66] and PROCHECK-NMR[67] to enable the quality comparison between all data sets including data set 2. Mean, standard deviation (stddev), minimum and maximum are given for all quality critieria.

goal	number of comparisons (similar or active / dissimilar or inactive pairs)	resolution (mean ± stddev, minimum, maximum) [Å]	R_work_ (mean ± stddev, minimum, maximum)	average overall G-factor (mean ± stddev, minimum, maximum)
structures with identical sequences (data set 1)				
sensitivity with respect to the binding site definition, score range for active and inactive pairs	13,430 / 92,846 (12 groups of structures with identical sequences)	1.79 ± 0.37, 0.8, 2.71	0.174 ± 0.027, 0.091, 0.264	0.023 ± 0.23, -1.27, 0.6
structures with identical sequences and similar ligands (data set 1.2)				
impact of ligand diversity on binding site comparison	241 / 1,784	1.73 ± 0.37, 0.92, 2.5	0.171 ± 0.025, 0.104, 0.232	0.019 ± 0.22, -0.57, 0.6
NMR structures (data set 2)				
sensitivity with respect to the binding site flexibility	7,729 / 100,512 (17 structural ensembles of diverse proteins)	n.d.	n.d.	-0.279 ± 0.705, -2.8, 0.21
decoy set 1 (data set 3)				
differentiation between binding sites with different physicochemical properties	13,430 / 67,150 (complete data set) 13,430 / 13,430 (data set with five residue variants)	n.d.	n.d.	n.d.
decoy set 2 (data set 4)				
differentiation between binding sites with different physicochemical and shape properties	13,430 / 67,150 (complete data set) 13,430 / 13,430 (data set with five residue variants)	n.d.	n.d.	n.d.
Kahraman data set[63] without phosphate binding sites (data set 5)				
classification of proteins binding to identical ligands and cofactors	920 / 5,480	2.02 ± 0.37, 0.88, 2.9	0.202 ± 0.033, 0.089, 0.265	0.166 ± 0.228, -0.56, 0.47
Kahraman data set[63] (data set 5.2)				
original data set	1,320 / 8,680	2.02 ± 0.4, 0.88, 2.9	0.201 ± 0.031, 0.089, 0.265	0.162 ± 0.218, -0.56, 0.47
Barelier data set[64] (data set 6) including cofactors (data set 6.2)				
identification of distant relationships between protein binding sites with identical ligands which “observe” a similar environment	19 / 43	2.16 ± 0.44, 0.93, 3.1	0.196 ± 0.027, 0.104, 0.25	0.117 ± 0.23, -1.46, 0.53
data set of successful applications (data set 7)				
recovery of known binding site similarities within a set of diverse proteins	115 / 56,284 (49 query structures)	1.98 ± 0.43, 0.8, 3.25	0.191 ± 0.029, 0.101, 0.284	0.13 ± 0.208, -2.8, 1.35

As all data sets include active (i.e. similar) and inactive (i.e. dissimilar) pairs, the ROC (receiver operating characteristics) curves as well as EFs (enrichment factors) at different percentages of the screened data set for all analyses can be calculated. A detailed summary of all AUC (area under the ROC curve) values and EFs obtained for the applied tools is given in S14, S16, S18, S20, S22, S24, S26, S28, S30 and S32 Tables. The significance of the AUC values and the differences between the methods’ AUC values for the different data sets are provided in S15, S17, S19, S21, S23, S25, S27, S29, S31 and S33 Tables.

### Benchmark studies

#### Structures with identical sequences

**Fig 2:**
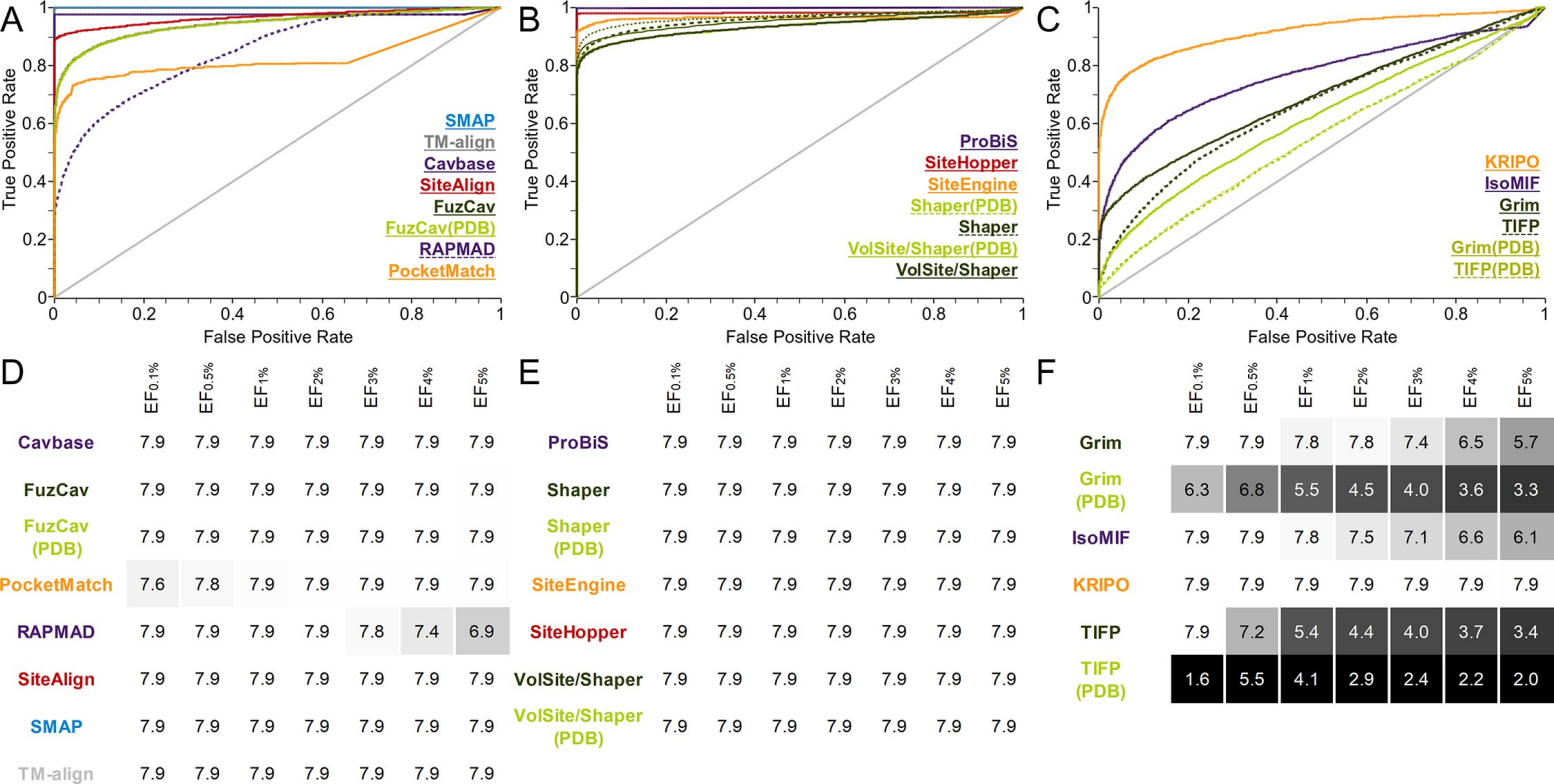
Evaluation of different binding site comparison tools with respect to the data set of structures with identical sequences. A-C) The ROC curves for residue- (A), surface- (B), and interaction-based (C) comparison methods. The name of the tool is colored according to its corresponding ROC curve. The binding site comparison tools are sorted in descending order with respect to the AUC. Thin lines represent the resulting ROC curve for the scoring scheme that yielded the highest AUC. (A) A slightly higher AUC for SiteAlign was obtained if distance d2 was applied for binding site pair ranking. (B) For the surface-based methods, the Tanimoto (color) for Shaper or VolSite/Shaper and the ColorTanimoto for SiteHopper led to the highest AUC. (C) The use of the Tanimoto coefficient as similarity measure led to the highest AUC for TIFP(PDB). D-F) EFs for residue- (D), surface- (E), and interaction-based (F) comparison methods. A linear color gradient ranging from white for the highest value to gray to black for the lowest value was applied for the EFs at different percentages of screened data set.

The data set consists of structures with identical sequences whose binding sites are occupied by different ligands. It was generated to evaluate the sensitivity of binding site comparison tools with respect to the binding site definition. A tool which is not able to enrich similar binding sites accommodating different ligands should not be applied for drug repurposing projects or the prediction of putative off-targets. We assembled 12 groups of structures with identical sequences which bind to different ligands at a similar location. Figures of the respective binding site alignments can be found in the SI (S1 Fig) together with statistics on the pairwise Tanimoto coefficients of the ligands within the groups. While the binding site flexibility plays a minor role in this data set (S2 Table), the differences in size and the chemical nature of the ligands affect the performance of the tools to a varying extent. The resulting ROC curves and EFs are depicted in Fig 2. Most differences between the AUC values are significant (S15 Table) except for ProBiS and SMAP which perform identically. There is also no significant difference between the performance of FuzCav, Shaper, and VolSite/Shaper for PDB or MOL2 files as input.

**Fig 3:**
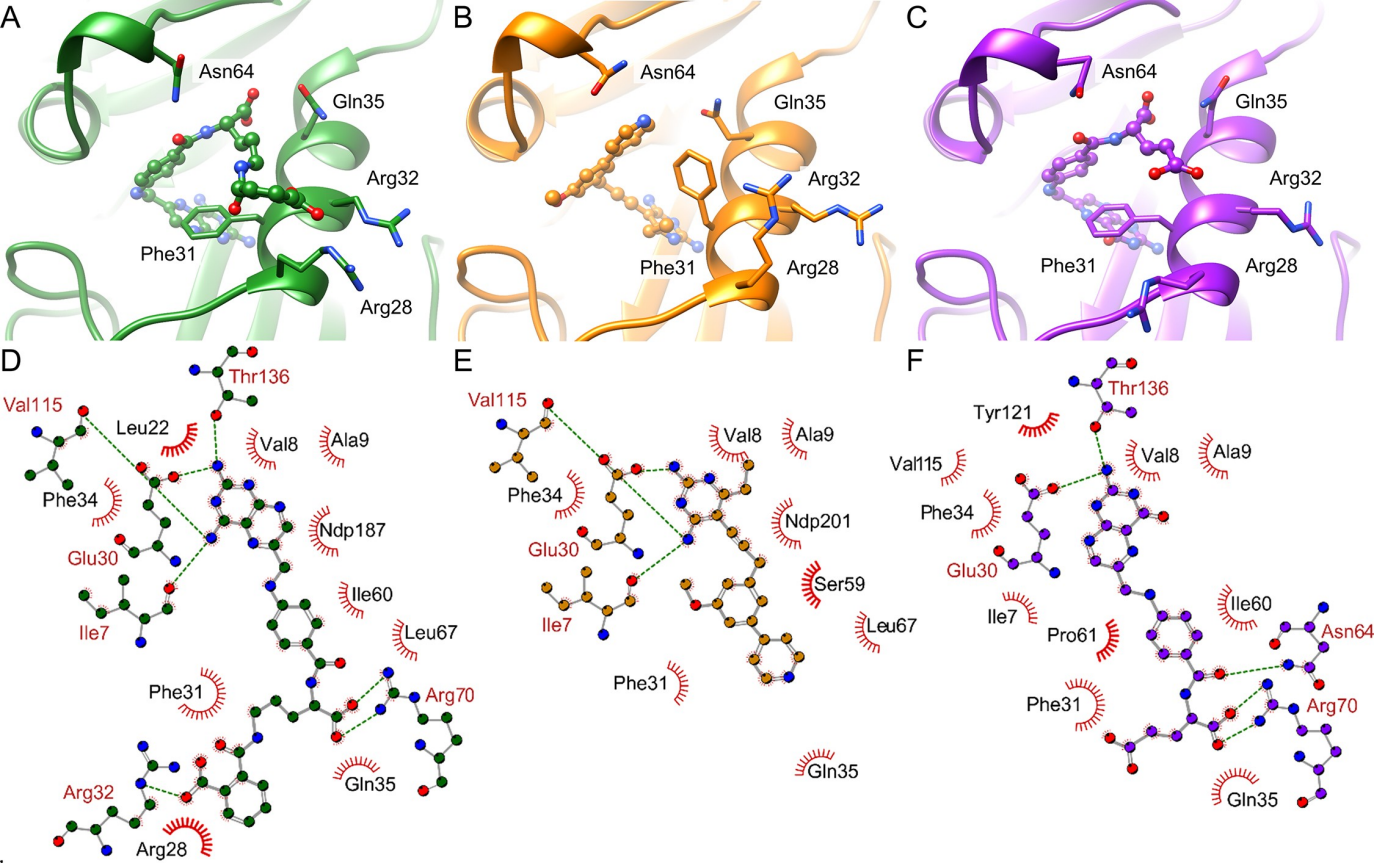
**Changes in the molecular interaction patterns of dihydrofolate reductase ligands and changes in the binding site upon ligand binding (PDB ID 1ohk, chain A (A and D); PDB ID 4kd7, chain A (B and E); PDB ID 1drf, chain A (C and F)).** A-C) Representation of the binding site structures. Figures were generated using UCSF Chimera[60]. D-F) Schematic view of the crucial interactions between protein and ligand. Figures were generated using LigPlot^+^[69].

The plots of the EFs for this data set reveal that nearly all methods are characterized by a high early enrichment, i.e. an enrichment of similar pairs at the top of the list of ranked binding site similarities. In contrast, an analysis of the AUC values, which represent the tools’ overall performance, shows that some methods are highly sensitive with respect to binding site definition (FuzCav, PocketMatch, RAPMAD). This effect is most pronounced for binding site comparison tools that compare protein-ligand interactions. The interaction patterns of similar protein binding sites with different ligands strongly deviate from each other (Fig 3). An overview of the aligned protein-ligand complexes can be found in S1 Fig. IsoMIF and KRIPO clearly outperform Grim and TIFP. While the former solely rely on the ligand for the site definition, the latter derive interactions based on the bound molecules. Both methods are highly dependent on the ligand’s size and its chemical nature. Interaction-based tools should therefore be used in combination with a large set of available protein-ligand structures. If the number of known complex structures is low, it may be useful to perform molecular docking studies for further known ligands and use the resultant binding poses for the comparison in addition to experimentally derived complex structures. Moreover, the use of “compound ligands” might improve the results, i.e. using a number of ligands from different aligned structures for binding site definition. A similar approach was successfully applied in a study of binding site similarities[68], but was restricted to similar or identical ligands. Alternatively, the analysis of ligand substructures (fragments) and their corresponding interaction patterns (sub-pockets), as described for KRIPO[26,56], might increase the robustness of binding site similarity searches.

Interaction-based approaches perform convincingly and similarly to residue- and surface-based methods if applied to a reduced data set of structures with identical sequences. In this reduced set, comprising only 45 structures, the Tanimoto coefficient between all ligand pairs of one group never falls below 0.6 (S3 Table). The performance of nearly all methods improved with this data set. In particular, the interaction-based methods’ AUC values and EFs increased significantly. However, only slight improvements were observed for surface- and residue-based methods (S2 Fig), as their binding site representation does not depend on bound ligands. The ligands in this data set show highly similar interaction patterns. In practice, structures of similar binding site pairs of unrelated proteins interacting with similar or even identical ligands are rarely known[64]. Consequently, residue- and surface-based methods should be preferentially used for drug repurposing, polypharmacology studies, or off-target prediction.

A comparison of the graph-based methods Cavbase and Grim to their faster counterparts RAPMAD and TIFP shows a drop in sensitivity for the less accurate histogram- and fingerprint-based methods. Lower accuracy can be expected for nearly all fingerprint-, histogram-, or sorted-list based methods. The use of these tools is only recommended for very large data sets because the major benefit is their low run time. Nevertheless, the early enrichment is promising for all methods, underlining their general applicability. For the comparison of a small number of complex structures, e.g. to deduce evolutionary relationships or to analyze differences within a certain protein class, it might be beneficial to use a time-consuming but accurate method. In case of sites with chemically diverse ligands, interaction-based methods that generate ligand-dependent interaction patterns should not be applied.

Unfortunately, the significance of obtained similarities and reasonable score ranges are not discussed in detail for most methods in literature and no guidance is available. The ability of the applied scoring schemes to discriminate between active (i.e. similar) and inactive (i.e. dissimilar) cavity pairs was also analyzed using data set 1. This is vital to the application of binding site comparison methods. S3–S5 Figs show box plots that illustrate the distribution of scores for similar and dissimilar site pairs. These plots might assist in distinguishing between similar and dissimilar binding site matches in a query-based comparison. The statistical data derived from the Welch’s t-test are given in S4 Table and underline the utility of the different scoring schemes in delimiting similar and dissimilar sites. A clear score-dependent distinction between active and inactive pairs can be observed for the residue-based methods Cavbase, SiteAlign, and TM-align as well as for the surface-based methods ProBiS and SiteHopper. In the publication introducing SiteAlign reasonable cut-off distances for similar binding site pairs for the distance measures d1 and d2 are given[18]. The threshold for d2 (below 0.2) agrees with our data (S3 Fig) for distance d3 differing from d2 only in the center used for distance calculations. The distances between the upper and lower limits of the active and inactive pairs are high for SiteAlign, SMAP, TM-align, and SiteHopper compared to other tools. The structures in this data set are highly similar as the protein structures share the same sequence. Pairs of similar binding sites of unrelated structures will consequently result in lower scores. A large gap between the mean scores of active and inactive pairs is beneficial, as similar but not identical binding sites are likely to yield scores in between these mean values. Therefore, the box plots are useful to estimate meaningful score ranges for similar sites, but should never be misinterpreted as fixed score thresholds applicable for specific binding site comparison projects (see next Results section).

In comparison, the scoring schemes for interaction-based methods do not universally provide such distinct score ranges for similar and dissimilar cavities. IsoMIF and KRIPO performed best among our selection of tools. As explained earlier, the varying ligands of a single binding site are involved in different interactions leading to highly different interaction patterns used for TIFP and Grim comparisons. These narrow score ranges for similar and dissimilar sites were already observed by the method developers[19]. Nevertheless, an interaction-based site matching can lead to interesting findings, e.g. unrelated proteins binding to similar ligands (see below for the data set of Kahraman *et al*.[63]).

In conclusion, the application of interaction-based methods in projects that strive to find similarities between proteins binding to chemically different ligands (e.g. in drug repurposing, off-target prediction) can only be successful as part of an elaborate workflow. The use of binding site prediction, molecular docking, and a detailed visual inspection of the identified similarities are indispensable for TIFP, Grim, IsoMIF, and KRIPO. In contrast, residue- and surface-based methods are the best choice for the elucidation of relationships between sites in complex with different ligands. The scoring metrics for most tools ensure a good discrimination between similar and dissimilar binding site pairs. Nevertheless, the use of fast and unspecific, but sensitive methods (RAPMAD, PocketMatch, FuzCav) should be avoided whenever possible. Their high false negative rates also show that a high proportion of similar pairs remain undiscovered by these methods.

#### NMR data set

**Fig 4:**
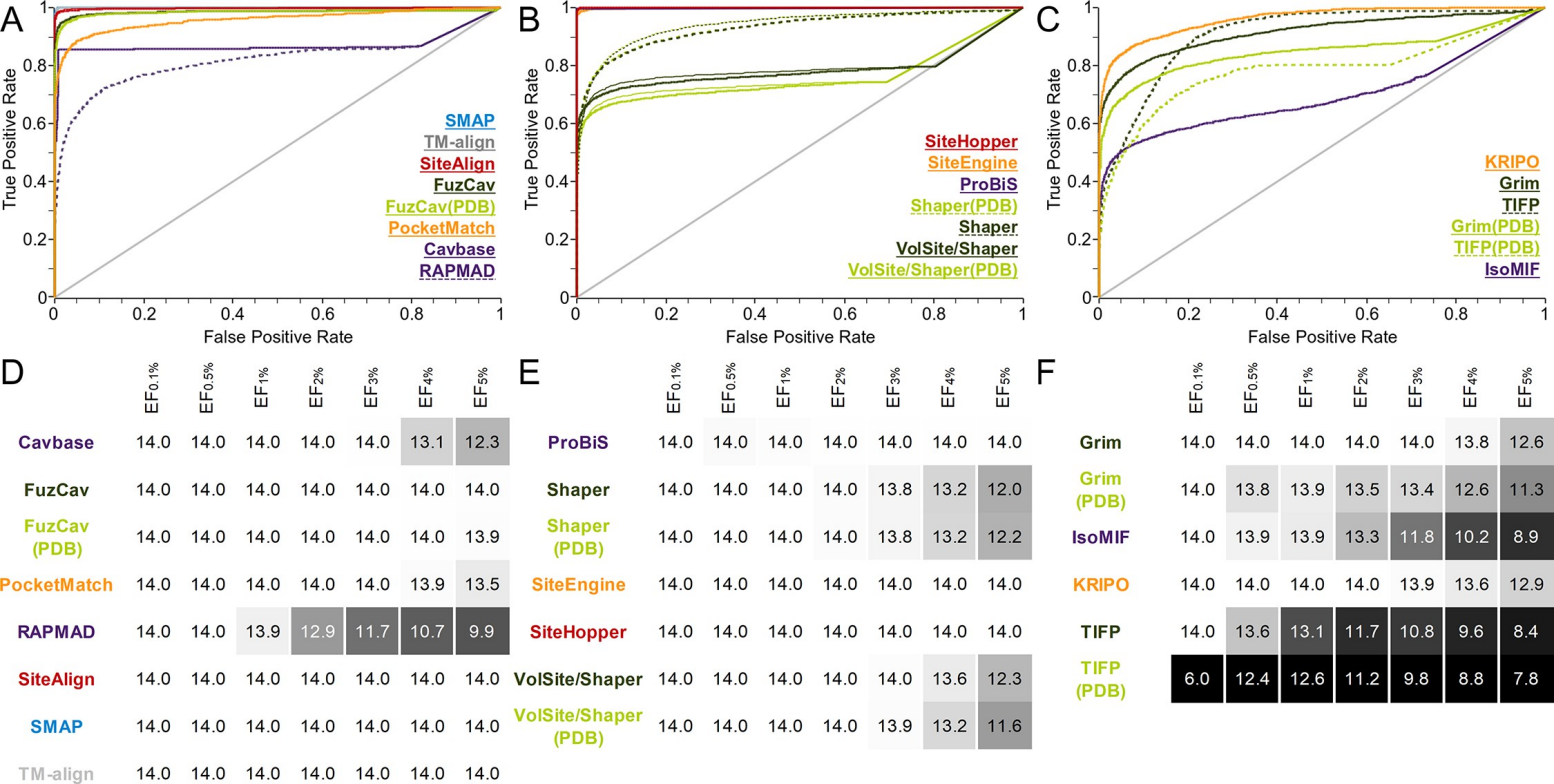
Evaluation of different binding site comparison tools with respect to the data set of NMR structures. A-C) The ROC curves for residue- (A), surface- (B), and interaction-based (C) comparison methods. The name of the tool is colored according to its corresponding ROC curve. The binding site comparison tools are sorted in descending order with respect to the AUC. (A) The highest AUC was obtained for SiteAlign when using distance d1. (B) All Shaper comparisons led to higher AUCs for the scoring measure Tanimoto (color). SiteEngine results slightly improved the AUC for the distance scoring scheme. D-F) EFs for residue- (D), surface- (E), and interaction-based (F) comparison methods. A linear color gradient ranging from white for the highest value to gray to black for the lowest value was applied for the EFs at different percentages of screened data set.

A detailed examination of Fig 3 reveals that differing binding site conformations contribute to high false negative rates, in addition to the effect of differing interaction patterns. Induced fit and conformational selection cause changes in side chain orientations and sometimes also changes in the protein’s backbone atom positions[70,71]. Such observations encouraged us to design a second data set for analyzing whether conformational changes influence the outcome of binding site comparison methods. Solution NMR structures provide easy access to a conformational ensembles of protein structures with the same binding sites containing larger and smaller conformational variations. The binding site RMSD (root-mean-square deviation) values are higher than for data set 1 (S2 and S5 Tables). The impact of conformational fluctuations on the performance is lower compared to that of different binding site definitions, as shown in Fig 4. The AUC values for the interaction-based methods Grim, TIFP, and KRIPO as well as for PocketMatch are significantly higher than for data set 1.

Interestingly, Shaper comparisons alone lead to a low early enrichment compared to other surface-based methods. A difference between the use of Shaper and its application in combination with VolSite (VolSite/Shaper) is evident (S19 Table). This result points toward a challenge concerning automated druggability estimation in combination with binding site flexibility, as already observed during the development of automated druggability prediction methods[72,73]. Following the introduction of the principle of binding site druggability[74], i.e. the potential of a cavity to accommodate drug-like small molecules leading to a modulation of protein function, different computational methods were developed for automated structure-based prediction of druggable binding sites[75]. VolSite initially calculates druggability scores for the binding sites of interest. If the score drops below a certain threshold the binding site is regarded as non-druggable. For non-druggable binding sites, no cavities are extracted for comparison purposes. Intriguingly, certain models of one solution NMR ensemble were predicted as druggable whereas others were predicted as non-druggable. This prevented the comparison for some binding site pairs (see failure rates in S10 Fig). The previously described sensitivity of binding site comparison tools toward binding site definition and flexibility also holds true for druggability prediction. Most computational druggability prediction methods are trained based on binding site descriptors, e.g. the hydrophobicity, the number of hydrogen bond donor and acceptor atoms, and various geometric properties. These characteristics fluctuate highly within the NMR ensembles (S6 Table).

The comparably poor performance of Cavbase and RAPMAD (significant AUC differences to all other tools) relates to the fact that binding sites are extracted based on the LIGSITE[76] pocket identification method instead of relying on the ligand’s environment. Therefore, not all cavities of interest are detected and not all are included in the comparison. This represents a major drawback for both methods and is reflected in the final progression of the ROC curve with a linear slope. Nonetheless, both methods show a high early enrichment. They might be beneficial for identifying highly similar binding sites in a drug repurposing or polypharmacology project without ligand knowledge. An exclusion of all pairs that could not be compared by these tools for benchmark analyses results in an overall small performance gain.

Residue- and surface-based tools are characterized by a poor performance for this set. It might, for example, be beneficial to use MD-derived structural ensembles for a screening of a large binding site library with the binding site of interest. In particular, off-target predictions and the analysis of evolutionary relationships should not rely solely on single structures, but structural ensembles reflecting different conformational binding site states.

**Fig 5:**
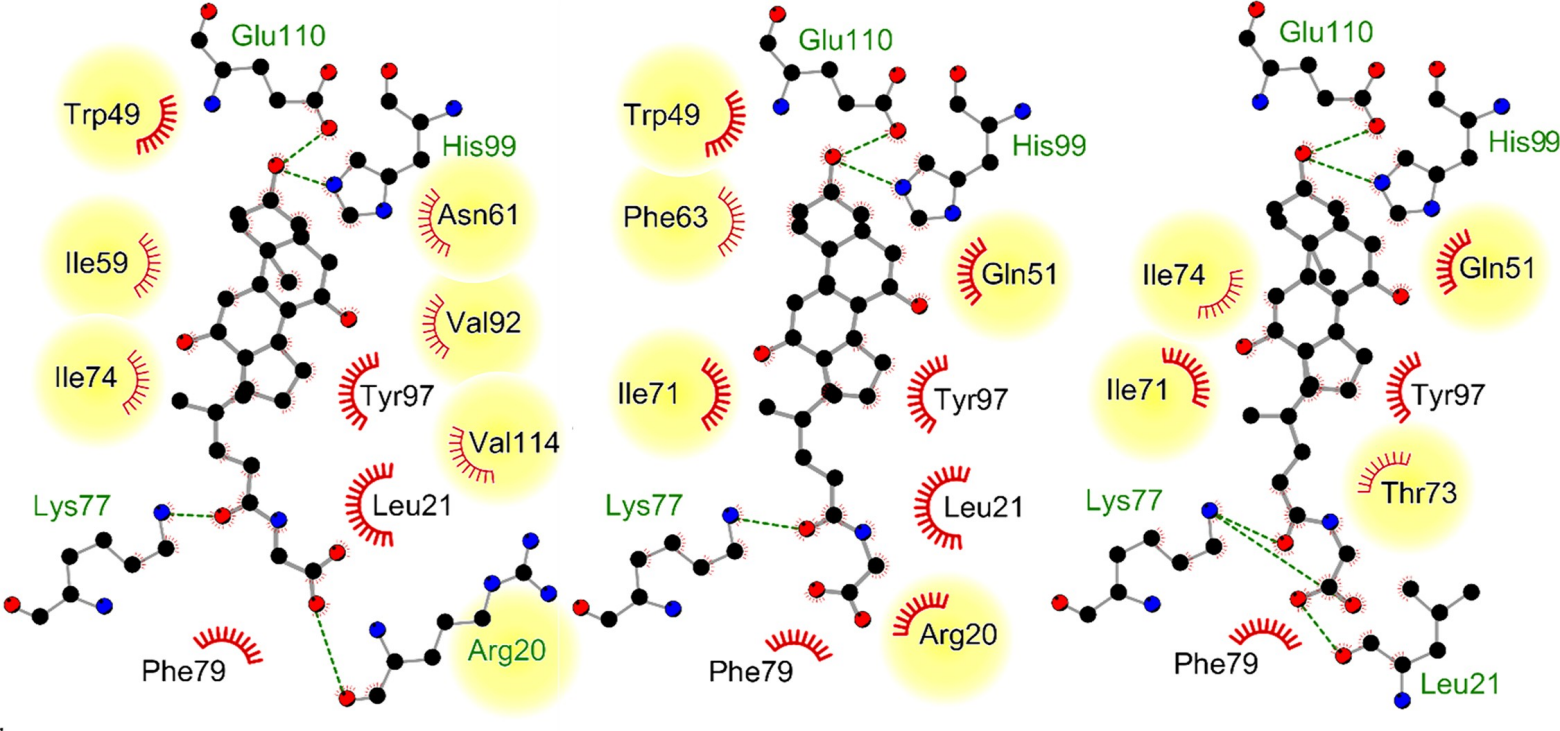
Different interaction patterns for structures from the solution NMR ensemble of ileal lipid-binding protein (PDB ID 1eio, chain A). The ensemble contains five conformers in total. Models 1, 3, and 4 (from left to right) were used to generate this illustration. Residues with alternating interaction patterns in the different conformations are highlighted and occupy nearly half of the pocket. The remaining part of the pocket is mainly engaged in hydrophobic contacts and three hydrogen bond interactions with the small molecule glycocholate. The figure was generated using LigPlot^+^[69].

The same holds true for the interaction-based methods. The question arises of whether unstable interactions of the ligand with the protein of interest vanish in some ensemble structures. The interaction patterns of the underlying NMR ensembles confirm this assumption. Fig 5 exemplifies changes in the binding site environment for three structures of the NMR ensemble of ileal lipid-binding protein. It indicates that only a small portion of all observed interactions remain stable within the ensemble. Such changes do not influence residue- and surface-based methods to a major extent, but they negatively affect comparison methods that rely on a distinct set of interactions. An inclusion of interaction strengths (e.g. as weights) for an interaction-based comparison method could lead to improved overall performance. Again, the inclusion of various protein-ligand complexes for a cavity comparison with an interaction-based tool is essential for success. In particular, the results of IsoMIF are considerably affected by the binding sites’ flexibility. In contrast, KRIPO, which considers the flexibility of binding sites by including fuzziness within the fingerprints, shows a more convincing performance.

Grim and TIFP give similar results with respect to the AUC. The higher early enrichment for Grim relative to TIFP can be attributed to the more complex matching procedure and scoring scheme of the graph-based method. The performance differences between Cavbase and RAPMAD are not very pronounced for the NMR structures when compared to the results for data set 1. Nevertheless, the graph-based method outperforms its faster counterpart in terms of early enrichment.

Taken together, the results for data set 2 indicate the benefits of using a structural ensemble of the protein of interest. For analyses other than query-based investigations (e.g. the calculation of similarity matrices), the user should perform comparisons with tools whose results do not depend on the site conformation (SMAP, TM-align, SiteAlign, SiteEngine, ProBiS). This is the only way of obtaining reliable results in the absence of knowledge about the binding site dynamics.

#### Decoy data sets

The recovery of structures with identical sequences or NMR models does not allow a clear distinction between the comparison algorithms. The question arises whether the algorithms analyzed are able to distinguish between pairs of similar binding sites and decoy structures containing several residue substitutions leading to different physicochemical and geometrical properties. Data set 1 provided the basis for these benchmark data sets. One structure from each group of structures with identical sequences was chosen and one to five residues were substituted by similarly sized residues with different physicochemical properties (decoy structures in data set 3) or by residues whose number of carbon and hetero atoms differs by at least three atoms (decoy structures in data set 4). For the different methodologies, we aimed at investigating whether pairs of binding sites with identical sequences are ranked high while pairs consisting of an original structure and a decoy structure receive lower rankings.

**Fig 6:**
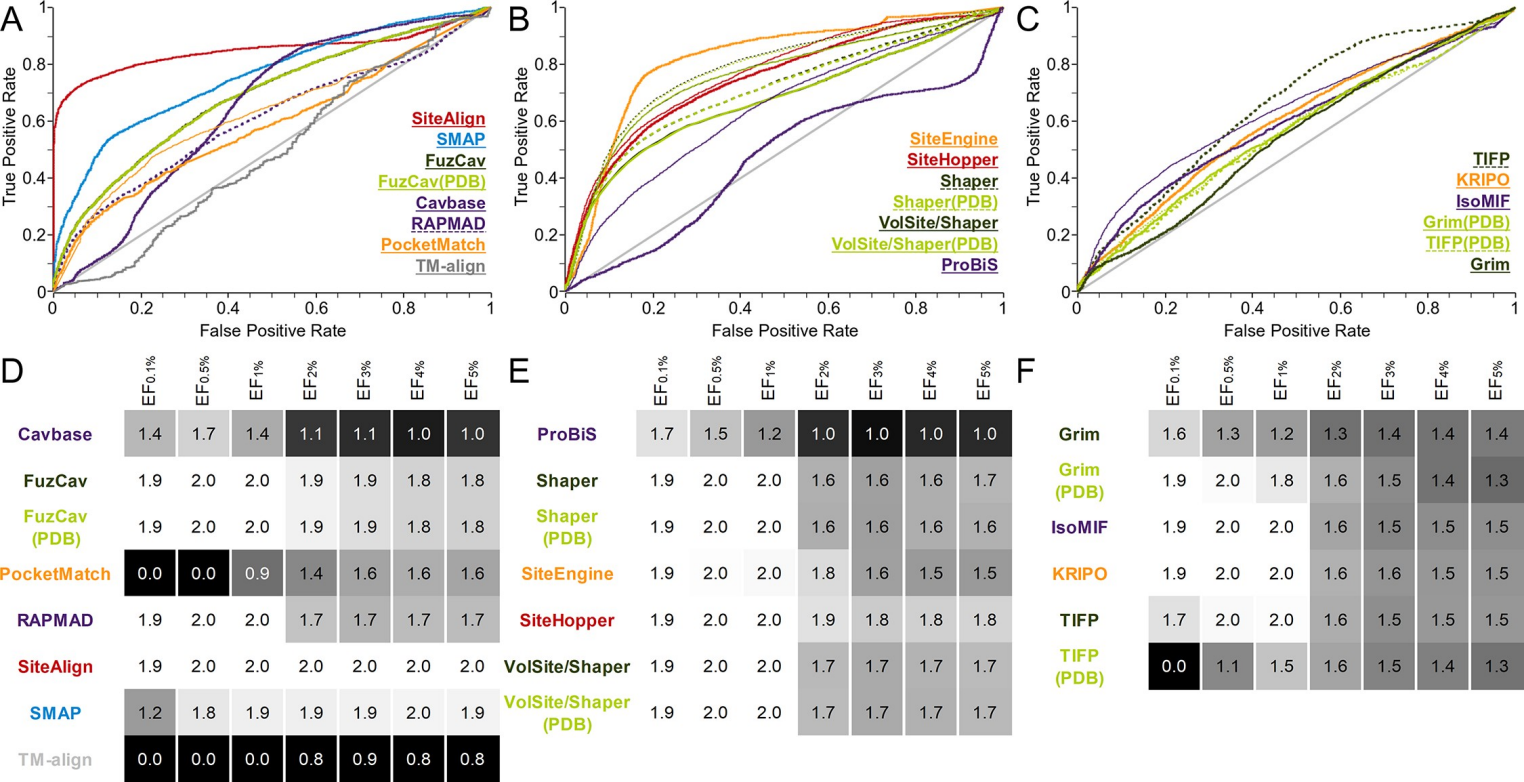
Evaluation of different binding site comparison tools with respect to data set 3 (five substitutions by physicochemically different residues). A-C) The ROC curves for residue- (A), surface- (B), and interaction-based (C) comparison methods. The name of the tool is colored according to its corresponding ROC curve. The binding site comparison tools are sorted in descending order with respect to their AUC. (A) PocketMatch showed the best AUC for the score PMScore_min_ (thin orange line). (B) The scores SVA, RefTversky (color), RefTversky (color), RefTversky (color), RefTversky (color), and ColorTanimoto led to the highest AUC values for ProBiS, Shaper, Shaper(PDB), VolSite/Shaper, VolSite/Shaper(PDB), and SiteHopper, respectively (thin lines). (C) The highest AUC was obtained for IsoMIF and TIFP(PDB) when using taniM and the Tanimoto coefficient as similarity measure (thin lines). D-F) EFs for residue- (D), surface- (E), and interaction-based (F) comparison methods. A linear color gradient ranging from white for the highest value to gray to black for the lowest value was applied for the EFs at different percentages of screened data set.

For data set 3 (different physicochemical properties), decoy binding site variants with one, two, three, four, or five substitutions were generated (see Methods section for more details). Due to the poor performance of the tools for comparisons of the original structures to all possible variants (S6 Fig), we restricted our discussions of the ROC curves and EFs to comparisons with five residue variants. The results are depicted in Fig 6.

Intriguingly, the methods SiteAlign, SMAP, SiteEngine, SiteHopper, and Shaper were significantly (S21 Table) superior to all interaction-based methods in terms of the AUC values. The early enrichment of active pairs is comparably high, although the high early enrichment of active pairs for SiteAlign is unique. The results for TM-align are explicable when considering that the method returns a score that is independent of the type of matched residues. Cavbase, Grim, ProBiS, and TIFP showed the lowest early enrichment. When taking one, two, three, four, and five residue substitutions into account this trend becomes even more pronounced (S6 Fig). Obviously, these tools assign high similarity scores to matches between the original structure and the corresponding decoy structures whereas other structures with different binding site conformations, but identical residues are regarded as being more dissimilar.

Next, we evaluated whether the methods are able to rank the matches between the structure and its variants with respect to the number of substitutions. To this end, we calculated the Spearman’s Rho rank correlation coefficients between the number of substitutions and the corresponding similarity scores for all comparisons between the original structure and its decoy variants (Table 3). The results for TM-align were omitted as the method is not able to distinguish between the original structure and its variants. Negative correlation coefficients indicate a decreasing similarity score with increasing number of mutations. This is to be expected for binding site comparison tools which are able to discriminate between binding sites with different physicochemical properties. The value -1 indicates a perfect correlation.

**Table 3:** The Spearman’s Rho correlation coefficients for a comparison between the original crystal structures and their corresponding decoy structures with one, two, three, four, and five substitutions of randomly chosen residues by physicochemically different ones for residue-based, surface-based, and interaction-based binding site comparison methods. A color gradient was applied to highlight a prominent negative correlation (green), a correlation coefficient of -0.5 (yellow), and no correlation at all or a positive correlation (red). No cavities could be extracted for Cavbase and RAPMAD comparisons for decoy structures generated from the structure with the PDB ID 4ca7 (n.d.).

residue-based methods							
PDB ID.chain	Cavbase	FuzCav	FuzCav (PDB)	PocketMatch	RAPMAD	SiteAlign	SMAP
1kmv.A	-0.90	-0.90	-0.90	-0.31	-0.58	-0.95	-0.89
1odm.A	-0.79	-0.90	-0.90	-0.34	-0.71	-0.89	-0.91
2qxw.A	-0.67	-0.79	-0.79	-0.53	-0.61	-0.75	-0.81
3f17.A	-0.67	-0.87	-0.87	-0.59	-0.69	-0.88	-0.93
3rm2.H	-0.53	-0.58	-0.58	-0.52	-0.60	-0.81	-0.80
3t10.A	-0.72	-0.56	-0.57	-0.52	-0.65	-0.75	-0.92
3u5l.A	-0.66	-0.83	-0.83	-0.59	-0.78	-0.88	-0.87
3u9w.A	-0.86	-0.72	-0.72	-0.33	-0.71	-0.90	-0.82
4bfz.A	-0.70	-0.68	-0.68	-0.31	-0.54	-0.71	-0.84
4buu.A	-0.63	-0.72	-0.72	-0.56	-0.75	-0.76	-0.92
4ca7.A	n.d.	-0.69	-0.69	-0.48	n.d.	-0.84	-0.96
4fpt.A	-0.65	-0.86	-0.86	-0.57	-0.61	-0.85	-0.94
surface-based methods							
PDB ID.chain	ProBiS	Shaper	Shaper (PDB)	SiteEngine	SiteHopper	VolSite/ Shaper	VolSite/ Shaper (PDB)
1kmv.A	-0.53	-0.89	-0.89	-0.76	-0.90	-0.91	-0.91
1odm.A	-0.01	-0.59	-0.59	-0.71	-0.95	-0.92	-0.92
2qxw.A	-0.26	-0.71	-0.70	-0.76	-0.85	-0.73	-0.69
3f17.A	-0.70	-0.69	-0.69	-0.71	-0.77	-0.70	-0.71
3rm2.H	-0.52	-0.60	-0.60	-0.62	-0.79	-0.50	-0.50
3t10.A	-0.31	-0.65	-0.64	-0.66	-0.82	-0.63	-0.62
3u5l.A	-0.54	-0.67	-0.67	-0.65	-0.94	-0.72	-0.73
3u9w.A	0.02	-0.72	-0.72	-0.81	-0.84	-0.67	-0.67
4bfz.A	-0.38	-0.73	-0.74	-0.81	-0.90	-0.77	-0.77
4buu.A	-0.40	-0.73	-0.71	-0.65	-0.90	-0.65	-0.69
4ca7.A	0.22	-0.31	-0.32	-0.75	-0.87	-0.63	-0.63
4fpt.A	0.43	-0.83	-0.82	-0.85	-0.93	-0.85	-0.85
interaction-based methods							
PDB ID.chain	Grim	Grim (PDB)	IsoMIF	KRIPO	TIFP	TIFP (PDB)	
1kmv.A	-0.70	-0.72	-0.56	-0.67	-0.35	-0.70	
1odm.A	-0.66	-0.57	-0.72	-0.77	-0.64	-0.61	
2qxw.A	-0.73	-0.62	-0.60	-0.75	-0.62	-0.57	
3f17.A	-0.58	-0.53	-0.52	-0.75	-0.59	-0.28	
3rm2.H	-0.61	-0.37	-0.68	-0.72	-0.42	-0.28	
3t10.A	-0.46	-0.42	-0.56	-0.61	-0.30	-0.43	
3u5l.A	-0.60	-0.56	-0.24	-0.68	-0.45	-0.59	
3u9w.A	-0.68	-0.73	-0.65	-0.73	-0.50	-0.54	
4bfz.A	-0.51	-0.50	-0.61	-0.51	-0.45	-0.47	
4buu.A	-0.57	-0.32	-0.20	-0.65	-0.37	-0.32	
4ca7.A	-0.67	-0.43	-0.79	-0.63	-0.44	-0.41	
4fpt.A	-0.72	-0.76	-0.40	-0.80	-0.54	-0.61	

This analysis shows that SMAP, SiteHopper, and KRIPO as residue-, surface-, and interaction-based methods are best suited to rank binding site pairs according to a decreasing physicochemical similarity. For Cavbase and RAPMAD, no results were obtained for the structure with the PDB ID 4ca7 (chain A) as the implemented LIGSITE[76] algorithm for binding site processing was not able to detect this buried binding site (S7 Fig).

The results for some variants obtained with IsoMIF show a very weak correlation between the number of mutations and the similarity score. The results are comparable to those of TIFP and Grim which were outperformed by IsoMIF in the two previously discussed data sets. The same holds true for PocketMatch and ProBiS. In some cases, no correlation at all can be observed. The difficulties in the differentiation between original and decoy structures might arise from the isosteric substitutions which retain the overall binding site shape.

**Fig 7:**
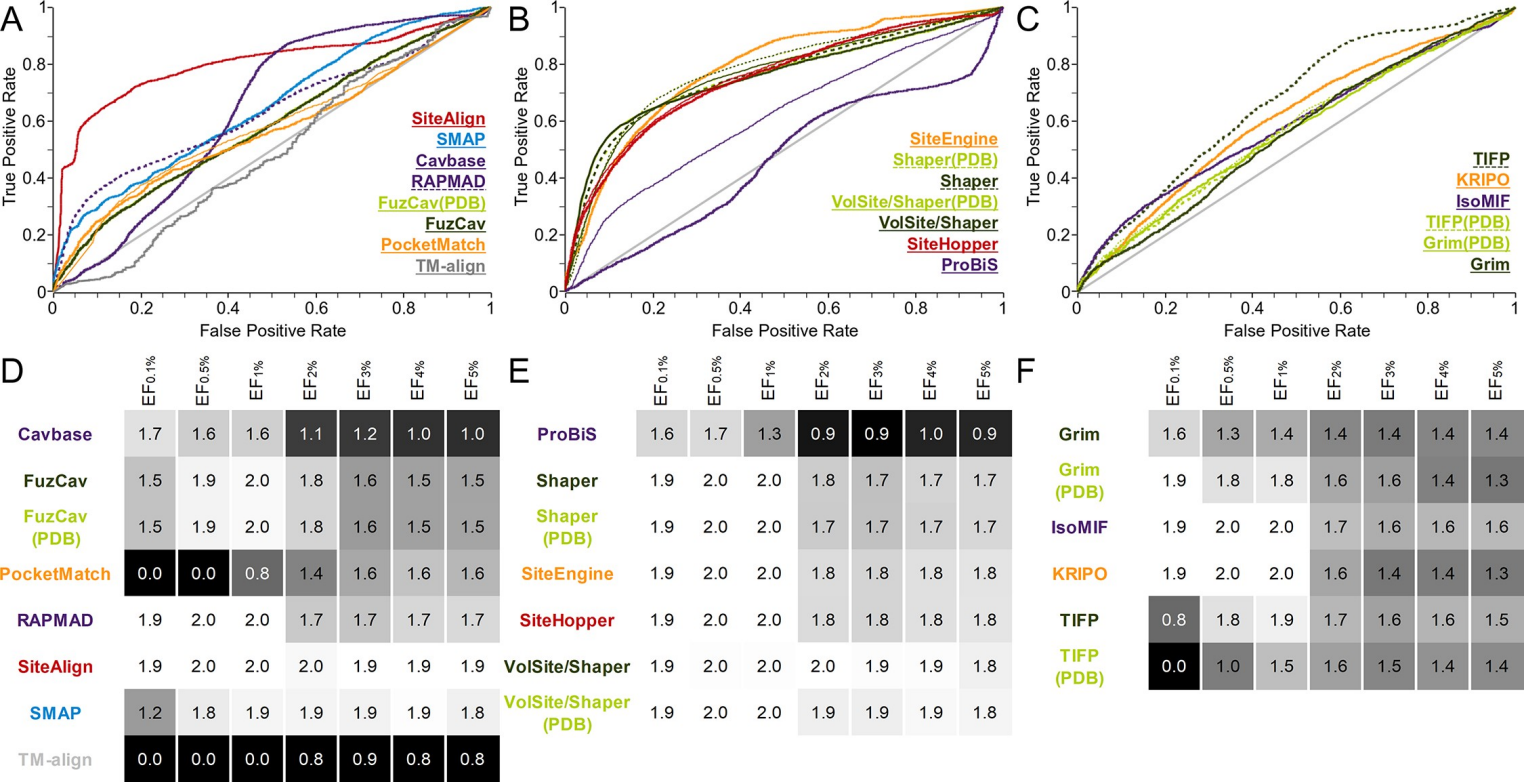
Evaluation of different binding site comparison tools with respect to the data set of rational decoy structures (five mutations). A-C) The ROC curves for residue- (A), surface- (B), and interaction-based (C) comparison methods. The name of the tool is colored according to its corresponding ROC curve. The binding site comparison tools are sorted in descending order with respect to their AUC. (A) PocketMatch showed the best AUC for the score PMScore_min_ (thin orange line). (B) The scores SVA, Tanimoto (color), Tanimoto (color), RefTversky (color), RefTversky (color), and ColorTanimoto led to the highest AUC values for ProBiS, Shaper, Shaper(PDB), VolSite/Shaper, VolSite/Shaper(PDB), and SiteHopper, respectively (thin lines). (C) The highest AUC was obtained for TIFP(PDB) when using the Tanimoto coefficient as scoring measure (thin dark green line). D-F) EFs for residue- (D), surface- (E), and interaction-based (F) comparison methods. A linear color gradient ranging from white for the highest value to gray to black for the lowest value was applied for the EFs at different percentages of screened data set.

We therefore created a second data set of decoy binding sites to evaluate the influence of cavity shape on the final outcome of this study. Randomly chosen residues were replaced by residues with a different shape (data set 4, see Methods section for details). The final outcome is depicted in Fig 7. A general trend in comparison to data set 3 is that most surface-based methods are characterized by a slightly better performance. This holds especially true for Shaper and VolSite/Shaper. The introduction of differently sized residues causes these improvements. In contrast, the performance of ProBiS and the interaction-based methods did not improve. Strikingly, the performance of SMAP is worse than for data set 3. This tool seems to be more sensitive toward the residues’ chemical properties than toward shape. A plausible explanation is that SMAP models geometric binding site features based on the Cα coordinates which do not change upon the introduction of mutations. The superiority of SiteAlign compared to all other tools with respect to early enrichment is also pronounced for this data set. The poor correlation between score and number of substitutions observed for PocketMatch, ProBiS, Grim, IsoMIF, and TIFP did not improve despite the introduction of differently sized residues with modified chemical properties (Table 4).

**Table 4:** The Spearman’s Rho correlation coefficients for a comparison between the original crystal structures and their corresponding decoy structures with one, two, three, four, and five rational mutations of randomly chosen residues for residue-based, surface-based, and interaction-based binding site comparison methods. A color gradient was applied to highlight a prominent negative correlation (green), a correlation coefficient of -0.5 (yellow), and no correlation at all or a positive correlation (red). No cavities could be extracted for Cavbase and RAPMAD comparisons for decoy structures generated from the structure with the PDB ID 4ca7 (n.d.).

residue-based methods							
PDB ID.chain	Cavbase	FuzCav	FuzCav (PDB)	PocketMatch	RAPMAD	SiteAlign	SMAP
1kmv.A	-0.89	-0.67	-0.67	-0.55	-0.52	-0.83	-0.90
1odm.A	-0.94	-0.73	-0.73	-0.31	-0.66	-0.54	-0.92
2qxw.A	-0.68	-0.69	-0.69	-0.19	-0.57	-0.74	-0.93
3f17.A	-0.40	-0.55	-0.55	-0.48	-0.55	-0.82	-0.89
3rm2.H	-0.60	-0.60	-0.60	-0.66	-0.61	-0.73	-0.90
3t10.A	-0.77	-0.44	-0.44	-0.57	-0.61	-0.59	-0.92
3u5l.A	-0.53	-0.62	-0.62	-0.65	-0.32	-0.64	-0.92
3u9w.A	-0.87	-0.79	-0.79	-0.40	-0.52	-0.77	-0.88
4bfz.A	-0.89	-0.28	-0.28	-0.52	-0.77	-0.82	-0.89
4buu.A	-0.72	-0.63	-0.63	-0.38	-0.50	-0.52	-0.92
4ca7.A	n.d.	-0.57	-0.57	-0.49	n.d.	-0.81	-0.90
4fpt.A	-0.79	-0.61	-0.61	-0.51	-0.36	-0.51	-0.88
surface-based methods							
PDB ID.chain	ProBiS	Shaper	Shaper (PDB)	SiteEngine	SiteHopper	VolSite/ Shaper	VolSite/ Shaper (PDB)
1kmv.A	-0.74	-0.90	-0.90	-0.85	-0.92	-0.90	-0.90
1odm.A	0.08	-0.86	-0.86	-0.88	-0.95	-0.89	-0.90
2qxw.A	-0.54	-0.73	-0.74	-0.86	-0.87	-0.70	-0.76
3f17.A	-0.18	-0.66	-0.65	-0.79	-0.74	-0.65	-0.64
3rm2.H	-0.34	-0.47	-0.46	-0.60	-0.81	-0.40	-0.40
3t10.A	-0.47	-0.66	-0.66	-0.74	-0.85	-0.49	-0.49
3u5l.A	-0.42	-0.75	-0.79	-0.76	-0.94	-0.84	-0.84
3u9w.A	0.28	-0.73	-0.72	-0.78	-0.81	-0.57	-0.57
4bfz.A	-0.26	-0.70	-0.71	-0.84	-0.93	-0.72	-0.68
4buu.A	-0.58	-0.59	-0.62	-0.85	-0.83	-0.48	-0.47
4ca7.A	0.27	-0.43	-0.44	-0.76	-0.86	-0.46	-0.47
4fpt.A	0.07	-0.80	-0.80	-0.89	-0.86	-0.84	-0.84
interaction-based methods							
PDB ID.chain	Grim	Grim (PDB)	IsoMIF	KRIPO	TIFP	TIFP (PDB)	
1kmv.A	-0.64	-0.64	-0.69	-0.72	-0.50	-0.63	
1odm.A	-0.57	-0.68	-0.79	-0.76	-0.54	-0.65	
2qxw.A	-0.75	-0.66	-0.66	-0.61	-0.54	-0.66	
3f17.A	-0.58	-0.40	-0.54	-0.52	-0.49	-0.37	
3rm2.H	-0.61	-0.43	-0.61	-0.72	-0.51	-0.41	
3t10.A	-0.37	-0.47	-0.62	-0.70	-0.35	-0.32	
3u5l.A	-0.71	-0.48	-0.29	-0.68	-0.50	-0.33	
3u9w.A	-0.70	-0.61	-0.75	-0.74	-0.55	-0.60	
4bfz.A	-0.57	-0.40	-0.77	-0.79	-0.32	-0.33	
4buu.A	-0.62	-0.63	-0.17	-0.74	-0.52	-0.49	
4ca7.A	-0.47	-0.44	-0.51	-0.68	-0.36	-0.43	
4fpt.A	-0.74	-0.68	-0.56	-0.72	-0.70	-0.75	

In summary, a potential applicability domain of SiteAlign is the accurate evaluation of minor binding site differences. This can be of interest for polypharmacology studies and drug repurposing projects as well as for the evaluation of off-target effects. The same holds true for SMAP and SiteHopper although they show a lower early enrichment. These tools do not necessarily require structural ensembles as input as their sensitivity with respect to binding site flexibility is low. Therefore, no additional computational costs arise for the application of the methods. Methods which enable such accurate scoring measures might be suitable choices to post-process potential matches of similar binding sites obtained with the help of a faster, but less accurate binding site comparison tool.

A closer examination of the Spearman’s Rho correlation coefficients (Table 4) shows that the SMAP and SiteHopper scoring schemes are best suited to correlate the degree of physicochemical dissimilarity with the final score. For the interaction-based methods, KRIPO led to the clearest correlation between the number of substitutions and the score. Consequently, the application of these tools might offer an opportunity to correlate ligand affinities with binding site similarities, e.g. for protein kinases[68,77,78]. A clear differentiation between minor dissimilarities is an essential necessity for such analyses and the abovementioned tools might be helpful to reliably rank binding sites according to their similarities. However, if a robust method is needed that should not be influenced by minor dissimilarities, one of the other tools should be used. A potential application is the elucidation of unobvious binding site similarities, as for example shown for SiteAlign[41], ProBiS[49], and KRIPO[57]. Examples for such unapparent similarities are given in the last Results section.

Taken together, the results indicate that several residue-based, surface-based tools and the interaction-based tool KRIPO are best suited to accurately score similarities between binding site pairs with minor modifications. Depending on the desired outcome (identification of minor dissimilarities or the identification of only moderately similar sites), the most suitable method should be chosen with care.

#### Kahraman data set

A commonly analyzed data set for the evaluation of binding site comparison algorithms is the data set of Kahraman and co-workers[63]. It contains several different cofactor sites and small molecule binding cavities (S7 Table). The structures were originally assembled to assess the assumption that ligand shape and binding site shape are related. It was concluded that differently shaped binding sites bind the same ligand, but that shape complementarity might be a significant driver for ligand recognition. We used this data set to find out about the impact of binding site features, interaction patterns, and binding site shape on the recovery of protein binding site pairs binding to identical ligands.

**Fig 8:**
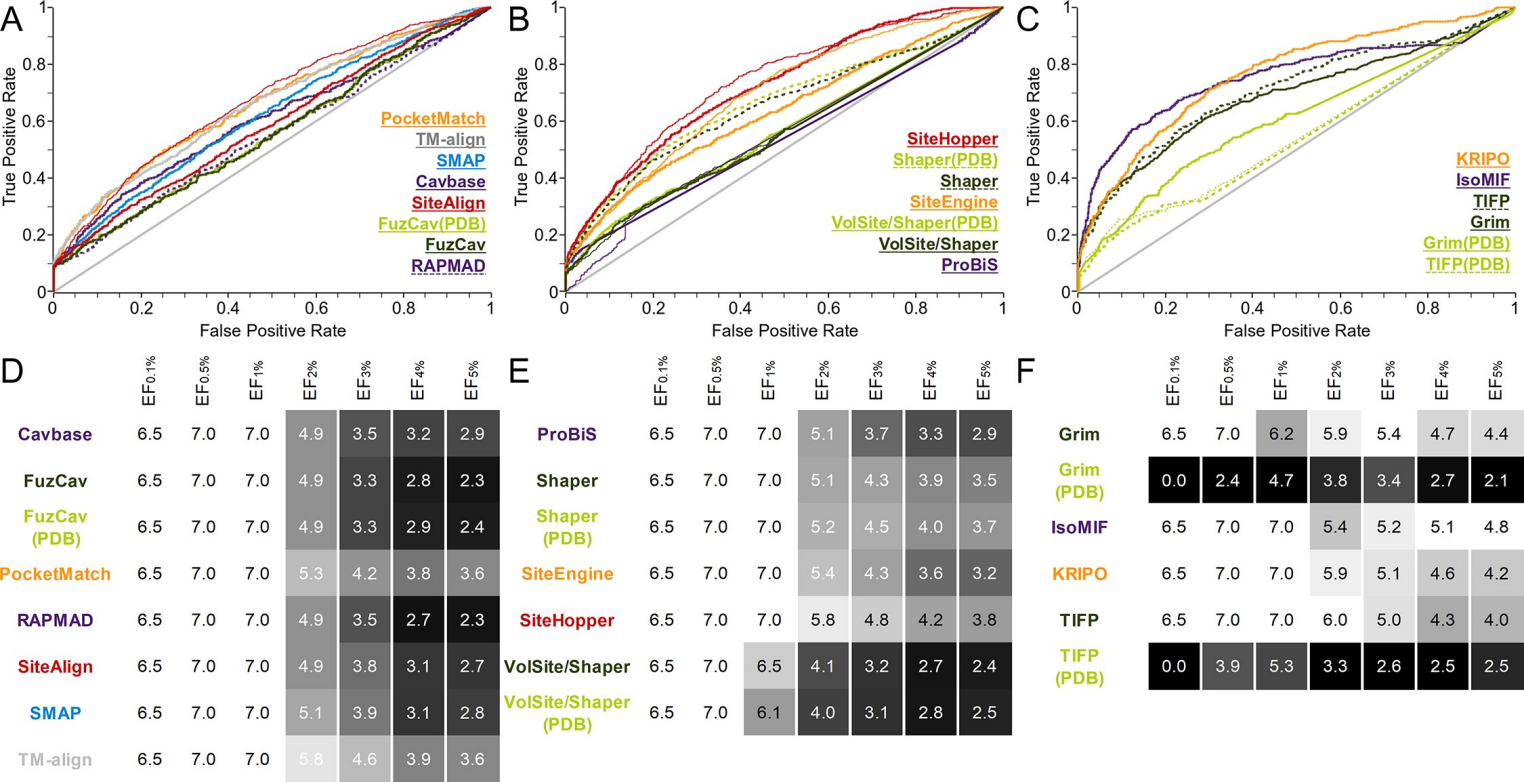
Evaluation of different binding site comparison tools with respect to the data set of Kahraman structures [63] after the exclusion of phosphate binding sites. A-C) The ROC curves for residue- (A), surface- (B), and interaction-based (C) comparison methods. The name of the tool is colored according to its corresponding ROC curve. The binding site comparison tools are sorted in descending order with respect to the AUC. (A) The best AUC for SiteAlign resulted from the d1 distance (thin red line). (B) For ProBiS, VolSite/Shaper, SiteEngine, and SiteHopper the scores SVA, Tanimoto (color), TotalScore, and ShapeTanimoto yielded the best AUC values (thin lines). (C) For TIFP(PDB), the use of the Hamming distance led to the best results with respect to AUC (thin line). D-F) EFs for residue- (D), surface- (E), and interaction-based (F) comparison methods. A linear color gradient ranging from white for the highest value to gray to black for the lowest value was applied for the EFs at different percentages of screened data set.

One problem with this data set occurs during comparisons. Some of the tools failed to process the much smaller phosphate binding sites due to the low number of interactions or binding site residues involved. To analyze the impact of these binding sites on the final outcome, two evaluation steps were conducted: the analysis of 100 vs. 100 structures (S8 Fig) and the analysis of 80 vs. 80 structures (with excluded phosphate binding sites, Fig 8). IsoMIF, SiteHopper, and KRIPO significantly outperform most of the remaining tools. This tendency is less obvious when omitting the phosphate binding site from the analysis. Even similarities between the phosphate binding sites can be identified based on common geometric binding site properties and a high number of positively charged residues (Fig 8). While the performance of residue-based tools is poor, that of surface- and interaction-based methods looks more promising. The interaction-based method KRIPO clearly outperforms all residue- and surface-based methods except SiteHopper and shows a high early enrichment. The early EFs of IsoMIF and KRIPO are the highest for all interaction-based methods if all 100 structures are considered (S8 Fig). Together with SiteHopper, IsoMIF is also the tool with the highest AUC for the binding site pairs of this complete set. This is not unexpected as the tool was validated on and probably optimized for these structure pairs (S1 Table).

**Fig 9:**
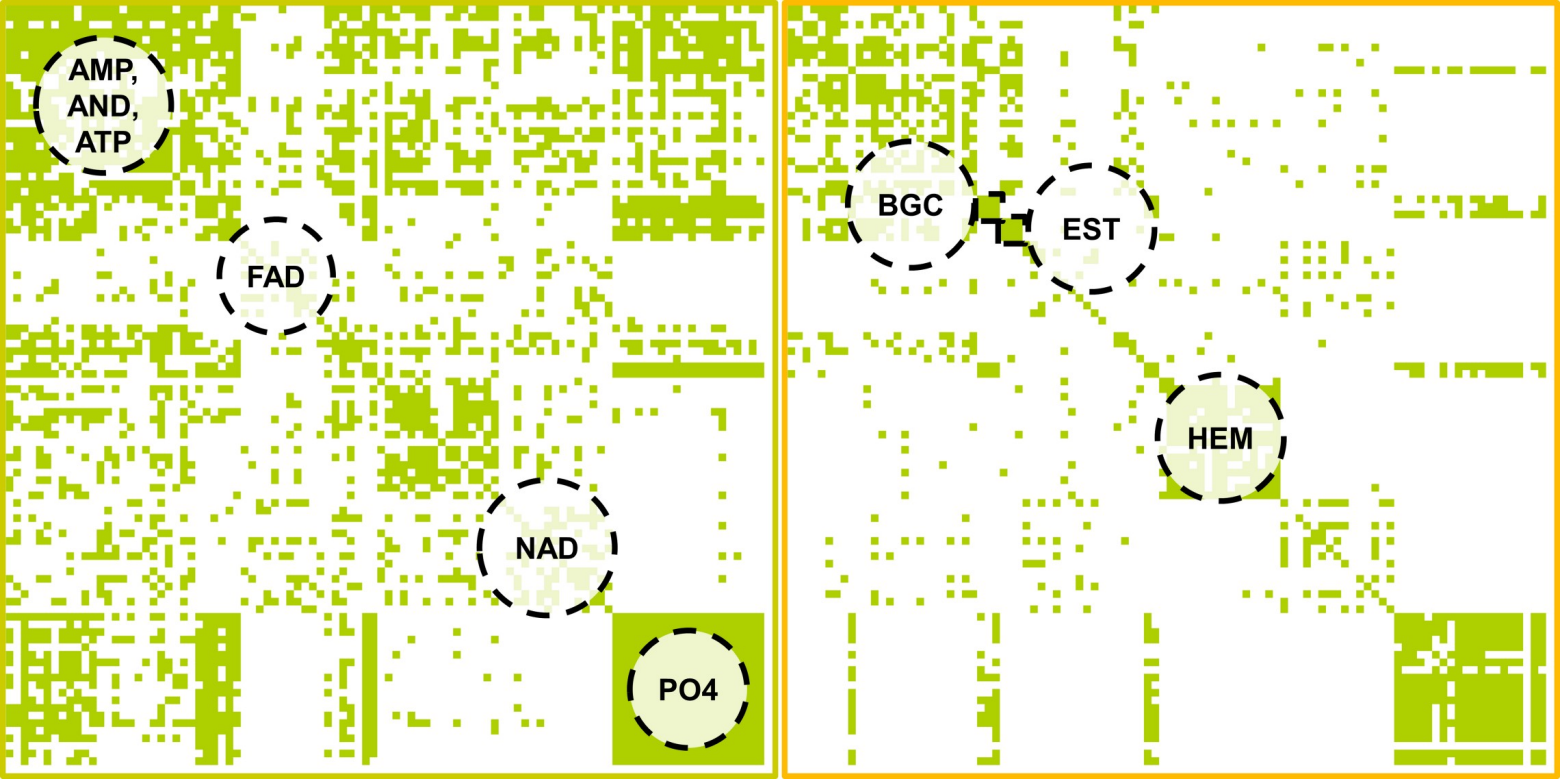
Similarity score matrices for the Kahraman data set generated from the SiteHopper (left) and IsoMIF (right) results. Both methods are able to find clusters of binding sites with identical ligands. The combination of both methods might even give rise to an improved differentiation. Similarity scores (tani) above 0.4 are colored green for the matrix obtained with IsoMIF. Similarity scores (PatchScore) above 0.65 are colored green for all SiteHopper-derived site alignments.

A preliminary conclusion that can be drawn from this analysis is that a combination of surface- and interaction-based methods might be the key for the detection of binding site similarities between otherwise unrelated proteins. The similarity matrix of the all-against-all comparison for IsoMIF and SiteHopper underlines this finding (Fig 9). Both methods do not only successfully differentiate between active and inactive pairs, but they are also suitable for clustering binding sites according to the bound ligands.

#### Barelier data set

A recent publication by Barelier and co-workers[64] focused on similarities and differences between binding sites of unrelated proteins binding to identical ligands (in one case similar ligands). The authors screened the PDB for identical ligands, but included some pre-filtering steps: cofactors as well as ligands found in at least 15 complexes, with a molecular weight above 500 g/mol, or less than 10 non-hydrogen atoms were excluded. The resulting 62 pairs of protein-ligand complexes were classified and divided into three classes according to the similarities of ligand interactions: similar interactions of the ligands with similar protein functionalities (class A), similar interactions of the ligands with different protein functional groups (class B), and different functional groups of the ligands interacting with the proteins (class C). The calculation of per atom van der Waals and electrostatic interaction energies per ligand atom and their subsequent normalization enabled this detailed comparison of ligand-site interactions.

**Fig 10:**
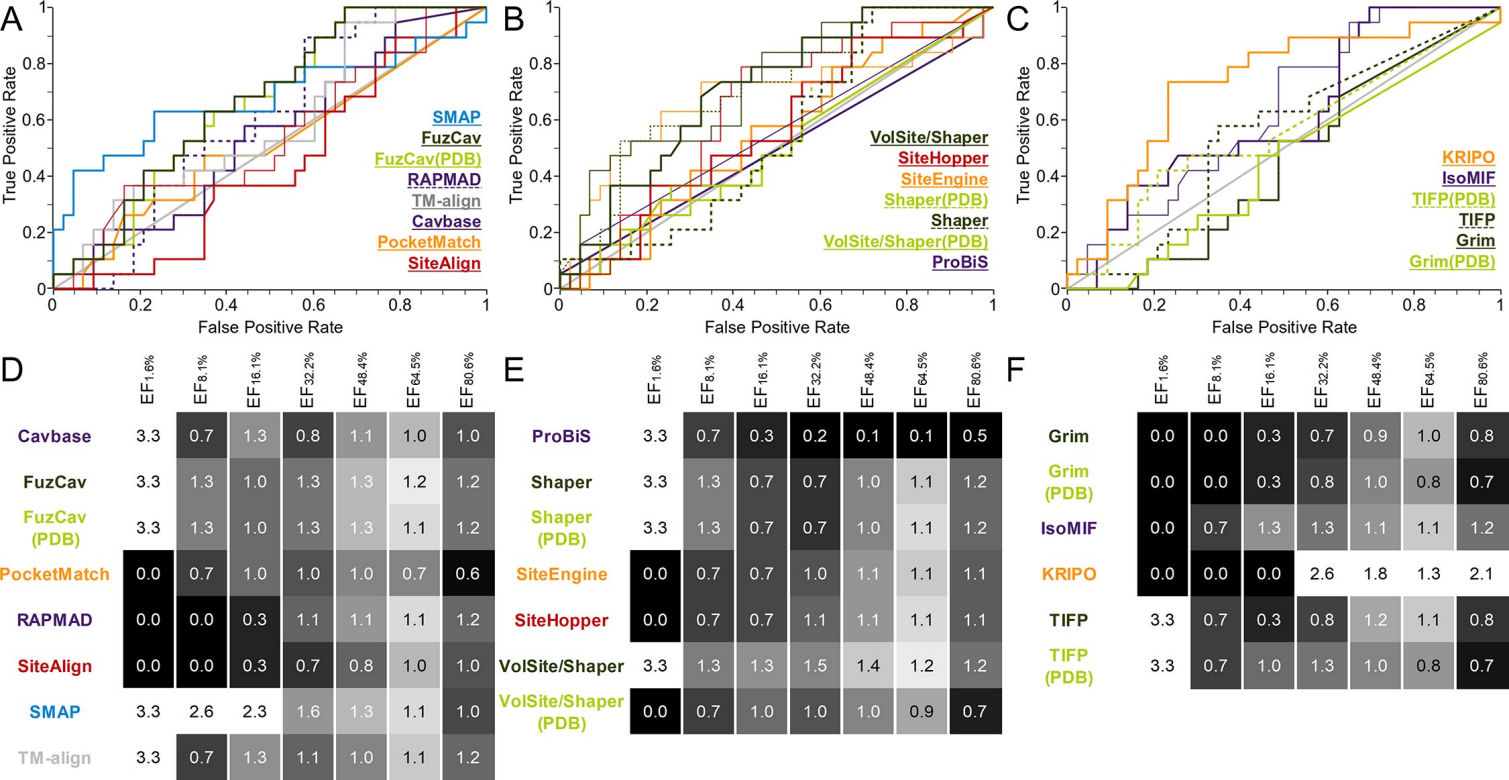
Evaluation of different binding site comparison tools with respect to the data set of Barelier *et al*.[64]. A-C) The ROC curves for residue- (A), surface- (B), and interaction-based (C) comparison methods. The name of the tool is colored according to its corresponding ROC curve. The binding site comparison tools are sorted in descending order with respect to the AUC. (A) The thin red line represents the resulting ROC curve for SiteAlign when using the distance d1. (B) Thin lines represent the ROC curves for ProBiS, Shaper, Shaper(PDB), VolSite/Shaper, VolSite/Shaper(PDB), SiteEngine and SiteHopper when using the scoring schemes SVA, FitTversky (color), FitTversky (color), RefTversky (color), Tanimoto (fit), distance, and ShapeTanimoto, respectively. (C) The thin line represents the resulting ROC curve for IsoMIF and the taniMW score. D-F) EFs for residue- (D), surface- (E), and interaction-based (F) comparison methods. A linear color gradient ranging from white for the highest value to gray to black for the lowest value was applied for the EFs at different percentages of screened data set.

Protein-ligand complexes of class A were used as active pairs in our analysis of all 62 binding site pairs. Due to the small number of protein pairs, EFs for 1.6%, 8.1%, 16.1%, 32.3%, 48.4%, 64.5%, and 80.6% of screened data set were calculated. The results are given in Fig 10. The ROC curves indicate a random ranking of active and inactive pairs for all methods analyzed herein. A significant differentiation between the tools’ AUC values is only possible in a few cases (S25 Table) and the data set size precluded the decision for a suitable tool. The performance improvements using more appropriate scoring measures are also negligible for this data set.

**Fig 11:**
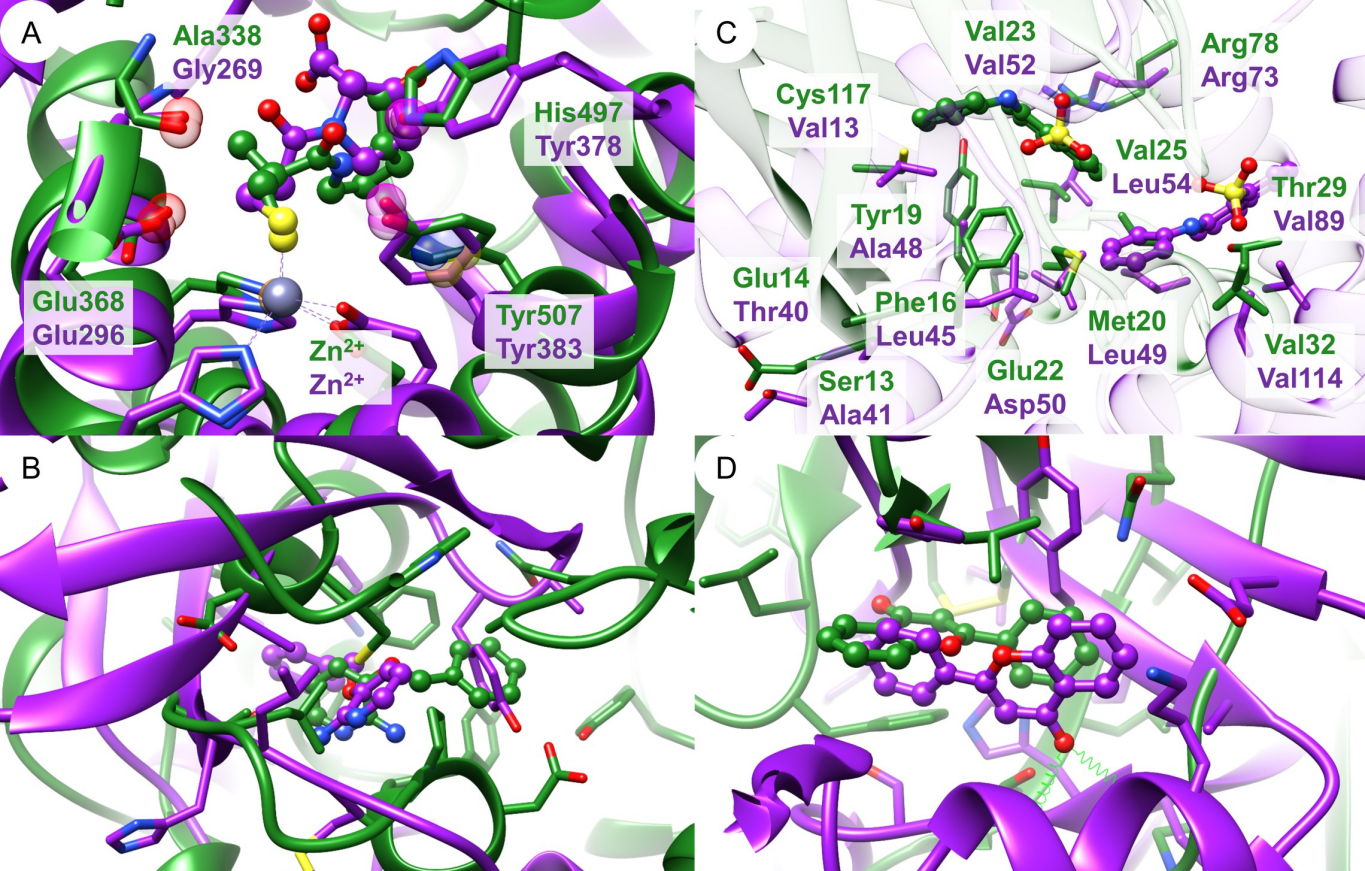
**Alignments of high-scoring binding site pairs of the Barelier data set generated by (A) Cavbase, (B) TM-align, (C) SMAP, and (D) Shaper.** (A) Superposition of angiotensin-converting enzyme (PDB ID 2x8z, green) and leukotriene A4 hydrolase (PDB ID 4dpr, purple) in complex with captopril (ball-and-sticks representation). Red spheres denote hydrogen bond acceptor features while purple spheres represent mixed hydrogen bond acceptor/donor features. Metal coordination sites are marked by orange spheres and blue and yellow spheres denote residues with aromatic and aliphatic characteristics, respectively. The Cavbase similarity score for this match is 11.37. (B) Alignment of leukotriene A4 hydrolase (PDB ID 3fty, green) and mitogen-activated protein kinase 14 (PDB ID 1w7h, purple) crystallized with the small molecule fragment 3-(benzyloxy)-pyridine-2-amine (3IP, ball-and-sticks-representation). Residues within a 4 Å radius of any ligand atom are depicted in stick representation. This alignment yields a TM-score of 0.32. (C) Superposition of adipocyte lipid-binding protein (PDB ID 2ans, green) and pheromone-binding protein (PDB ID 1ow4, purple) in complex with the fluorescent probe 8-anilino-1-naphthalene sulfonate (2AN, ball-and-sticks). The residues shown in stick representation represent only a fraction of all matched residues. The SMAP RawScore for this site pair is 63.44. (D) Shaper-based alignment of the neocarzinostatin (PDB ID 2g0l, green) and tankyrase-2 (PDB ID 4hki, purple) flavone (FLN, ball-and-sticks) binding sites (TanimotoCombo = 0.92). Residues within a 4 Å radius of the ligand are represented as sticks. Hydrogen bond interactions are depicted as green springs.

Based on the per atom score analysis of Barelier *et al*.[64], one might expect that the ligands nicely overlay for high scoring pairs of binding sites. The underlying binding site alignments of class A pairs were therefore analyzed for tools with a high early enrichment and enable the visualization of the underlying match (Cavbase, SMAP, TM-align, ProBiS, and VolSite/Shaper). Fig 11 shows the binding site alignments for some high-scoring site pairs. The best-scored Cavbase match reflects an acceptable overlay of captopril bound to angiotensin-converting enzyme and leukotriene A4 hydrolase. The match between these binding sites was also the highest-scoring of all active pairs for TM-align. A good superposition of the bound identical ligands could be achieved. The second highest ranked true positive match yielded a superposition with poorly aligned ligands. The SMAP alignment of the class A pair with the highest score does not superpose the ligands in a satisfactory manner. Nevertheless, it is obvious that the tool was able to identify residue-based binding site similarities. A closer look shows that the residues taken into account for comparison are rather broadly distributed across the proteins providing a possible explanation for the unsatisfactory superposition. Only one significant (according to the expectation values) match was found by ProBiS for the binding sites of the abovementioned captopril binding proteins with a good agreement of the ligand atom positions in the alignment. Shaper-derived alignments also do not provide perfect alignments of ligand atoms for the best-scored hits, although ligand moieties with a similar physicochemical nature overlap. These results indicate that a visual inspection of the best ranked binding site pairs is a crucial step to assess the significance of the identified matches. Binding site similarity can only be a reasonable explanation for the binding of identical or similar ligands if the corresponding ligand atoms overlap in the superposition.

Sturm and co-workers analyzed the chemical nature of promiscuous ligands. Based on an analysis of the sc-PDB[65] the authors named quinone (PDB ligand-id QUE) as a super-promiscuous ligand[37]. As depicted in Fig 11D, each ligand interacts mainly via hydrophobic interactions with various cavity residues. In the binding site of the structure with the PDB ID 4hki, the ligand is engaged in two additional hydrogen bond interactions with the protein backbone. Given these observations, the question arises whether binding site comparison is the method of choice for such predominantly hydrophobic cavities.

As cofactors and ions were taken into account for the comparison of interactions by Barelier *et al*.[64], we included them to assess their impact on binding site comparison. The final outcome did not lead to significant changes in the obtained results (S9 Fig and S31 Table).

According to our results, the only acceptable match out of all available class A pairs was that between the captopril binding sites of the proteins with the PDB IDs 2x8z and 4dpr. Some further class A pairs were assigned high scores, but the alignment of the corresponding binding sites did not correctly superpose the counterpart ligand atoms, but physicochemically similar ligand moieties (Fig 11D). Some results can be explained by the way similar binding site pairs were extracted. Sometimes, the ligand atoms “observe” similar interaction partners, but the 3D orientation of these atoms is rather different as presented in S8 Table. Nevertheless, this does not thoroughly explain the final outcome.

**Fig 12:**
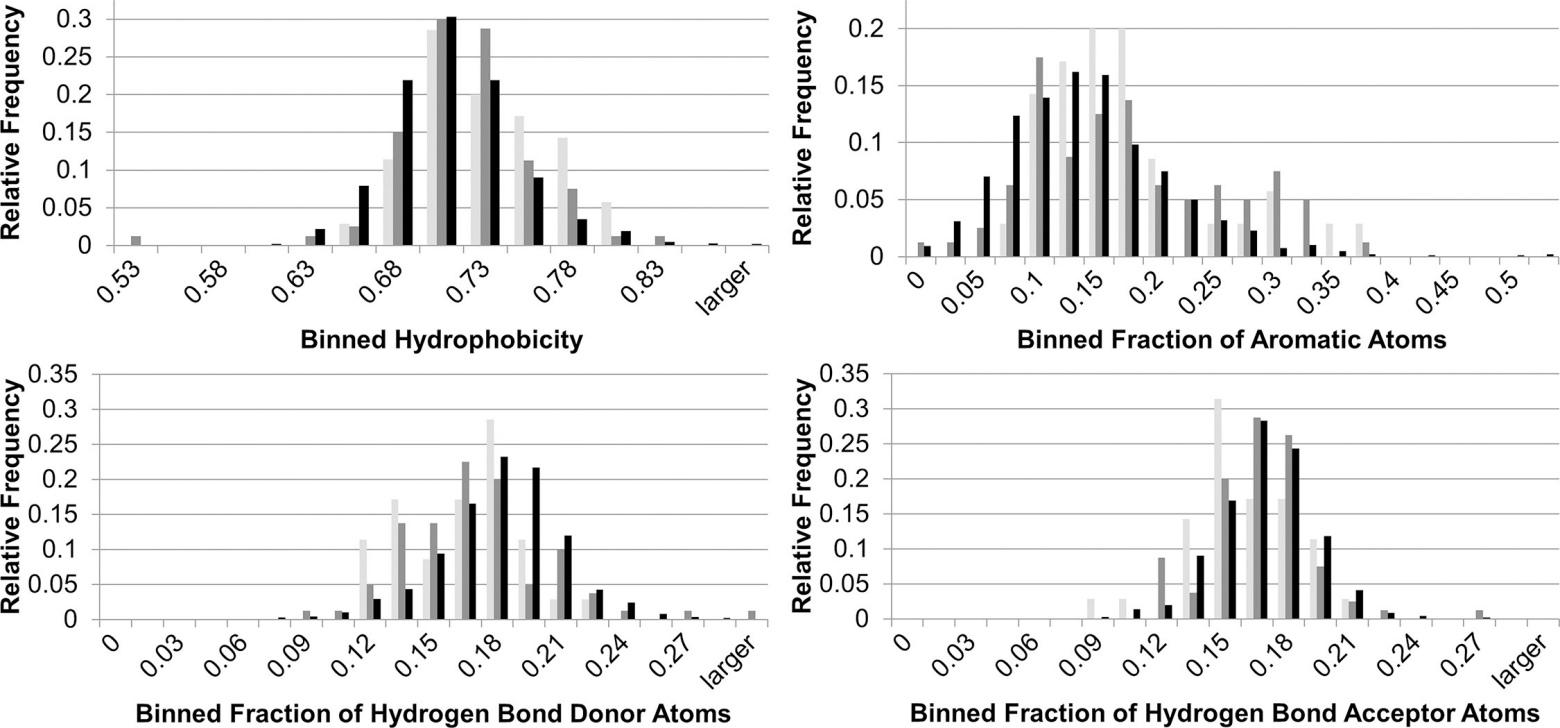
**Results of a binding site feature analysis for the class A, B, and C pairs of Barelier *et al*.[64] and a sequence-culled subset of druggable binding sites.** The relative frequencies of the binned properties are presented in light gray for class A structures, in dark gray for structures belonging to class B and C pairs, and in black for the sequence-culled sc-PDB[65] subset. The binding site features were calculated using DoGSite[73].

The question arises whether different parts of the binding site are insufficiently dissimilar to be clearly distinguished (promiscuous ligand binding sites). A binding site analysis of the structures involved in the different similarity classes and druggable sites of a sequence-culled sc-PDB[65] subset led to the results presented in Fig 12. While the hydrophobicity and aromaticity of the binding sites in the benchmark set is higher than that for the sc-PDB[65] pockets, the number of hydrogen bond donor and acceptor atoms is lower. This underlines the finding that binding site similarity does not always imply a similar binding mode adopted by the ligands. For rather hydrophobic and aromatic ligands, multiple binding modes are possible irrespective of the underlying binding site alignment. Moreover, the generation of an accurate superposition is hampered by the fact that for highly hydrophobic binding sites a broad variety of acceptable cavity alignments can be obtained. We conclude that the generation of a similar data set—taking into account only druggable binding sites with a broad range of interaction types—might be more valuable in the evaluation of the performance of binding site comparison methods.

#### Data set of successful applications

**Fig 13:**
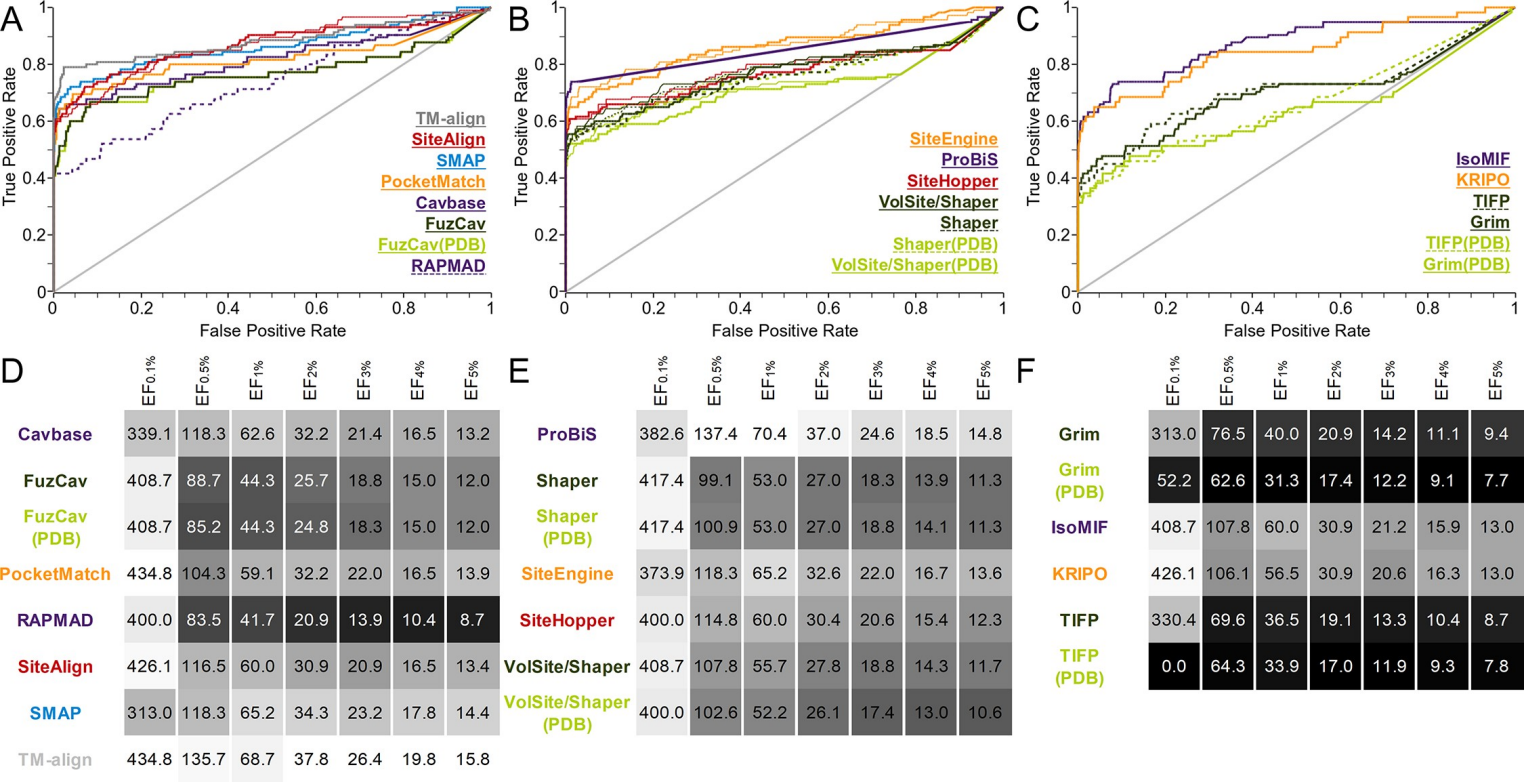
Evaluation of different binding site comparison tools with respect to the data set of successful applications. A-C) The ROC curves for residue- (A), surface- (B), and interaction-based (C) comparison methods. The name of the tool is colored according to its corresponding ROC curve. The binding site comparison tools are sorted in descending order with respect to the AUC. (A) SiteAlign yielded a slightly better AUC if the distance d1 was used (thin line). (B) The best AUC values for ProBis, Shaper, Shaper(PDB), VolSite/Shaper, VolSite/Shaper(PDB), SiteEngine, and SiteHopper resulted from the scoring measures Zscore, Tanimoto (color), Tanimoto (color), Tanimoto (color), Tanimoto (color), TotalScore, and ShapeTanimoto, respectively (thin lines). D-F) EFs for residue- (D), surface- (E), and interaction-based (F) comparison methods. A linear color gradient ranging from white for the highest value to gray to black for the lowest value was applied for the EFs at different percentages of screened data set.

The last data set was created from known binding site similarities that have been summarized in literature[5]. Cavities with known similarities were included in a sequence-culled sc-PDB[65] derived cavity subset. Their recovery within the best ranked pairs was analyzed. As compared to the previous data sets, the active pairs represent realistic binding site similarities that should be found and respectively scored by all comparison methods. Besides obvious similarities between binding sites of one protein family (e.g. protein kinases), similar pairs of binding sites in unrelated proteins are included. The outcome of this analysis is shown in Fig 13 and puts the results obtained for all above analyzed data sets into perspective. The differences between the different types of binding site comparison tools cancel each other out for this data set. In most cases, there are only minor differences in the AUC values for the methods and most of these differences are not significant (S33 Table). All tools show a good performance in terms of AUC as well as in terms of early enrichment.

Most residue-based methods outperform the surface- and interaction-based tools, which were the best among all tools for the data set of Kahraman and co-workers. TM-align–a tool which cannot be used to score minor physicochemical binding site differences and aligns residues exclusively based on geometric criteria–is the one with the highest AUC and the highest early enrichment. This underlines a specific challenge for binding site comparison. Although software should be able to accurately score similarities, it also has to allow for the observation of non-obvious similarities. Additionally, the difference in performance between Cavbase and RAPMAD is very pronounced for this set of lifelike protein pairs. Obviously, a purely histogram-based comparison enriches dissimilar pairs together with the similar ones.

Surface- and interaction-based tools are also characterized by a high early enrichment of known similar pairs. SiteEngine and ProBiS are the surface-based methods which are best suited to recover known similarities in this set. Despite the huge difference in their ability to score site similarities according to the number of substituted residues (data sets 3 and 4), both tools perform similarly in this analysis underlining the necessity to choose a binding site comparison tool based on the user’s requirements. This analysis does not aim for the identification of the best method, but emphasizes the impact of the study design on the final decision. For example, while ProBiS can be safely applied to perform query-based searches for similar binding sites (e.g. for function prediction), SiteEngine is well suited to perform all-against-all comparison between binding sites of similar proteins to elucidate relationships and identify potential off-targets.

With regard to the interaction-based methods, the clear superior performance of IsoMIF and KRIPO that was present for other data sets becomes even more evident here. This trend might change upon exclusion of binding sites that could not be processed for a comparison with Grim and TIFP which perform similarly good for this set. Although all interaction-based methods show a comparable early enrichment, KRIPO and IsoMIF seem to be the methods of choice for applications which originate from already known protein-ligand complexes. This relates with the finding that the worse performing methods Grim and TIFP fail to identify similar sites in complex with different (Tc < 0.6) ligands.

### Will the similar sites please stand up?

#### Similarity cut-off values

Another topic of interest, which was already discussed with respect to the benchmark analyses of data set 1, is the definition of appropriate score cut-offs to differentiate between similar and dissimilar binding sites. The challenges and data set dependence of similarity cut-off values were exhaustively discussed for small molecule similarities[79–81]. These analyses in cheminformatics are supported by the availability of bioactivity data[82,83]. In consequence, some fingerprint similarity thresholds to distinguish between similar and dissimilar molecules could be derived[80]. In contrast, this data is hard to extract for small molecule binding sites. The site of action of molecules with known bioactivities is often difficult to spot experimentally in bioassays. Especially enzyme activity assays provide no hints at the modulator’s binding site. For most known small molecule modulators, only the respective target is reported in publicly available databases such as ChEMBL[84].

The definition of similarity of binding sites relies on structural data retrieved from the PDB[1] and comparative modeling studies. The investigations of Barelier and co-workers[64] underline the sparseness of this crucial data resource. The evaluation of binding site similarities is therefore restricted to well-known binding sites (e.g. cofactor binding sites), artificially created data set (e.g. the decoy sets applied in this study), and already known binding site similarities. These factors impede the evaluation of comparison methods and the definition of a reliable threshold for each method. Additionally, the problem of deriving reliable cut-off values is still up for discussion. Youden’s index[85], the F-measure, likelihoods[86], or discriminant power[87] are only a few examples for discriminant measures.

We employed the ROC-based Youden’s J statistic[85] to find optimum cut-off scores to distinguish similar and dissimilar site pairs for each method. This analysis was first restricted to data set 1 to define a unique threshold per tool. The application of these thresholds for the data set of successful applications leads to the results presented in columns two to five of Table 5. While all tools except RAPMAD are able to reliably reject dissimilar binding site pairs with the given cut-offs (high specificity), the maximum sensitivity is 0.73. Similar binding site pairs seem to yield similarity scores below the predefined thresholds and are therefore rejected. PocketMatch and RAPMAD are within the three methods with the highest sensitivity for the complete data set.

**Table 5:** Sensitivity (se) and specificity (sp) values for data set 7 using different score cut-offs. The score thresholds to discriminate similar and dissimilar site pairs were determined using the ROC-based Youden’s J statistic[85] based on data set 1 and data set 7. Both thresholds were applied for sensitivity and specificity calculations. The rank column gives the rank of the respective method within all methods for the corresponding sensitivity. Sensitivity and specificity values for thresholds that were assigned by the methods’ developers are given in brackets. For SiteAlign, Shaper, VolSite/Shaper, and TIFP, the corresponding scoring measure is different from the one used in our study.

Method	score cut-off based on data set 1 (in brackets cut-off provided by developers)				score cut-off based on data set 7			
	cut-off	sp	se	rank (se)	cut-off	sp	se	rank (se)
Pocket-Match	0.16	0.86	0.73	1	0.26	0.95	0.70	6
SMAP	69.18	0.97	0.71	2	67.61	0.96	0.72	4
RAPMAD	0.89	0.51	0.71	3	0.97	1.00	0.42	19
TM-align	0.49	0.99	0.70	4	0.38	0.98	0.79	1
KRIPO	0.50	0.88	0.69	5	0.56	0.96	0.65	11
ProBiS	8.03	1.00	0.67	6	2.98	0.99	0.74	2
SiteEngine	875.25	0.98	0.65	7	1,063.70	0.99	0.65	10
IsoMIF	0.49	0.96	0.63	8	0.46	0.92	0.73	3
Cavbase	15.85	0.99	0.63	9	8.33	0.97	0.66	9
SiteAlign	0.85 (d1 < 0.6, d2 < 0.2)	0.99 (0.81, 0.93)	0.60 (0.77, 0.70)	10	0.80	0.93	0.72	5
FuzCav (PDB)	0.21 (0.16)	0.94 (0.70)	0.60 (0.74)	11	0.20	0.92	0.67	7
FuzCav	0.21 (0.16)	0.94 (0.70)	0.60 (0.74)	12	0.20	0.92	0.67	8
VolSite/ Shaper	0.89 (RefTversky (color) > 0.35)	0.95 (0.59)	0.58 (0.77)	13	0.99	0.99	0.56	15
SiteHopper	0.95	0.99	0.57	14	0.87	0.99	0.61	12
Shaper	0.89 (RefTversky (color) > 0.35)	0.94 (0.52)	0.57 (0.76)	15	0.98	0.99	0.54	16
Shaper (PDB)	0.89 (RefTversky (color) > 0.35)	0.94 (0.51)	0.57 (0.76)	16	0.94	0.97	0.57	14
VolSite/ Shaper (PDB)	0.89 (RefTversky (color) > 0.35)	0.96 (0.64)	0.53 (0.68)	17	0.98	0.99	0.52	17
Grim (PDB)	0.57 (0.594)	0.78 (0.96)	0.51 (0.38)	18	0.59	0.94	0.42	20
TIFP	0.23 (Tanimoto > 0.318)	0.89 (0.89)	0.49 (0.49)	19	0.17	0.83	0.59	13
Grim	0.57 (0.594)	0.90 (0.99)	0.48 (0.42)	20	0.58	0.95	0.47	18
TIFP (PDB)	0.20 (Tanimoto > 0.318)	0.89 (0.92)	0.44 (0.41)	21	0.35	0.96	0.39	21

Additionally, we used thresholds that were assigned by the developers. This was possible for FuzCav, SiteAlign (for the distances d1 and d2), Shaper and VolSite/Shaper (for the RefTversky color similarity), Grim, and TIFP (for the Tanimoto coefficient). The thresholds for SiteAlign were initially derived based on the score distributions of similar and dissimilar sites. In all other cases the F-measure[88] was applied to identify an optimum cut-off. Using the pre-defined thresholds, we observe a lower specificity for FuzCav, SiteAlign, Shaper, and VolSite/Shaper, but a higher sensitivity with respect to data set 7 when compared to our thresholds. Depending on the aim of a binding site comparison study, the optimum threshold will vary. For a drug repurposing study, a high specificity is crucial because it is interesting to retrieve only similar sites. In contrast, high sensitivity values are more suitable for off-target predictions as it is important to find as many potential off-targets as possible.

We also calculated cut-off values using data set 7 (sixth to ninth column in Table 5). The use of these thresholds leads to better specificity and sensitivity values underpinning the strong data set dependence of similarity score thresholds. Especially, the ability to detect similar binding sites increases. The high specificity, which was already high for the initial cut-offs, persists. This observation is most prominent for SiteAlign, TIFP, and IsoMIF. Importantly, not only do the sensitivity values differ for the different thresholds, but also the ranking of the methods according to sensitivity.

This finding is important for designing a binding site comparison study. Whenever searching for similar binding sites, it is not recommended to reject reasonable site matches solely based on a low score. The basic idea is to analyze the resulting list of ranked binding site similarities, visualize high scored pair alignments, and finally decide whether they are similar or not. The choice for an appropriate tool should always take into account the methods’ enrichment factors. If a similarity score cut-off for classification purposes is indispensable, the user has to take into account the aim of the comparison as well as the nature of the data set.

The cut-off values for our data sets as determined with the method of Youden[85] can be found in S12 Table, but they should never be overestimated. The variation in the thresholds calculated for the seven data sets shows that they have to be carefully chosen dependent on the anticipated outcome. Similarity scoring for binding sites is an ambitious undertaking (see benchmark studies for data set 1). The reliability of the similarity measures varies with binding site flexibility, bound ligands, and the binding site definition. The development of meaningful benchmark data sets is challenging which is due to the lack of knowledge of site-specific bioactivities and the sparseness of known protein-ligand complex structures with similar ligands. Unfortunately, meaningful cut-offs cannot be determined before having even more protein ligand complex structures and bioactivity data with binding site annotations. In line with other investigations concerning this topic[11,43,89], we argue that these factors preclude the assignment of thresholds for a binary classification of similar and dissimilar binding site pairs.

#### The discovery of unexplored site similarities

**Fig 14:**
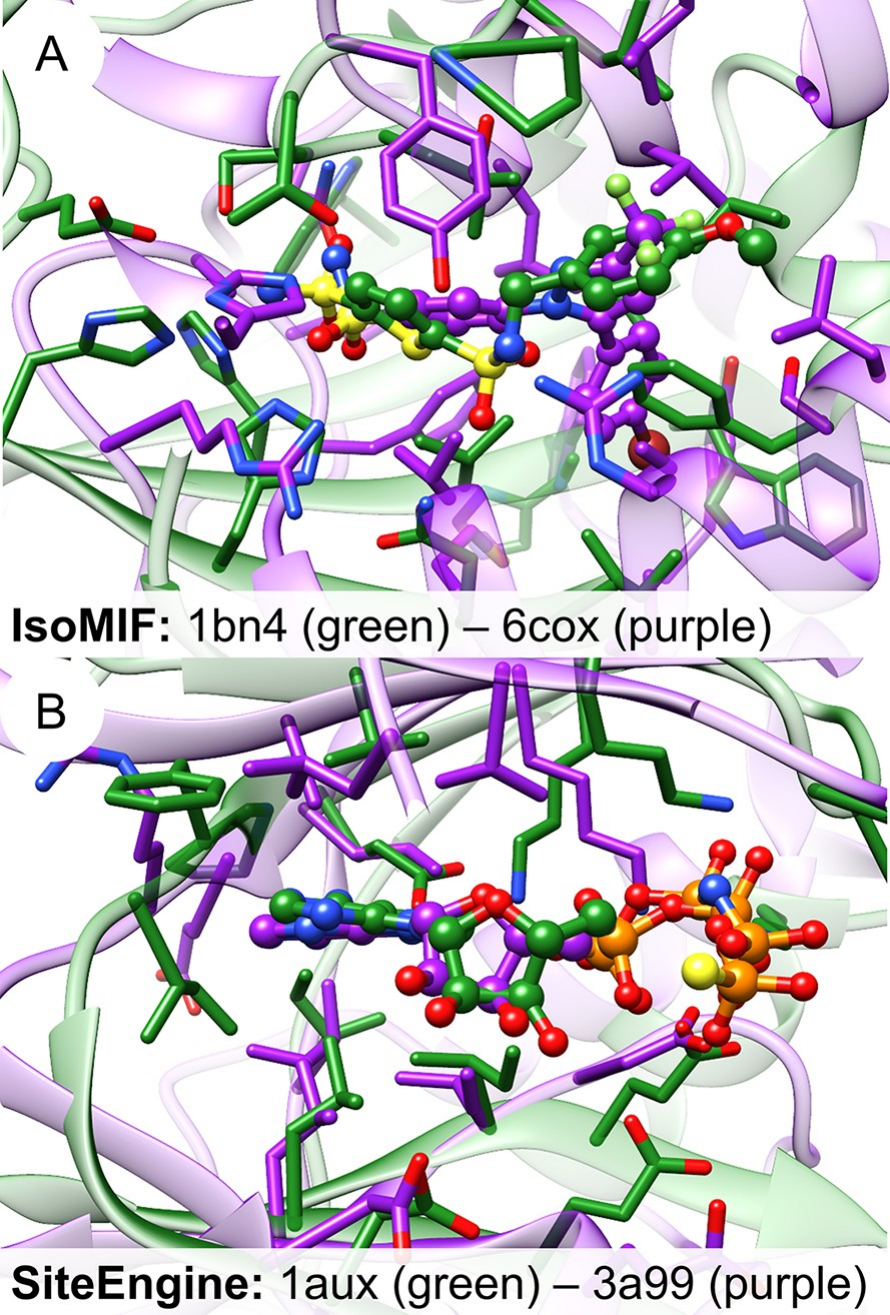
Binding site alignments for similar cavity pairs which most tools failed to identify. (A) Alignment of human carbonic anhydrase II (PDB ID 1bn4, green) and cyclooxygenase-2 (PDB ID 6cox, purple) as obtained with IsoMIF. (B) The binding sites of synapsin (PDB ID 1aux, green) and PIM-1 kinase (PDB ID 3a99, purple) as aligned by SiteEngine. All illustrations were generated using UCSF Chimera[60].

Finally, we asked the question: “What are the similarities which most of the tools miss?”. To this end, the box plots for the different methods (S3–S5 Figs) were taken as a basis for the identification of “difficult cavity pairs”. Active pairs with similarity or distance values in the range of dissimilar pairs for most methods were analyzed. The methods with the highest scores for these matches were applied to generate alignments of the pairs. Some of these are depicted in Fig 14. The general outcome of this analysis reveals the difficulties in finding similar pockets. The example in Fig 14A was first identified by the residue-based method Cavbase[35]. Intriguingly, a low Cavbase similarity score of 3.25 was assigned for this site pair in the present study. In contrast, the interaction-based method IsoMIF scored this pair highly and provided the presented site alignment. It is obvious that this match cannot be found by residue-based methods as the chemical nature of most aligned residues is different. Nevertheless, similarities were detected with respect to site interaction patterns. SiteAlign was applied for the identification of the match between synapsin and PIM-1 kinase[41]. In contrast to the previous example, SiteAlign assigns a high score to the match and it can be found within the best-scored pairs. A high ranking for this cavity pair was also observed for the surface-based method SiteEngine. The alignment in Fig 14B illustrates why it is so difficult for many methods to identify this similarity. Although the atoms of both ligands align as expected, residue correspondences are difficult to find and only sub-parts of both pockets are similar. This final analysis underlines that it is impossible to identify a “best-performing tool”. Some of the similar cavity pairs were within the best-scored hits for one, but not all tools. The results of two or more tools are often complementary. This holds especially true for binding sites sharing common interaction patterns or surface properties which are not evident on a residue-based level.

### Run time analyses and failure rates

A final aspect for choosing an appropriate tool is the computational cost required by the different methods. The interplay of cavity preparation, number of modeled binding site properties, the implementation of the comparison algorithm, and filtering steps determines the final CPU time. Therefore, the run time of binding site preparation as well as comparison per method was analyzed. Table 6 summarizes the different algorithms with respect to the run time for binding site pre-processing of all 100 structures of data set 5 and for 10,000 comparisons (all-against-all). As PocketMatch, SiteAlign, and TM-align rely on user-defined cavities, which were prepared in an automated manner using a Python script, i.e. exemplary run times are given that might change for a different site extraction method.

**Table 6:** Run time evaluation of different binding site comparison methods with respect to the data set of Kahraman *et al*.[63]. The numbers in brackets highlight the number of successfully prepared pockets or the number of comparisons, respectively. The average run times are colored by a gradient ranging from green (minimum run time) to yellow to red (maximum run time).

method	data basis for comparison	run time preparation [s] (number of structures)	run time comparison [s]	total run time [s]	average run time per comparison [s]
PocketMatch[24]	distance lists	28.97*	0.28	29.25	0.000028
KRIPO[56]	fingerprint	446.50	0.92	447.42	0.000092
RAPMAD[31]	histogram	71.42 (100)	2.36 (8,281)	73.78	0.000285
FuzCav[36]	fingerprint	399.88 (96)	5.59 (9,216)	405.47	0.000607
FuzCav(PDB)[36]	fingerprint	236.73 (96)	5.64 (9,216)	242.37	0.000612
TM-align[27]	matrix	25.72*	65.96	91.68	0.006596
Shaper(PDB)[23]	3D points (grid)	181.16 (96)	364.42 (9,216)	545.58	0.039542
Shaper[23]	3D points (grid)	384.21 (96)	367.21 (9,216)	751.42	0.039845
VolSite/Shaper[23]	3D points (grid)	537.00 (76)	248.77 (5,776)	785.77	0.043070
ProBiS[48]	graph	6.95	479.32	486.27	0.047932
VolSite/Shaper(PDB)[23]	3D points (grid)	259.54 (57)	162.26 (3,249)	421.80	0.049942
TIFP[19]	fingerprint	228.30 (77)	550.88 (5,929)	779.18	0.092913
TIFP(PDB)[19]	fingerprint	194.36 (47)	205.56 (2,209)	399.92	0.093056
Grim(PDB)[19]	graph	169.33 (96)	1,714.49 (9,216)	1,883.82	0.186034
Grim[19]	graph	220.17 (95)	2,104.99 (9,025)	2,325.16	0.233240
IsoMIF[22]	graph	752.83	2,561.44	3,314.27	0.256144
SiteHopper[25]	3D points	154.01	3,828.61	3,982.62	0.382861
Cavbase[20,21]	graph	67.89 (100)	21,823.71 (8,281)	21,891.60	2.635396
SMAP[43]	graph	1.69	42,346.74	42,348.43	4.234674
SiteEngine[51]	3D points	328.81	81,193.54	81,522.35	8.119354
SiteAlign[18]	fingerprint	28.97*	286,326.41	286,355.38	28.632641

_* exemplary run times for methods that demand a pre-processing by the user_

Interestingly, the tools were not as robust as expected and failed at different steps of preparation and comparison. The failure rates of all methods for the analyzed data sets are given in S10 Fig. The most interesting finding was the importance of PDB to MOL2 conversion to decrease the failure rates of Grim and TIFP. Both methods should be preferentially applied with MOL2 files of the protein-ligand complexes.

The calculation of the average run times per comparison takes the number of omitted comparisons into consideration. For Cavbase and RAPMAD, all structures could be processed, but the ligand-defined binding site was not identified for nine structures. Therefore, the comparison run time is given for 8,281 comparisons. For FuzCav and Shaper, only 96 out of 100 binding sites could be prepared. A pre-processing of the prepared pockets (with the PDB file parser pdbconv of IChem) with VolSite for a subsequent Shaper comparison (VolSite/Shaper) led to only 57 pockets derived from PDB files. In contrast, 76 cavities were extracted with MOL2 files as input for pdbconv. TIFP calculations led to 47 fingerprints for PDB files and 77 for MOL2 files. In Table 6, the numbers in brackets summarize the numbers of prepared structures and comparisons for each tool.

A clear correlation between the comparison method used and the run time could not be observed. Nevertheless, it is possible to differentiate between very fast methods (several microseconds per comparison), moderately fast methods (several milliseconds per comparison), and comparably slow methods (several seconds per comparison). Depending on the desired outcome and the size of the data set, the computational cost might be a limiting factor and the use of some methods becomes infeasible. For a comparison of a single binding site of interest against all known pockets as stored in the sc-PDB[65] (9,283 entries) on a single CPU, three days will be necessary using SiteAlign while PocketMatch will perform these comparisons within 0.28 seconds. For an all-against-all comparison, RAPMAD is approximately 9,200 times faster than the graph-based method Cavbase. In contrast, TIFP is only twice as fast as the graph-based method Grim. Such minor differences suggest the use of the better-performing method. The run time also depends on the type of comparisons. Pairwise comparisons were performed for Grim, IsoMIF, VolSite/Shaper, SiteAlign, SMAP, TIFP, and TM-align, i.e. the tool was invoked for each comparison separately. This might become the time-limiting step for some methods. Cavbase, FuzCav, PocketMatch, ProBiS, RAPMAD, SiteEngine, and SiteHopper allow the comparison of one query against a list of targets in one run. This causes a speedup as the tool has to be invoked only once. For ProBiS, the conversion of PDB files to a surface file format is necessary. Thus the cavity preparation time reflects this preliminary step. Additionally, KRIPO and RAPMAD enable even faster comparisons by providing SQLite databases of the modeled sites and the automated calculation of similarity matrices. These factors illustrate the difficulty in analyzing and comparing run times. In this analysis, we chose the fastest option available for each tool. The computational cost will therefore differ depending on the tool’s implementation and the type of approach employed (comparing distinct binding site pairs, query-based analyses, generation of distance matrices, etc.).

Finally, the nature of the data set has to be taken into consideration. Changing the binding site size might have a huge impact on the run time per comparison. While the run times of fingerprint-based methods are often not affected by this factor, graph-based methods might become significantly slower with an increasing graph size. Cavbase, for example, fails to compare very large binding sites. ProBiS uses pre-filtering steps to speed up comparisons.

## Discussion

Plenty of factors influence the decision for a suitable binding site comparison tool. S13 Table summarizes the tools’ characteristics with respect to the most important criteria and provides a detailed assessment of the tools’ performance. First of all, the necessary pre-processing of structures might have an impact on the choice. The preparation of large data sets, e.g. for the prediction of potential off-targets using all known binding sites, requires tools that enable an automated and flawless binding site processing and annotation. We divided this criterion into two parts. Firstly, it is important that binding sites can be prepared in an automated fashion. For example, the KRIPO developers generated scripts to automatically process the binding sites of interest. The same holds true for FuzCav, Grim, TIFP, Shaper, and VolSite/Shaper (although for some PDB structures ligand MOL2 templates have to be provided). Cavbase and RAPMAD require XML-formatted cavity descriptions as input which can be generated with the help of the CSD Python API[90] in an automated fashion. Secondly, the inclusion of all available binding sites might be crucial (S10 Fig). The prediction of potential off-targets and identification of novel binding sites require comprehensive cavity data sets. It is also vital that the chosen tools are reasonably fast with respect to such applications (run time).

Sometimes, it is of interest to investigate the similarity between predicted binding sites and already known ones, e.g. for the identification of druggable binding sites of novel targets. Many interaction-based methods will not be able to compare predicted pockets (Grim, KRIPO, TIFP). SiteEngine will also fail as the tool relies on protein- and ligand-surface construction to compare the resulting sites’ surfaces. Furthermore, SMAP does not provide the possibility to process detected binding sites. The only possibility is the generation of putative ligand binding modes via docking or pharmacophore searches to obtain structures with bound ligands for these methods. The tools Cavbase, FuzCav, RAPMAD, Shaper, and IsoMIF come along with implemented binding site identification approaches. Additionally, PocketMatch, ProBiS, SiteAlign, and TM-align process user-defined binding sites based on residues. Therefore, it is possible to apply them to externally predicted binding sites. SiteHopper was shown to compare predicted pockets as long as fpocket[91]-derived cavities are utilized as a pseudo-ligand[53].

Based on the analyzed data sets we can also draw conclusions with respect to a suitable input for binding site comparison. Tools that showed a poor performance for data set 1 should not be used if only a small number of protein-ligand complex structures are known. These tools are very sensitive toward the nature of the ligand. The problem may possibly be circumvented by using docking-derived poses of ligands in the binding site of interest. Some methods’ scoring schemes suffer from a small similarity score difference window for similar and dissimilar binding sites.

In many cases, it is advisable to use all available crystal structures or multiple NMR models to retrieve promising results. Although the accuracy of NMR structures is poorly validated[92], they seem to be an acceptable choice for different structural biology and computational modeling challenges[93,94]. The G-factors calculated with PROCHECK-NMR (Table 2) indicate a lower quality as compared to the X-ray data sets. Nevertheless, the average of -0.28 point toward usual dihedral angles and main-chain covalent forces. Several tools were developed to facilitate the selection of a diverse set of representative protein structures[95,96]. In the absence of experimental structural ensembles, the use of MD-derived binding site conformations as input for a comparison might be beneficial.

With respect to the decoy data sets, there are only a few tools that are able to distinguish between similar pockets with minor dissimilarities and highly dissimilar sites. SMAP, SiteHopper, and KRIPO are best suited to convincingly penalize differences between protein structures and their artificially created decoy variants with different numbers of substitutions. Other tools failed to score dissimilarities appropriately. This can be partially attributed to the fact that the binding site’s shape was retained in data set 3; yet, the introduction of major shape differences (data set 4) did not cause substantial improvements. Tools which do not reliably score minor differences in structures might provide better results if they are applied to elucidate similarities between unrelated cavities. In such cases, residue changes and geometric deviations have to be tolerated to identify such partial similarities within the best-ranked binding site pairs.

The evaluation of the tools with regard to the data set of Barelier *et al*.[64] shows that a final visual inspection is unavoidable if the user is interested in a drug repurposing strategy or the establishment of polypharmacology. Therefore, we have to differentiate between the methods depending on the possibility to visually analyze the resulting binding site alignment.

The data set of similar binding site pairs extracted from lifelike analyses gives some insight into the basic applicability of the tools for different scenarios. The observation that all tools showed a very high early enrichment underlines the fact that all tools perform well for relevant examples. The individual strengths and weaknesses of all tools finally level out to considerably high early enrichment of similar binding site pairs for all methods. Whenever possible, it is advisable to use more than one tool as they might complement one another.

The comparison methods Grim and TIFP are characterized by a comparably low applicability toward the identification of binding site similarities. The original application domain both tools were designed for was the rescoring of docking poses and pose selection, e.g. in a structure-based virtual screening campaign. For these approaches they perform well as shown in the original publication[19] and quite recently for the D3R grand challenge 2015[97]. We could not use the pre-processed structures as stored in the sc-PDB[65] as our data sets make use of all available PDB structures. Restricting the binding site comparison with TIFP and Grim to sc-PDB derived structures might provide a different perspective for both tools as their performance depends highly on the pre-processing of the binding sites. Both tools assign interaction patterns depending on the nature of the ligand which impairs their success for some of our chosen data sets. Nevertheless, their performance on the data set of lifelike examples was good.

Finally, the application of score thresholds to discriminate between similar and dissimilar binding sites can be discussed. Similarity scoring for binding sites is challenging. Often, score cut-off values have to be optimized for specific studies[11]. Our investigations also show the strong context dependency of such thresholds (S12 Table). If a clear classification of similar and dissimilar pairs is necessary, the appropriate cut-off has to be evaluated with a data set that reflects the main purpose of the analysis. Score thresholds for the decoy data sets (data set 3 and 4) should be taken into account if binding sites with minor dissimilarities are compared. In contrast to the elucidation of local binding site similarities or similarities of pockets of unrelated proteins, the less restrictive cut-offs determined for the data set of Kahraman and colleagues[63] or data set 7 are more appropriate. The methods SMAP and ProBiS offer additional statistical measures to estimate the significance of a binding site match. Nevertheless, we want to emphasize that the application of thresholds often means disregarding potentially interesting and yet unexplored binding site similarities.

**Fig 15:**
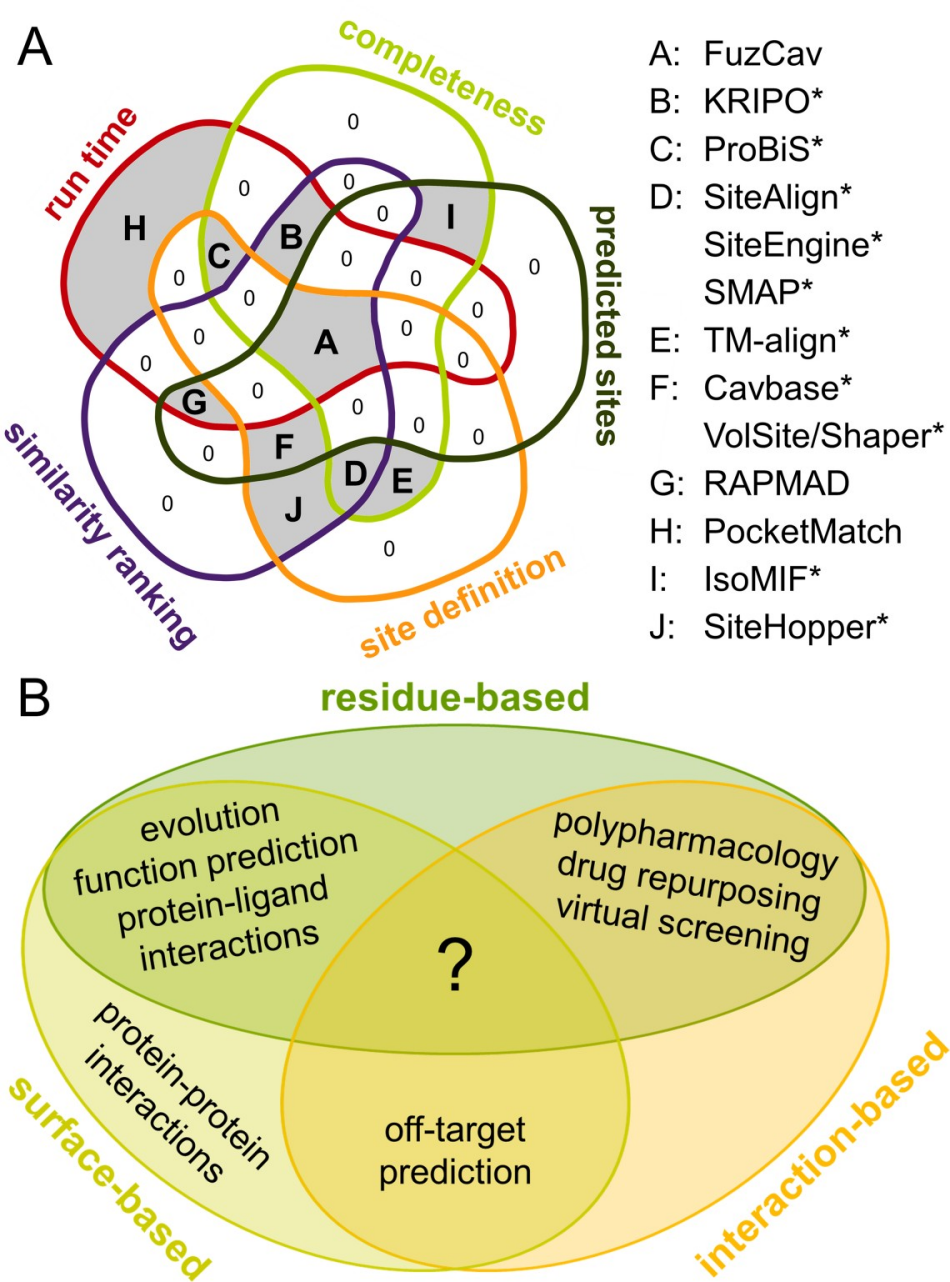
Guiding the choice of appropriate binding site comparison tools. (A) Venn diagram illustrating differences in the strengths of the comparison methods based on a subset of quality criteria. An asterisk marks methods which provide a binding site alignment for a visualisation of site similarities. (B) Venn diagram of successful applications of the evaluated residue-, surface-, and interaction-based tools in different research scenarios. Both diagrams were generated using DrawVenn[100].

The Venn diagram in Fig 15A summarizes six potentially meaningful categories which influence the decision on a useful tool. Depending on the problem to be addressed, other criteria out of a multitude as given in S13 Table might also facilitate the choice. We strongly recommend this comprehensive table for further details. Nevertheless, we wanted to give an example for guiding the way to the most appropriate tool according to the criteria selected. FuzCav seems to fulfil all criteria in the Venn diagram and can be safely applied for evolutionary analyses. However, the binding site matches cannot be visualized. Hence, the method is not suited for query-based drug repurposing projects which require a detailed examination of the identified similarities. The use of KRIPO enables the generation of site alignments, but the method is not applicable for predicted sites and consequently not suited for function prediction. ProBiS failed to rank the similarity according to the number of residue substitutions and will probably fail to accurately score minor cavity dissimilarities. As this is decisive in the analysis of evolutionary binding site relationships, such scenarios should be analyzed with SiteAlign, SiteEngine, or SMAP. A potential user can thereby iteratively exclude tools to arrive at a final choice.

Fig 15B puts all results into context and can be used to discuss possible application areas for the binding site analysis tools. The applicability suggestions for the different types of tools are based on previous studies illustrating different uses in drug discovery[5]. However, the diagram also hints at possible fields of applications yet unexploited, for example, the use of interaction-based methods for selectivity prediction or surface-based methods for target elucidation. Of course, the general aim of a study, the basis of data, and the number of comparisons necessary will influence the choice of a method. The embedding of a tool in an elaborate *in silico* workflow might serve to diminish certain weaknesses. Methods never applied to date for a certain field of research might provide useful results within specialized projects. Some obvious new applications are the use of interaction-based methods for the analysis of protein-protein and protein-ligand interactions or drug repurposing approaches exploiting the outcome of surface-based site comparisons. The prediction of off-targets with comparably fast residue-based approaches is another unexplored application. Furthermore, some tools have never been applied in independent studies. Several projects which made use of Grim[97], RAPMAD[98], and SiteHopper[99] demonstrate their unique capabilities. Their strengths as highlighted herein might encourage researchers to actively use these tools.

Although it is not possible to identify any single tool which fits all needs and performs well for all data sets, we can provide some guidance regarding different aspects of binding site comparison. The impact of the ease and completeness of binding site pre-processing steps increases with an increasing number of proteins used for comparison. While both factors are crucial for elaborate projects, e.g. off-target prediction or the identification of novel binding sites, their importance decreases for relatively small data sets used in selectivity profiling or polypharmacology prediction. An application to predicted binding sites is relevant for off-target prediction, drug repurposing, or the identification of potential targets. For polypharmacology elucidation, one generally refers to known druggable binding sites. The analysis of similar binding sites to elucidate minor structural differences is highly influenced by the binding site’s definition and flexibility, as is the ranking with respect to binding site properties. In contrast, the elucidation of similarities between the cavities of unrelated proteins does not necessarily depend on accurate scoring. MD simulations or the use of NMR ensembles might help to circumvent a potential failure of binding site comparison due to insufficient consideration of protein flexibility. It is necessary to unravel the similarity between functionally unrelated binding sites which bind similar ligands when understanding polypharmacology. Visualization of identified similarities, which is essential when dealing with non-obvious similarities, is not necessarily crucial for selectivity profiling or analyzing evolutionary relationships. These criteria should become the focus of future benchmark analyses of other promising site comparison approaches as such analyses can guide the rational choice of a method.

Ultimately, the choice of a comparison method depends on the focus of the study. These investigations can help to ease the choice of a suitable tool, though restricted to a limited subset of available approaches. The publication of the generated data sets and the benchmark results can assist in the assessment of tools and the establishment of reliable workflows that consider individual strengths and weaknesses. We hope that the assembly of benchmark sets (ProSPECCTs) and the conclusions drawn from the evaluation encourage researchers to objectively assess the advantages and drawbacks of individual approaches. Finally, this guide could facilitate the final choice of a suitable method and enable researchers to derive an advantage from these–as far as our experience goes—widely underemployed binding site comparison approaches.

## Methods

### Data set preparation

The structures of all data sets were prepared in the same way to ensure an equivalent basis for all binding site comparison tools. First, modified residues were identified and the respective HETATM record names in the PDB files were changed to ATOM. This modification did not affect the tools’ performance, but was essential for the site processing with SiteEngine which detects ligands based on the HETATM record names. The binding site’s defining ligand was identified and renamed to LIG for further processing steps. Other HETATM entries were deleted to ensure the exclusion of buffer ions, cofactors, and prosthetic groups. The final steps were realized with the help of the pdbcur tool of the CCP4[101] software package (version 6.5). Alternative locations with the highest occupancy were retained, while for locations with identical occupancy values the first one was retained. Finally, ANISOU entries were removed.

### Data set characterization

The resolution and R-factors (R work) for all structures were downloaded as a report from the PDB[1]. The mean values, standard deviations, minima, and maxima of these parameters were calculated for all data sets including X-ray structures. G-factors were calculated using PROCHECK[66] and PROCHECK-NMR[67]. These values measure the degree of unusual dihedral angles and covalent forces of the main chain. G-factors below -0.5 hint at unusual structure properties. For the groups of structures with identical sequences and the NMR ensembles, all residues were renumbered according to the sequence alignment calculated with default settings in MOE2013[102]. Binding site residues were assigned based on a 5 Å radius of all ligand atoms. Subsequently, the Cα atoms and all atoms of the binding site-defining residues were aligned using the “match” command of UCSF Chimera[60]. The mean RMSD values, standard deviations, minima and maxima of all pairwise comparison were calculated to characterize the binding site flexibility. For the data set of identical structures, Tanimoto coefficients based on the ECFP4 fingerprints were calculated in a pairwise manner for all groups using KNIME[103].

Binding site descriptors were calculated using DoGSite. The binding site ligand was chosen as the reference ligand and the pocket was defined by the ligand. Apart from these changes, default settings were used. The resulting pocket descriptors were analyzed for each NMR ensemble of data set 2, the sequence-culled set of the sc-PDB and the structures of the data set of Barelier and co-workers[64].

#### Structures with identical sequences

The sequences of all single chain entries of the sc-PDB[65] were sequence culled using the PISCES[104] server (with a sequence identity threshold of 25%). The single chain sc-PDB entry sequences of the culled set were compared to those of all single chain proteins as stored in the PDB using USEARCH[105]. Structures whose sequences were identical to those of at least nine others were retained. This resulted in a data set of 13 groups of diverse structures. The PDB structures within each group were aligned and the ligand-occupied binding sites were compared to ensure that all ligands were located at the same site. This visual inspection led to the exclusion of one group represented by the sc-PDB entry with the PDB ID 4l8u (chain A) as the ligand-defined binding sites differed for the structures with 100% sequence identity. A complete list of data set 1 can be found in S9 Table. An all-against-all comparison was performed to assess the performance of each tool. Site pairs of proteins with identical sequences were regarded as positives while other matches were defined as negatives.

Additionally, a reduced set of structures (data set 1.2) was generated which exclusively contains structures with identical sequences and similar ligands. To this end, we calculated ECFP4 fingerprints for all ligands in KNIME and iteratively excluded binding sites with ligands that show a Tanimoto coefficient below 0.6 to any other ligand in one group.

#### NMR structures

A sequence culling using PISCES[104] was performed for all in the sc-PDB[65] listed NMR chains with more than one conformer. A sequence similarity threshold of 25% was set. As a result, 17 diverse PDB entries containing different numbers of models were obtained (data set 2). An all-against-all comparison was performed and matches between models of the same structure were denoted positive. Although the NMR structures with the PDB IDs 1diu (chain A) and 1yho (chain A) are homologous enzymes from two different organisms (dihydrofolate reductase, see S10 Table), the binding sites show major dissimilarities. Therefore, binding site similarities between models of one enzyme should be ranked higher than similarities between models of both enzymes. Matches between conformers of both structures were defined as false positives.

#### Decoy structures

One representative of each group of structures with identical sequences was chosen for the introduction of one, two, three, four, and five binding site residue substitutions. Randomly chosen solvent accessible binding site residues (random module in Python) were substituted by physicochemically different residues by means of the SwissSidechain[106] plugin of Chimera[60] (decoy set 1). This plugin offers the possibility of choosing energetically favorable side chain conformations of the residues introduced by using the Dunbrack backbone-dependent rotamer library[107]. Substitutions were designed in a way that the introduced residues were isosteric with those replaced, but showed different physicochemical properties. The ligand was retained in the binding site of interest. For the second data set of decoy structures (decoy set 2), the surface exposed residues were randomly chosen for substitution and replaced by residues which differ in the number of hetero and carbon atoms by at least three from the original residue. Table 7 summarizes the substitution residues for the twenty naturally occurring amino acids.

**Table 7:** Overview of substitutions to generate a data set of rationally modified decoy structures.

**original residue**	Ala	Cys	Asp	Glu	Phe	Gly	His	Ile	Lys	Leu
substitution (decoy set 1)	Gly	Ala	Leu	Arg	His	Ala	Phe	Thr	Glu	Asp
substitution (decoy set 2)	Ile	Lys	Ala	Cys	Leu	Val	Ser	Ala	Ser	Ala
**original residue**	Met	Asn	Pro	Gln	Arg	Ser	Thr	Val	Trp	Tyr
substitution (decoy set 1)	Gln	Leu	Ala	Met	Glu	Ala	Val	Thr	Phe	Phe
substitution (decoy set 2)	Cys	Ala	Gly	Ala	Val	Lys	Gly	Gly	Phe	Ile

The data sets were applied in two different ways that allowed for a more sophisticated analysis. First, we analyzed whether the pairs of native structures were assigned higher scores than the variants with one, two, three, four, and five substitutions. Next, the analysis was repeated by omitting all variants with less than five mutations. Furthermore, the correlation between score and number of mutations was investigated by means of the Spearman’s rank correlation coefficient (see below for details).

#### Kahraman data set

The Kahraman data set[63] consists of 100 protein structures that are listed with their corresponding ligands in S7 Table. The data set includes inorganic phosphate ions as ligands. We evaluated the performance of different tools for an all-against-all comparison of the data set in the absence (data set 5) and in the presence (data set 5.2) of the 20 phosphate binding sites.

#### Barelier data set

The Barelier data set (data set 6) was designed based on the publication of Barelier and co-workers[64] who analyzed the relationships between binding sites of unrelated proteins binding to identical (or in some cases similar) ligands. All structures were prepared retaining all protein chains. An overview of the structures and respective ligands can be found in S8 Table. The structure with the PDB ID 4odj consists of a single chain in the PDB entry. Nevertheless, the native protein can be regarded as a dimer that binds the ligand at its interface. Therefore, we decided to use the dimer based on a symmetry copy to obtain a complete binding site. While binding site pairs of class A were used as positives, the remaining pairs were used as negatives. Finally, we included cofactors in the pairwise comparison whenever possible (data set 6.2).

#### Data set of successful applications

The final and most realistic data set consists of binding site pairs which were previously characterized as being similar in published literature. The similarities as summarized elsewhere[5] were manually analyzed and pairs of similar binding sites were extracted. Altogether, we used 49 query structures (S11 Table). We created a sequence-culled data set of the sc-PDB[65] single chain structures with a sequence identity threshold of 25% (1,103 structures) with the help of the PISCES[104] server. This data set was combined with our query structures. Finally, we assessed the retrieval of 115 cavity pairs with known binding site similarity when comparing the query structures to the complete data set.

#### Data set redundancy

The redundancy of the benchmark entries was evaluated using the DrawVenn[100] web server. The PDB IDs which were found in more than one data set are summarized in Table 8.

**Table 8:** Redundancy of the benchmark entries. The PDB IDs of redundant entries are given with the corresponding data sets and the number of structures in both sets.

data sets	number of structures	PDB IDs
data set 1 / data set 2	326 / 17	1yho
data set 1 / data set 6	326 / 115	1z9y
data set 1 / data set 7	326 / 1,151	4bfz, 3rm2, 4fpt, 3f17, 1odm, 2qxw, 1kmv, 3u5l, 4buu, 4ca7, 3u9w, 3t10
data set 2 / data set 7	17 / 1,151	1t84, 1diu, 1cz2, 2k5t, 1j5i, 1tvc
data set 5 / data set 7	100 / 1,151	1tox, 1b8o, 1t2d, 1kht, 1jq5, 1kvk, 1ct9, 1cqx, 1a49
data set 6 / data set 7	115 / 1,151	1eyq, 2oyf

### Binding site comparison

Unless stated differently, default parameters of the analyzed tools were used. The exception that holds true for all methods is that the scoring measure used for ranking was selected based on the early enrichment for the data sets of structures with identical sequences, NMR, and decoy structures with descending priority (Table 9). Regardless, all scores were calculated for further evaluation. Additional information for the different scoring schemes used is given in the SI (S1 Text).

**Table 9:** Summary of the scoring schemes provided by different comparison tools. An asterisk marks the scheme that was used for the evaluation of tools that offer more than one similarity or distance measure.

method	scoring schemes
Cavbase[20,21]	similarity score
FuzCav[36]	similarity score
Grim[19]	Grscore
IsoMIF (based on IsoCleftFinder[54])[22]	tani*, taniM, taniMW, taniNormNodes, RMSD
KRIPO[56]	similarity score
PocketMatch[24]	PMScore_max_*, PMScore_min_
ProBiS[48]	z-score, SVA, RMSD, Alignment Score*
RAPMAD[31]	distance
VolSite/Shaper[23]	Tanimoto (color, fit, combo*), RefTversky (color, fit, combo), FitTversky (color, fit, combo)
SiteAlign[18]	distances d1, d2, d3*, d4
SiteEngine[51]	LowResolutionScore, OverallSurfaceScore, DistanceScore, CurvatureScore*, TotalScore
SiteHopper[25]	Tanimoto (color, shape), PatchScore*
SMAP (based on SOIPPA[42])[43]	Tanimoto, RawScore*
TIFP[19]	Tanimoto, Hamming, Ref Tversky, Fit Tversky, Dice, Soergel*
TM-align[27]	TM-score

Most binding site comparison tools applied in this study have some major limitations that have to be taken into account when applying them to different types of data sets. They will be discussed in the following.

PocketMatch (version 2.0), SiteAlign (version 4.0), and TM-align (version 20170708) rely on pre-processed ligand binding sites. The respective binding sites were extracted by means of a Python script which creates an output PDB file that contains all protein atoms within a given radius of the ligand atoms. This excised binding site was used for the comparison. A 5 Å radius was applied for the creation of input cavities for PocketMatch and SiteAlign. Residues within 10 Å of the ligand were used as the binding site definition for TM-align to guarantee a sufficient number of residues to yield a reliable alignment.

The programs Cavbase and RAPMAD require XML-formatted pockets as input for binding site comparison. The extraction of the pockets was achieved using the CSD Python API 1.3[90] from the CCDC. Residues with missing backbone atoms which were not part of the binding site were excluded to obtain pre-processed cavities as these residues were not properly processed. The cavity extraction is based on pockets detected by LIGSITE[76]. Thus, the binding site of interest could not be found for some PDB entries. We used only cavities including ligand atoms whereas other detected pockets were excluded from the analysis. If more than one pocket for the ligand of interest was identified, the pocket with the largest cavity volume was used. Some very large cavities could not be processed using Cavbase. The use of RAPMAD is restricted to similarity scoring of binding sites as it does not generate a binding site alignment.

FuzCav comparisons can be performed for binding sites extracted with the pdbconv tool of IChem, i.e. the complete site or residues within 4, 6, 8, 10, or 12 Å of the ligand can be used for the comparison. A 6Å radius was used in our analyses.

IsoMIF is a molecular interaction-based method that generally relies on bound ligands. A cut-off radius can be used to restrict the binding site dimensions depending on the ligand. The use of the additional tool GetCleft[108] allows for the inclusion of predicted cavities.

KRIPO fingerprint databases can be individually created by users for their own data sets. A ligand database is provided for all PDB entries. For proprietary or modified structures, a ligand database has to be generated. Fingerprints can be prepared for ligand fragments as well as for complete ligands with the command line tool “kripo”. Subsequently, the KRIPO DB package (“kripodb”) was used to perform all-against-all comparisons for the derived fingerprint databases for complete ligands. The final similarity matrix did not include similarities between identical structures. The identity scores were automatically set to 1.

PocketMatch does not take modified residues into account. The tool is suitable for similarity ranking but the alignments are not available as output. The PDB convention of 80 characters per line has to be fulfilled for all input pockets.

Binding sites are defined by the ligand structures for ProBiS comparisons. A distance threshold can be applied to modify the cavity definition. By default, ProBiS does not provide scores for all binding site pairs of interest. Cliques with poor scores and/or low z-scores are deleted. The “noprune” and “z-score” options offer the output of insignificant matches together with the significant ones.

The use of Shaper relies on cavity definitions by VolSite. The program predicts the druggability of binding sites and excludes those that are denoted non-druggable. Not all cavities can therefore be processed with Shaper. We additionally applied Shaper to binding sites extracted by the pdbconv tool alone to allow for a more complete processing of binding sites although this is not recommended by the developers. The complete detected cavity as well as cavities defined within a 4, 6, 8, 10, or 12 Å radius of the ligand can be used. According to the recommendations, we used a 6 Å radius.

Modified residues cannot be processed by SiteAlign. A binding site definition based on residue names and numbers is necessary. Insertion numbers are not supported requiring a preliminary renumbering of residues with insertion codes in the PDB files. The comparison of one query against a list of targets frequently fails with “Segmentation Error”. It is unavoidable to do comparisons of one query against one target each to avoid high failure rates.

Alternate atom locations are not supported by SiteEngine. A single conformation has to be retained in the PDB file. Moreover, the HETATM entries for modified residues have to be changed to ATOM entries for correct surface construction. The tool is highly sensitive toward PDB files with more than 80 characters per line. Some large protein structures were not properly processed and had to be excluded. SiteEngine comparisons depend on ligand-defined cavities and its use is restricted to protein binding sites with bound ligands. The distance cut-off can be manually adjusted to define the binding site.

SiteHopper initially creates binding site patches. This step failed for some protein-ligand complexes. Furthermore, the tool was not able to process residues with missing backbone atoms. These residues were therefore excluded for the comparisons after ensuring that they are not part of the binding site of interest. SiteHopper relies on ligand defined binding sites, but the cut-off radius can be adjusted. For small ligands, an additional flag had to be set during protein-ligand splitting (-min_atoms). This flag was set to 0 to process phosphate binding sites and to 1 for the remaining data sets.

SMAP (version 2.0) comparisons worked for nearly all structures after providing the PDB files in an appropriate manner. In some cases, the tool was highly sensitive with respect to the provided structures (including REMARK lines). The binding site definition can be modified using a distance threshold.

The tools TIFP and Grim (as tools of IChem version 5.2.6) are based on interaction fingerprints. Only ligand-occupied cavities can therefore be compared. The tool pdbconv of the IChem toolbox must be applied to extract proper binding sites and interaction fingerprints. Problems also arose when the ligand of interest giben in the PDB format was not in the predefined template files. Input structure preparation therefore included a PDB to MOL2 conversion with the CSD Python API 1.3[90] to obtain more reliable fingerprints. Consequently, we applied two analyses (with PDB and MOL2 files). The same holds true for FuzCav, Shaper, and VolSite/Shaper.

The algorithm underlying TM-align relies on a given residue sequence alignment. Binding site atoms within 10 Å of the bound ligand were used to ensure a meaningful comparison. As the tool relies on an initial sequence alignment of cavity residues, a small number of residues prevents the alignment of the residues of interest.

### Performance assessment

The ranking lists of the binding site comparison tools were used to investigate their performance with respect to AUC and EF.

The ROC curves were plotted with the help of the KNIME[103] ROC Curve node to analyze sensitivity (true positive rate, Eq 1) and specificity (true negative rate, Eq 2) of the tools.

$$sensitivity=\frac{TP}{P}=\frac{TP}{TP+FN}$$ (1)

$$specificity=\frac{TN}{N}=\frac{TN}{TN+FP}$$ (2)

The AUC values for the resulting ROC curves were also calculated using KNIME.

Statistical analyses of the AUC differences were performed according to DeLong *et al*.[109] as implemented in the R[110] package pROC[111].

The EF describes the enrichment of similar (active) binding site pairs opposed to the number of similar pairs identified in a random screening (Eq 3).

$$EF_{x\%}=\frac{N{\left(actives\right)}_{x\%}/N_{x\%}}{N{\left(actives\right)}_{100\%}/N_{100\%}}$$ (3)

The EF for x% of the screened data set is calculated based on the number of true actives at this percentage (N(actives)_x%_) and the number of all pairs at this percentage (N_x%_) in the list of pairs with ranked similarity/distance score, the number of true active pairs (N(actives)_100%_), and the number of all pairs in the complete data set (N_100%_). For tools with more than one scoring scheme, we analyzed the early EFs. The score that led to the highest early enrichment for the identical structures was taken into account. If no distinction was possible, the results for the data set of NMR structures were used in a similar manner. Finally, the data set of decoy structures was taken into account. The applied scores can be found in Table 9.

Notched box plots of scores for active and inactive pairs in the data set of structures with identical sequences were generated using the software package R[110]. The Welch’s two-sample t-test[112] for the similar and dissimilar pair score distributions was performed using the software package R[110]

The Spearman’s rank correlation coefficient (Spearman’s Rho, r_S_) was calculated for the decoy binding site data sets according to Eq 4.

$$r_{S}=\frac{cov\left(rank_{X},rank_{Y}\right)}{\sigma_{rank_{X}}\sigma_{rank_{Y}}}$$ (4)

The raw scores (X_i_) and the number of mutations (Y_i_) are converted to ranks (rank X, rank Y). The covariance of the rank variables divided by the product of the standard deviations of both rank variables gives the final correlation coefficient. In this study, the correlation between binding site similarity score and number of binding site mutations was calculated. The general expectation is that the higher the number of binding site mutations the lower the score. Thus, if the Spearman’s Rho equals -1, it denotes a perfect correlation.

Optimum similarity score cut-off values for each method were determined using the R[110] package pROC[111]. Youden’s J statistic[85] was applied without weights to derive a score threshold that optimizes both sensitivity and specificity for the corresponding data set. The thresholds based on data set 1 and data set 7 were used to determine the methods’ sensitivity and specificity for data set 7.

### Run time evaluation

All-against-all comparisons with the structures of data set 5 were performed on an Intel Xeon workstation (E5-2690 with 2.90GHz and 32 GB RAM) in a serial manner (single core). The time for the pocket preparation was disregarded for SiteAlign, PocketMatch, and TM-align as this was realized separately. It has to be considered that for the remaining binding site comparison software, this is achieved within the given run time for preparation. For ProBiS, preparation and comparison are performed on the fly. Additionally, the time for summarizing the results is not included. The run time was assessed with the Linux “time” command (user time).

## Supporting information

S1 Text Brief description of the analyzed binding site comparison methods. (PDF)

S1 Table Summary of binding site comparison tools and the evaluation data sets used to indicate their applicability and strengths. (PDF)

S2 Table Statistics of binding site RMSD values for the groups of structures with identical sequences. (PDF)

S3 Table Statistics of the Tanimoto coefficients for all ligand pairs within the reduced data set 1 which results from the exclusion of highly dissimilar ligands within each group. (PDF)

S4 Table Results of the Welch’s two-sample t-test for the active and inactive pairs of data set 1. (PDF)

S5 Table Statistics of binding site RMSD values for the NMR ensembles. (PDF)

S6 Table Binding site descriptors for all NMR ensembles of data set 2 as calculated with DoGSite[73]. Mean, standard deviation, minimum, and maximum are given for four descriptors of the binding sites as defined by the ligand.
(PDF)

S7 Table Overview of the data set of Kahraman and co-workers[63]. (PDF)

S8 Table Overview of the data set of Barelier *et al*.[64] and the RMSD values obtained by a least-squares fitting of corresponding ligand atoms of both complexes. (PDF)

S9 Table Data set of structures with identical sequences (data set 1). Structures which are also in the reduced data set 1.2 are highlighted in bold characters.
(PDF)

S10 Table Overview of the data set of NMR ensemble structures (data set 2). The column ID holds the first two letters of the model names in our downloadable data set.
(PDF)

S11 Table Query structure matches used for the data set of successful examples (data set 7). (PDF)

S12 Table Cut-off values for all comparison methods defined using the Youden’s J statistic[85]. The thresholds were defined separately for each data set based on the corresponding ROC curves. Means and standard deviations for the cut-off values for all data sets are given together with the corresponding score and the score range (whenever applicable). The distance measures of RAPMAD and SiteAlign were transformed to the corresponding similarities.
(PDF)

S13 Table Different criteria of importance for the choice of a suitable binding site comparison method. With respect to the applicability toward predicted sites, a plus in brackets means that the predicted binding site has to be given with the corresponding coordinates of a binding site prediction as “artificial” ligand, a simple plus denotes tools that offer a way of binding site prediction. With respect to run time evaluation, “+”, “/”, “-” denote comparison algorithms that require several ns, µs, or s per comparison, respectively. With respect to the scoring, a “+” was assigned to those tools where the intervals of upper and lower whiskers of active and inactive pairs do not overlap. A “/” denotes tools where the upper and lower quartile for the pairs do not overlap. With respect to other factors, tools that were clearly outperformed by many other tools were assigned a “-”.
(PDF)

S14 Table AUC and EFs of different binding site comparison methods for data set 1. (PDF)

S15 Table AUC confidence intervals for the ROC curves of different binding site comparison methods and AUC value differences with the corresponding p-values calculated according to DeLong and co-workers[109] for data set 1. P-values below 0.05 are colored green.
(PDF)

S16 Table AUC and EFs of different binding site comparison methods for data set 1.2. (PDF)

S17 Table AUC confidence intervals for the ROC curves of different binding site comparison methods and AUC value differences with the corresponding p-values calculated according to DeLong and co-workers[109] for data set 1.2. P-values below 0.05 are colored green.
(PDF)

S18 Table AUC and EFs of different binding site comparison methods for data set 2. (PDF)

S19 Table AUC confidence intervals for the ROC curves of different binding site comparison methods and AUC value differences with the corresponding p-values calculated according to DeLong and co-workers[109] for data set 2. P-values below 0.05 are colored green.
(PDF)

S20 Table AUC and EFs of different binding site comparison methods for data set 3. (PDF)

S21 Table AUC confidence intervals for the ROC curves of different binding site comparison methods and AUC value differences with the corresponding p-values calculated according to DeLong and co-workers[109] for data set 3. P-values below 0.05 are colored green.
(PDF)

S22 Table AUC and EFs of different binding site comparison methods for data set 4. (PDF)

S23 Table AUC confidence intervals for the ROC curves of different binding site comparison methods and AUC value differences with the corresponding p-values calculated according to DeLong and co-workers[109] for data set 4. P-values below 0.05 are colored green.
(PDF)

S24 Table AUC and EFs of different binding site comparison methods for data set 5. (PDF)

S25 Table AUC confidence intervals for the ROC curves of different binding site comparison methods and AUC value differences with the corresponding p-values calculated according to DeLong and co-workers[109] for data set 5. P-values below 0.05 are colored green.
(PDF)

S26 Table AUC and EFs of different binding site comparison methods for data set 5.2. (PDF)

S27 Table AUC confidence intervals for the ROC curves of different binding site comparison methods and AUC value differences with the corresponding p-values calculated according to DeLong and co-workers[109] for data set 5.2. P-values below 0.05 are colored green.
(PDF)

S28 Table AUC and EFs of different binding site comparison methods for data set 6. (PDF)

S29 Table AUC confidence intervals for the ROC curves of different binding site comparison methods and AUC value differences with the corresponding p-values calculated according to DeLong and co-workers[109] for data set 6. P-values below 0.05 are colored green.
(PDF)

S30 Table AUC and EFs of different binding site comparison methods for data set 6.2. (PDF)

S31 Table AUC confidence intervals for the ROC curves of different binding site comparison methods and AUC value differences with the corresponding p-values calculated according to DeLong and co-workers[109] for data set 6.2. P-values below 0.05 are colored green.
(PDF)

S32 Table AUC and EFs of different binding site comparison methods for data set 7. (PDF)

S33 Table AUC confidence intervals for the ROC curves of different binding site comparison methods and AUC value differences with the corresponding p-values calculated according to DeLong and co-workers[109] for data set 7. P-values below 0.05 are colored green.
(PDF)

S1 Fig Binding site alignments and statistics of the Tanimoto coefficients for all pairs within the groups of structures with identical sequences. All figures were generated using UCSF Chimera [60].
(PNG)

S2 Fig Evaluation of different binding site comparison tools with respect to data set 1.2 including only similar binding site pairs with similar ligands (Tanimoto coefficient > 0.6). A-C) The ROC curves for residue- (A), surface- (B), and interaction-based (C) comparison methods. The name of the tool is colored according to its corresponding ROC curve. The binding site comparison tools are sorted in descending order with respect to their AUC. (B) The score RefTversky (color) led to the highest AUC values for Shaper, Shaper(PDB), VolSite/Shaper, and VolSite/Shaper(PDB). D-F) EFs for residue- (D), surface- (E), and interaction-based (F) comparison methods. A linear color gradient ranging from white for the highest value to gray to black for the lowest value was applied for the EFs at different percentages of screened data set.
(PNG)

S3 Fig Box plots to visualize the score distribution for similar and dissimilar pairs for the residue-based binding site comparison methods. The plots were created based on the data set of structures with identical sequences. The usage of RAPMAD and SiteAlign results in distances for the binding site pairs. The application of the remaining tools results in similarity scores. The values given in the plots correspond to the maximum, the upper whisker, the upper quartile, the median, the lower quartile, the lower whisker, and the minimum.
(PNG)

S4 Fig Box plots to visualize the score distribution for similar and dissimilar pairs for the surface-based binding site comparison methods. The plots were created based on the data set of structures with identical sequences. The values given in the plots correspond to the maximum, the upper whisker, the upper quartile, the median, the lower quartile, the lower whisker, and the minimum.
(PNG)

S5 Fig Box plots to visualize the score distribution for similar and dissimilar pairs for the interaction-based binding site comparison methods. The plots were created based on the data set of structures with identical sequences. The values given in the plots correspond to the maximum, the upper whisker, the upper quartile, the median, the lower quartile, the lower whisker, and the minimum.
(PNG)

S6 Fig Evaluation of different binding site comparison tools with respect to data set 3 (one, two, three, four, and five substitutions by physicochemically different residues). A-C) The ROC curves for residue- (A), surface- (B), and interaction-based (C) comparison methods. The name of the tool is colored according to its corresponding ROC curve. The binding site comparison tools are sorted in descending order with respect to their AUC. (A) PocketMatch showed the best AUC for the score PMScore_min_ (thin orange line). (B) The scores SVA, RefTversky (color), RefTversky (color), RefTversky (color), RefTversky (color), and ColorTanimoto led to the highest AUC values for ProBiS, Shaper, Shaper(PDB), VolSite/Shaper, VolSite/Shaper(PDB), and SiteHopper, respectively (thin lines). (C) The highest AUC was obtained for IsoMIF and TIFP(PDB) when using taniM and the Tanimoto coefficient, respectively (thin lines). D-F) EFs for residue- (D), surface- (E), and interaction-based (F) comparison methods. A linear color gradient ranging from white for the highest value to gray to black for the lowest value was applied for the EFs at different percentages of screened data set.
(PNG)

S7 Fig The ligand binding site of angiotensin-converting enzyme (PDB ID 4ca7 chain A). The structure is depicted in green in the surface representation (A) and the clipped surface representation (B). The ligand with the id 3EF is shown as an orange ball-and-stick model. The figure was generated using UCSF Chimera.
(PNG)

S8 Fig Evaluation of different binding site comparison tools with respect to the data set of Kahraman *et al*.[63]. A-C) The ROC curves for residue- (A), surface- (B), and interaction-based (C) comparison methods. The name of the tool is colored according to its corresponding ROC curve. The binding site comparison tools are sorted in descending order with respect to the AUC. (A) The best AUC for SiteAlign resulted from the d1 distance (thin red line). (B) For ProBiS, SiteEngine, and SiteHopper the scores SVA, LowResolutionScore, and ShapeTanimoto yielded the best AUC values (thin lines). (C) For TIFP(PDB), the use of the Hamming distance led to the best results with respect to AUC (thin line). D-F) EFs for residue- (D), surface- (E), and interaction-based (F) comparison methods. A linear color gradient ranging from white for the highest value to gray to black for the lowest value was applied for the EFs at different percentages of screened data set.
(PNG)

S9 Fig Evaluation of different binding site comparison tools with respect to the data set of Barelier *et al*.[64] (cofactors were included for comparison wherever possible). A-C) The ROC curves for residue- (A), surface- (B), and interaction-based (C) comparison methods. The name of the tool is colored according to its corresponding ROC curve. The binding site comparison tools are sorted in descending order with respect to the AUC. (A) The thin red line represents the resulting ROC curve for SiteAlign when using the distance d1. (B) Thin lines represent the ROC curves for ProBiS, Shaper, Shaper(PDB), VolSite/Shaper, VolSite/Shaper(PDB), SiteEngine and SiteHopper when using the scoring schemes SVA, FitTversky (color), FitTversky (color), RefTversky (color), Tanimoto (fit), DistanceScore, and ShapeTanimoto, respectively. (C) The thin line represents the resulting ROC curve for IsoMIF and the taniMW score. D-F) EFs for residue- (D), surface- (E), and interaction-based (F) comparison methods. A linear color gradient ranging from white for the highest value to gray to black for the lowest value was applied for the EFs at different percentages of screened data set.
(PNG)

S10 Fig Failure rates of all residue-based (top), surface-based (center), and interaction-based (bottom) binding site comparison methods for all data sets analyzed in this study. (PNG)
