# Supplementary material for: A benchmark driven guide to binding site comparison: An exhaustive evaluation using tailor-made data sets (ProSPECCTs)
Source: PLoS Comput Biol. 2018 Nov 8;14(11):e1006483. doi: 10.1371/journal.pcbi.1006483 (PMC6224041; doi:10.1371/journal.pcbi.1006483)
Supplement: S1 Text — (PDF) [file pcbi.1006483.s001.pdf]

S1 Text. Brief description of the analyzed binding site comparison methods

Binding Site Modeling Based on Residues

The binding site residues' properties are represented by means of pseudocenters for comparisons using Cavbase[1,2] and RAPMAD[3] (Rapid Pocket Matching using Distances). The physicochemical properties of binding site residue atoms - as detected by an implementation of the pocket detection approach LIGSITE[4] - are assigned with the following types: hydrogen bond acceptor or donor, mixed hydrogen bond acceptor/donor, center of an aromatic ring, π electron center, aliphatic moiety, and metal ion. First, the pseudocenters are matched to identify suitable superposition possibilities while the following step transforms the pseudocenters into undirected graphs. The overlap of the final match is calculated by using the number of matching surface points within a distance of 1 Å (Cavbase similarity). In contrast, in RAPMAD the pseudocenters are divided into seven property subsets and the centroid and pseudocenter that is closest to this centroid are determined for each set. The Euclidean distances of those two reference points to all pseudocenters enables the generation of distance histograms with a bin size of 0.4 Å for comparison. The tool returns a distance score for the compared cavities (RAPMAD distance) based on the histogram distributions. The outcome of both methods was shown to be comparable with respect to classification performance. Both methods can solely be applied to binding sites.

For FuzCav[5] comparisons, the Cα atom coordinates of binding site residues are labelled with six pharmacophoric properties (hydrogen bond acceptor and donor, positive and negative ionizable, aromatic, aliphatic). The final fingerprint representation encodes all unique pharmacophore triplets together with the binned Cα atom distances (0-4.8, 4.8-7.2, 7.2-9.5, 9.5-11.9, 11.9-14.3 Å). A fingerprint for 4,833 possible triplets is used for comparison. The final score is calculated by dividing the number of common fingerprint strings by the minimum number of non-zero fingerprint strings of the compared binding sites (FuzCav similarity).

In PocketMatch[6], the binding site residues are grouped according to their physicochemical properties (0: Ala, Val, Ile, Leu, Gly, Pro; 1: Lys, Arg, His; 2: Asp, Asn, Glu, Gln; 3: Tyr, Phe, Trp; 4: Cys, Ser, Thr) and are represented by three types of points (Cα, Cβ, Ccentroid – the geometric center of the side chain atoms). A binning of all pairs of point distances (group-type pairs and point-type pairs) enables a subsequent comparison and alignment of pairs of sorted distance sequences. The scoring scheme recommended by the developers consists in dividing the number of distance pair matches by the total number of distance pairs of the larger cavity ($PMScore_{max}$). Additionally, the $PMScore_{min}$ is returned which takes the total number of distance pairs of the smaller cavity into account.

The description of the binding site used by SiteAlign[7] includes topological properties (distance Cβ – sphere center, side chain orientation, size) and physicochemical properties (hydrogen bond acceptor or donor, charge, aromatic, aliphatic). They are projected on a discretized 80 triangle sphere with its origin in the cavity center by deriving a geometrical vector from the Cα atom of each binding site residue to the sphere center. The descriptors are mapped on the respective triangle to derive a binding site fingerprint. The alignment thrives to optimize the number of possible similarities between both sites. For the final scoring, normalized similarities per triangle and descriptor are calculated and finally averaged for each triangle. Four distances (d1, d2, d3, d4) are calculated by subtracting the following similarities from 1: the triangle similarity sum which is divided by the number of non-zero triangles for one of both sites (s1), the number of non-zero triangles for both sites (s2), the number of non-zero triangles which lead to local distances smaller than or equal to 0.2 for both sites (s3), and the number of non-zero triangles with polar character for both sites (s4). Due to the underlying methodology, this method should not be applied to compare binding sites to proteins.

The previously developed method SOIPPA (Sequence Order Independent Profile-Profile Alignments)[8] constitutes the basis for SMAP[9]. A mesh surface is generated by a Delaunay

triangulation of the binding site's Cα atoms. Each Cα atom is assigned a normal vector that is perpendicular to the surface. Moreover, a PSI-BLAST[10] search is conducted to derive a position-specific scoring matrix and assign a probability distribution to each Cα atom. The annotated ligand binding site is finally represented as encoded graph based on the regular tessellation. The McLachlan[11] similarity matrix is used to describe the physicochemical and evolutionary relationship of the matched cavities and the angle and distance between two matched atoms is used as geometric quality criterion. The resulting score (raw score) is returned together with the RMSD, the template and query coverage, and the Tanimoto coefficient which describes the ratio of the overlapping pocket volume and the union pocket volumes of both sites. In contrast to SOIPPA, a statistical model implemented in SMAP assists to assess the statistical significance of a match by the calculation of p-values on the basis of an extreme value distribution. The method is applicable toward local binding site similarities as well as those between cavities and complete proteins.

Although TM-align[12] was developed to superpose protein structures, the method's successful application in various site comparison scenarios led to the inclusion of the method in our analysis. The comparison is based on the Cα coordinates and the underlying secondary structures elements based on the DSSP (dictionary of protein secondary structure) definition[13]. The TM-score does not consider the physicochemical nature of the matched residues. It represents the sum of distances between aligned residues after optimal spatial superposition. It is noteworthy that the algorithm is sequence order dependent, i.e. a discontinuous alignment of binding site residues cannot be achieved.

Binding Site Modeling Based on Surface Properties

ProBiS (Protein Binding Site)[14] compares binding sites using graphs. Solvent accessible surface atoms of the cavity are annotated by the nature of their underlying functional group properties (hydrogen bond acceptor or donor, mixed hydrogen bond acceptor/donor, aromatic,

and aliphatic). The atoms comprise the vertices of a labelled graph that is connected by edges. Subgraphs are derived by connecting vertices that are within a 15 Å distance for the use of complete protein structures. Various filtering steps enable a fast comparison. Finally, the tool returns an expectation value (e-value) according to the Karlin-Altschul equation[15], the surface vector angle between the two normal vectors of the matched protein surfaces (SVA), an RMSD of the aligned surface vertices, a z-score a (a score calculated from the number of matched vertices, the e-value, and the RMSD), and the alignment score that is composed of the aforementioned terms. The method enables the comparison of binding sites to proteins, binding sites to binding sites and proteins to proteins to identify local similarities.

In SiteEngine[16], physicochemical properties are determined similar to the Cavbase approach. The binding site residue atoms (4 Å of the ligand atoms) are grouped according to their potential ligand interaction. A Connolly surface of the binding site is constructed and pseudocenters with at least one surface exposed atom are retained. Pseudocenter triplets are generated and encoded with the respective side lengths and a physicochemical index. A comparison via hashing and an elaborate match analysis and rescoring of possible transformations enable the identification of binding site similarities. The found matches per pair are filtered by applying an additional graph matching procedure to identify the maximum weight match. The obtained results can be ranked according to a LowResolutionScore, an OverallSurfaceScore, a DistanceScore, a CurvatureScore, or the TotalScore that is computed by combining the four aforementioned scores. The tool allows for the comparison of pockets as well as protein structures and pockets.

For SiteHopper[17] comparisons, a ligand-occupied cavity is represented as a 3D shape that is “colored” by chemical properties of the underlying binding site residues. This is achieved with the help of the Shape[18] and Spicoli[19] toolkits of OpenEye. After the alignment of binding site patches the maximum overlap of atom-centered Gaussian functions is calculated.

The final so-called Patch Score combines a measure for physicochemical similarity (ColorTanimoto) and a measure to estimate the surface shape similarity (ShapeTanimoto). The software enables a comparison of protein pockets, but does not support the comparison of pockets against protein structures or vice versa. In a recent publication it was shown that the tool is also applicable to newly identified binding sites.[20]

VolSite is used to generate the binding site representation used for a comparison using Shaper[21]. A cube is placed at the ligand's center of mass and filled with a grid. A predicted binding site can also be used for the binding site definition. "IN" and "OUT" properties are assigned to the grid cells in dependence of the presence or absence of protein atoms and their corresponding buriedness. The resulting site points are assigned a pharmacophoric property (hydrogen bond acceptor or donor, mixed hydrogen bond acceptor/donor, hydrophobic, aromatic, positive or negative ionizable). The resulting VolSite attributes are additionally used for druggability prediction based on an SVM (Support Vector Machine) algorithm. Druggable annotated binding sites can be subsequently used for Shaper comparisons. The tool is designed for pharmacophore-annotated cavity shape comparison. Various similarity measures are provided: the color and fit Tanimoto as well as their sum (combo) and the color and fit Tversky index as well as their sum (combo) with a higher weight on the reference cavity (RefTversky) or the superposed cavity (FitTversky).

Binding Site Modeling Based on Protein-Ligand Interactions

The software IsoMIF[22] is based on molecular interaction fields (MIFs) which describe the putative interactions of binding site residues with ligand atoms. Pockets are detected by the GetCleft[23] algorithm which transforms the cavity into a grid with a 0.5 Å spacing with a distance of 2.5 to 4.0 Å to any protein non-hydrogen atom. Moreover, cavities can be defined by a bound ligand. Each grid point is labelled by the interaction potential energy for six different chemical probes (hydrophobic, aromatic, hydrogen bond acceptor or donor, positively

or negatively charged). Protein atom types are assigned according to the original SYBYL atom types[24] and an additional n.his atom type for the histidine nitrogen atoms that can exist in different protonation states (hydrogen bond acceptor and donor). Based on a given probe interaction energy threshold, probes in the vertices are neglected or retained. For donor and acceptor probes as well as aromatic interactions, additional threshold angles were introduced. The final MIF is the set of interaction vectors at all vertex positions within the grid. The similarity score is calculated as the Tanimoto similarity based on the number of common probes and the number of energetically significant probes for each of the two binding sites. Additionally, an RMSD value is given. In addition to the Tanimoto similarity (tani), a match is characterized by three further similarity scores (taniM, taniMW, and taniNormNodes).

For a comparison using KRIPO (Key Representation of Interaction in Pockets)[25] binding sites are extracted and described based on bound ligand fragments or ligands. All residues within 6 Å of a ligand or ligand fragment atom are defined as cavity residues. Intermolecular interaction features (hydrogen bond acceptors or donors, aromatic T- and π-interactions, hydrophobic interactions, salt bridges) are described by placing them at distinct geometric positions with respect to the underlying residue according to predefined rules (e.g. hydrogen bond donor vectors are placed at a distinct position along the vectors defined by the N-H and O-H bonds). Distances between pharmacophoric features are binned according to a non-linear binning scheme and 3-point pharmacophore fingerprints are generated. Each triplet is encoded by the features and the binned distances. The feature opposite the shortest distance is prioritized to provide unique codes. An additional fuzziness was introduced by setting more than one bit in the fingerprint for one pharmacophore triplet, i.e. three distance are encoded for each of the three feature distances. This leads to 27 bits which represent one triplet. A modified Tanimoto coefficient according to Fligner *et al.*[26] was introduced as similarity measure to reduce the size bias.

Protein and ligand atoms are assigned seven pharmacophoric properties (hydrogen bond acceptor or donor, hydrophobic, aromatic, positive or negative ionizable, metal) for a comparison with TIFP (Interaction Fingerprint Triplets)[27]. The detection of interactions is based on geometric rules including distance and angle thresholds. The interactions are: hydrophobic (hydrophobic - hydrophobic), face-to-face and edge-to-face (aromatic - aromatic), hydrogen bonds and weak hydrogen bonds ((weak) donor - (weak) acceptor), ionic (anion - cation), π-cation (cation - aromatic) and metal coordination (metal - acceptor). The overall interaction pattern comprises the two interacting atoms of protein and ligand as well as interaction pseudoatoms (the geometric center of two interacting atoms). The user can decide for a suitable comparison mode. Interaction triplets are derived based on the interaction properties and distances (discretized in six intervals) and the derived interaction fingerprint triplets are compared. The similarities can be evaluated by different fingerprint similarity measures. Using TIFP, six fingerprint similarity measures can be obtained to rank the binding site pairs: Tanimoto similarity, Hamming distance, two Tversky indices (Ref Tversky, Fit Tversky), Dice index, and Soergel similarity. In contrast, Grim (Graph interaction matching)[27] uses a graph matching algorithm for the comparison of interaction pseudoatoms. The relative frequencies of the different interaction types are taken into account by weight assignments based on the observed interactions in the sc-PDB[28]. The final equation to derive the Grscore (Graph-alignment score) was derived from the empirical analysis of 900 similar and 900 dissimilar protein-ligand complexes. It results from different contributions of the number of matched InterLig interaction pseudoatoms, the number of matched centered interaction pseudoatoms, the sum of pair weights in clique divided by the sum of all possible pair weights, the number of matched InterProt interaction pseudoatoms, the RMSD of the matched clique, and the absolute value of the difference in the number of interaction pseudoatoms between reference and query.

REFERENCES

1. Schmitt S, Hendlich M, Klebe G. From structure to function: A new approach to detect functional similarity among proteins independent from sequence and fold homology. Angew. Chem. Int. Ed. 2001;40(17):3141–4. doi: 10.1002/1521-3773(20010903)40:17<3141:AID-ANIE3141>3.0.CO;2-X.
2. Schmitt S, Kuhn D, Klebe G. A new method to detect related function among proteins independent of sequence and fold homology. J Mol Biol. 2002;323(2):387–406. PubMed PMID: 12381328.
3. Krotzky T, Grunwald C, Egerland U, Klebe G. Large-scale mining for similar protein binding pockets: with RAPMAD retrieval on the fly becomes real. J Chem Inf Model. 2015;55(1):165–79. doi: 10.1021/ci5005898. PubMed PMID: 25474400.
4. Hendlich M, Rippmann F, Barnickel G. LIGSITE: automatic and efficient detection of potential small molecule-binding sites in proteins. J Mol Graph Model. 1997;15(6):359-63, 389. PubMed PMID: 9704298.
5. Weill N, Rognan D. Alignment-free ultra-high-throughput comparison of druggable protein-ligand binding sites. J Chem Inf Model. 2010;50(1):123–35. doi: 10.1021/ci900349y. PubMed PMID: 20058856.
6. Yeturu K, Chandra N. PocketMatch: a new algorithm to compare binding sites in protein structures. BMC Bioinformatics. 2008;9:543. doi: 10.1186/1471-2105-9-543. PubMed PMID: 19091072.
7. Schalon C, Surgand J-S, Kellenberger E, Rognan D. A simple and fuzzy method to align and compare druggable ligand-binding sites. Proteins. 2008;71(4):1755–78. doi: 10.1002/prot.21858. PubMed PMID: 18175308.
8. Xie L, Bourne PE. Detecting evolutionary relationships across existing fold space, using sequence order-independent profile-profile alignments. Proc Natl Acad Sci U S A. 2008;105(14):5441–6. doi: 10.1073/pnas.0704422105. PubMed PMID: 18385384.
9. Xie L, Xie L, Bourne PE. A unified statistical model to support local sequence order independent similarity searching for ligand-binding sites and its application to genome-based drug discovery. Bioinformatics. 2009;25(12):i305-12. doi: 10.1093/bioinformatics/btp220. PubMed PMID: 19478004.
10. Altschul SF, Madden TL, Schäffer AA, Zhang J, Zhang Z, Miller W, et al. Gapped BLAST and PSI-BLAST: A new generation of protein database search programs. Nucleic Acids Res. 1997;25(17):3389–402. PubMed PMID: 9254694.
11. McLachlan AD. Tests for comparing related amino-acid sequences. Cytochrome c and cytochrome c 551. J Mol Biol. 1971;61(2):409–24. PubMed PMID: 5167087.
12. Zhang Y, Skolnick J. TM-align: a protein structure alignment algorithm based on the TM-score. Nucleic Acids Res. 2005;33(7):2302–9. doi: 10.1093/nar/gki524. PubMed PMID: 15849316.
13. Kabsch W, Sander C. Dictionary of protein secondary structure: pattern recognition of hydrogen-bonded and geometrical features. Biopolymers. 1983;22(12):2577–637. doi: 10.1002/bip.360221211. PubMed PMID: 6667333.
14. Konc J, Janežič D. ProBiS algorithm for detection of structurally similar protein binding sites by local structural alignment. Bioinformatics. 2010;26(9):1160–8. doi: 10.1093/bioinformatics/btq100. PubMed PMID: 20305268.

15. Karlin S, Altschul SF. Methods for assessing the statistical significance of molecular sequence features by using general scoring schemes. Proc Natl Acad Sci U S A. 1990;87(6):2264–8. PubMed PMID: 2315319.
16. Shulman-Peleg A, Nussinov R, Wolfson HJ. Recognition of functional sites in protein structures. J Mol Biol. 2004;339(3):607–33. doi: 10.1016/j.jmb.2004.04.012. PubMed PMID: 15147845.
17. Batista J, Hawkins PCD, Tolbert R, Geballe MT. SiteHopper - a unique tool for binding site comparison. J Cheminform. 2014;6(Suppl 1):P57. doi: 10.1186/1758-2946-6-S1-P57.
18. Shape Toolkit. Santa Fe, NM: OpenEye Scientific Software.
19. Spicoli Toolkit. Santa Fe, NM: OpenEye Scientific Software.
20. Meyers J, Brown N, Blagg J. Mapping the 3D structures of small molecule binding sites. J Cheminform. 2016;8(1):235. doi: 10.1186/s13321-016-0180-0.
21. Desaphy J, Azdimousa K, Kellenberger E, Rognan D. Comparison and druggability prediction of protein-ligand binding sites from pharmacophore-annotated cavity shapes. J Chem Inf Model. 2012;52(8):2287–99. doi: 10.1021/ci300184x. PubMed PMID: 22834646.
22. Chartier M, Najmanovich R. Detection of binding site molecular interaction field similarities. J Chem Inf Model. 2015;55(8):1600–15. doi: 10.1021/acs.jcim.5b00333. PubMed PMID: 26158641.
23. Gaudreault F, Morency L-P, Najmanovich RJ. NRGsuite: a PyMOL plugin to perform docking simulations in real time using FlexAID. Bioinformatics. 2015;31(23):3856–8. doi: 10.1093/bioinformatics/btv458. PubMed PMID: 26249810.
24. Clark M, Cramer RD, van Opdenbosch N. Validation of the general purpose tripos 5.2 force field. J Comput Chem. 1989;10(8):982–1012. doi: 10.1002/jcc.540100804.
25. Wood DJ, Vlieg J de, Wagener M, Ritschel T. Pharmacophore fingerprint-based approach to binding site subpocket similarity and its application to bioisostere replacement. J Chem Inf Model. 2012;52(8):2031–43. doi: 10.1021/ci3000776. PubMed PMID: 22830492.
26. Fligner MA, Verducci JS, Blower PE. A modification of the Jaccard–Tanimoto similarity index for diverse selection of chemical compounds using binary strings. Technometrics. 2002;44(2):110–9. doi: 10.1198/004017002317375064.
27. Desaphy J, Raimbaud E, Ducrot P, Rognan D. Encoding protein-ligand interaction patterns in fingerprints and graphs. J Chem Inf Model. 2013;53(3):623–37. doi: 10.1021/ci300566n. PubMed PMID: 23432543.
28. Kellenberger E, Muller P, Schalon C, Bret G, Foata N, Rognan D. sc-PDB: an annotated database of druggable binding sites from the Protein Data Bank. J Chem Inf Model. 2006;46(2):717–27. doi: 10.1021/ci050372x. PubMed PMID: 16563002.
