# Supplementary material for: A benchmark driven guide to binding site comparison: An exhaustive evaluation using tailor-made data sets (ProSPECCTs)
Source: PLoS Comput Biol. 2018 Nov 8;14(11):e1006483. doi: 10.1371/journal.pcbi.1006483 (PMC6224041; doi:10.1371/journal.pcbi.1006483)
Supplement: S1 Table — (PDF) [file pcbi.1006483.s002.pdf]

S1 Table. Summary of binding site comparison tools and the evaluation data sets used to indicate their applicability and strengths.

method	benchmark data sets (examples)
Cavbase[1,2]	query-based evaluation
FuzCav[3]	set of non-redundant similar and dissimilar binding sites based on SiteAlign comparisons,<br>clustering of ATP and protease binding sites
Grim[4]	similar and dissimilar protein ligand complexes based on a Shaper comparison,<br>rescoring of docking poses
IsoMIF[5]	Kahraman data set[6],<br>SOIPPA data set[7] as modified by Brylinski *et al.* (228 adenine containing binding sites and 91 true negatives)[8]
KRIPO[9]	fragments at least 20 times in PDB,<br>10,000 random fragment pairs within 200 diverse PDB ligand fragments (decoy)
PocketMatch[10]	similar SCOP classification (family level),<br>sites for identical ligands in multiple protein subunits,<br>binding sites in different protein structures complexed with CIT, MTX, MK1, or PGA
ProBiS[11]	test sets for binding site detection
RAPMAD[12]	data set of ATP, NAD, and FAD binding sites[13],<br>Hoffmann data set[14]
VolSite/Shaper[15]	clustering of ATP and protease binding sites
SiteAlign[16]	376 non-redundant related binding site pairs (EC classes) from the sc-PDB[17]
SiteEngine[18]	query-based searches including adenine binding proteins, proteases, estradiol binding proteins, etc. (screening of databases of binding sites or complete structures with known binding sites, or databases of binding sites with complete protein structures)
SiteHopper[19]	query-based searches of sc-PDB[17] entries including therapeutically relevant targets
SMAP[20]	247 non-redundant protein chains binding an adenine-containing ligand and 101 non-redundant protein chains believed not to bind an adenine-containing molecule
TIFP[4]	similar and dissimilar protein-ligand complexes based on a Shaper comparison,<br>rescoring of docking poses
TM-align[21]	200 non-homologous PDB proteins (comparison to different protein structure alignment algorithms)

REFERENCES

1. Schmitt S, Hendlich M, Klebe G. From structure to function: A new approach to detect functional similarity among proteins independent from sequence and fold homology. Angew. Chem. Int. Ed. 2001;40(17):3141–4. doi: 10.1002/1521-3773(20010903)40:17<3141:AID-ANIE3141>3.0.CO;2-X.
2. Schmitt S, Kuhn D, Klebe G. A new method to detect related function among proteins independent of sequence and fold homology. J Mol Biol. 2002;323(2):387–406. PubMed PMID: 12381328.
3. Weill N, Rognan D. Alignment-free ultra-high-throughput comparison of druggable protein-ligand binding sites. J Chem Inf Model. 2010;50(1):123–35. doi: 10.1021/ci900349y. PubMed PMID: 20058856.
4. Desaphy J, Raimbaud E, Ducrot P, Rognan D. Encoding protein-ligand interaction patterns in fingerprints and graphs. J Chem Inf Model. 2013;53(3):623–37. doi: 10.1021/ci300566n. PubMed PMID: 23432543.
5. Chartier M, Najmanovich R. Detection of binding site molecular interaction field similarities. J Chem Inf Model. 2015;55(8):1600–15. doi: 10.1021/acs.jcim.5b00333. PubMed PMID: 26158641.
6. Kahraman A, Morris RJ, Laskowski RA, Favia AD, Thornton JM. On the diversity of physicochemical environments experienced by identical ligands in binding pockets of unrelated proteins. Proteins. 2010;78(5):1120–36. doi: 10.1002/prot.22633. PubMed PMID: 19927322.
7. Xie L, Bourne PE. Detecting evolutionary relationships across existing fold space, using sequence order-independent profile-profile alignments. Proc Natl Acad Sci U S A. 2008;105(14):5441–6. doi: 10.1073/pnas.0704422105. PubMed PMID: 18385384.
8. Brylinski M. eMatchSite: sequence order-independent structure alignments of ligand binding pockets in protein models. PLoS Comput Biol. 2014;10(9):e1003829. doi: 10.1371/journal.pcbi.1003829. PubMed PMID: 25232727.
9. Wood DJ, Vlieg J de, Wagener M, Ritschel T. Pharmacophore fingerprint-based approach to binding site subpocket similarity and its application to bioisostere replacement. J Chem Inf Model. 2012;52(8):2031–43. doi: 10.1021/ci3000776. PubMed PMID: 22830492.
10. Yeturu K, Chandra N. PocketMatch: a new algorithm to compare binding sites in protein structures. BMC Bioinformatics. 2008;9:543. doi: 10.1186/1471-2105-9-543. PubMed PMID: 19091072.
11. Konc J, Janežič D. ProBiS algorithm for detection of structurally similar protein binding sites by local structural alignment. Bioinformatics. 2010;26(9):1160–8. doi: 10.1093/bioinformatics/btq100. PubMed PMID: 20305268.
12. Krotzky T, Grunwald C, Egerland U, Klebe G. Large-scale mining for similar protein binding pockets: with RAPMAD retrieval on the fly becomes real. J Chem Inf Model. 2015;55(1):165–79. doi: 10.1021/ci5005898. PubMed PMID: 25474400.
13. Krotzky T, Fober T, Hüllermeier E, Klebe G. Extended graph-based models for enhanced similarity search in Cavbase. IEEE/ACM Trans Comput Biol Bioinform. 2014;11(5):878–90. doi: 10.1109/TCBB.2014.2325020. PubMed PMID: 26356860.
14. Hoffmann B, Zaslavskiy M, Vert J-P, Stoven V. A new protein binding pocket similarity measure based on comparison of clouds of atoms in 3D: application to ligand prediction.

BMC Bioinformatics. 2010;11:99. doi: 10.1186/1471-2105-11-99. PubMed PMID: 20175916.

15. Desaphy J, Azdimousa K, Kellenberger E, Rognan D. Comparison and druggability prediction of protein-ligand binding sites from pharmacophore-annotated cavity shapes. J Chem Inf Model. 2012;52(8):2287–99. doi: 10.1021/ci300184x. PubMed PMID: 22834646.
16. Schalon C, Surgand J-S, Kellenberger E, Rognan D. A simple and fuzzy method to align and compare druggable ligand-binding sites. Proteins. 2008;71(4):1755–78. doi: 10.1002/prot.21858. PubMed PMID: 18175308.
17. Kellenberger E, Muller P, Schalon C, Bret G, Foata N, Rognan D. sc-PDB: an annotated database of druggable binding sites from the Protein Data Bank. J Chem Inf Model. 2006;46(2):717–27. doi: 10.1021/ci050372x. PubMed PMID: 16563002.
18. Shulman-Peleg A, Nussinov R, Wolfson HJ. SiteEngines: recognition and comparison of binding sites and protein-protein interfaces. Nucleic Acids Res. 2005;33(Web Server issue):W337-41. doi: 10.1093/nar/gki482. PubMed PMID: 15980484.
19. Batista J, Hawkins PCD, Tolbert R, Geballe MT. SiteHopper - a unique tool for binding site comparison. J Cheminform. 2014;6(Suppl 1):P57. doi: 10.1186/1758-2946-6-S1-P57.
20. Xie L, Xie L, Bourne PE. A unified statistical model to support local sequence order independent similarity searching for ligand-binding sites and its application to genome-based drug discovery. Bioinformatics. 2009;25(12):i305-12. doi: 10.1093/bioinformatics/btp220. PubMed PMID: 19478004.
21. Zhang Y, Skolnick J. TM-align: a protein structure alignment algorithm based on the TM-score. Nucleic Acids Res. 2005;33(7):2302–9. doi: 10.1093/nar/gki524. PubMed PMID: 15849316.
