# Supplementary material for: A benchmark driven guide to binding site comparison: An exhaustive evaluation using tailor-made data sets (ProSPECCTs)
Source: PLoS Comput Biol. 2018 Nov 8;14(11):e1006483. doi: 10.1371/journal.pcbi.1006483 (PMC6224041; doi:10.1371/journal.pcbi.1006483)
Supplement: S2 Table — (PDF) [file pcbi.1006483.s003.pdf]

S2 Table. Statistics of binding site RMSD values for the groups of structures with identical sequences.

PDB ID.chain	RMSD	mean [Å]	standard deviation [Å]	minimum [Å]	maximum [Å]
1kmv.A	Cα	0.46	0.19	0.09	1.05
	all atoms	1.24	0.32	0.37	2.09
1odm.A	Cα	0.14	0.05	0.04	0.28
	all atoms	0.49	0.24	0.08	1.05
2qxw.A	Cα	0.55	0.22	0.09	1.16
	all atoms	0.85	0.27	0.32	1.41
3f17.A	Cα	0.34	0.11	0.13	0.71
	all atoms	0.72	0.19	0.26	1.13
3rm2.H	Cα	0.28	0.10	0.07	0.74
	all atoms	0.58	0.15	0.16	1.04
3t10.A	Cα	1.27	0.72	0.09	2.53
	all atoms	1.38	0.65	0.13	2.39
3u5l.A	Cα	0.25	0.10	0.06	0.47
	all atoms	0.44	0.13	0.11	0.67
3u9w.A	Cα	0.18	0.04	0.09	0.31
	all atoms	0.36	0.08	0.10	0.53
4bfz.A	Cα	0.82	0.32	0.15	1.44
	all atoms	1.18	0.32	0.54	1.72
4buu.A	Cα	0.59	0.50	0.08	1.44
	all atoms	1.08	0.57	0.26	2.03
4ca7.A	Cα	0.14	0.03	0.07	0.20
	all atoms	0.35	0.11	0.15	0.56
4fpt.A	Cα	0.27	0.08	0.04	0.51
	all atoms	0.61	0.12	0.13	0.91
