# Supplementary material for: A benchmark driven guide to binding site comparison: An exhaustive evaluation using tailor-made data sets (ProSPECCTs)
Source: PLoS Comput Biol. 2018 Nov 8;14(11):e1006483. doi: 10.1371/journal.pcbi.1006483 (PMC6224041; doi:10.1371/journal.pcbi.1006483)
Supplement: S3 Table — (PDF) [file pcbi.1006483.s004.pdf]

S3 Table. Statistics of the Tanimoto coefficients for all ligand pairs within the reduced data set 1 which results from the exclusion of highly dissimilar ligands within each group.

PDB ID.chain	mean	standard deviation	minimum	maximum
1kmv.A	0.79	0.00	0.79	0.79
1odm.A	0.72	0.09	0.63	1.00
2qxw.A	1.00	0.00	1.00	1.00
3f17.A	0.75	0.00	0.75	0.76
3rm2.H	0.71	0.06	0.63	0.86
3t10.A	0.73	0.11	0.65	0.96
3u5l.A	1.00	0.00	1.00	1.00
3u9w.A	0.67	0.02	0.64	0.69
4bfz.A	0.72	0.10	0.61	0.83
4buu.A	0.68	0.00	0.68	0.68
4ca7.A	0.86	0.11	0.79	1.00
4fpt.A	0.73	0.03	0.69	0.75
