# Supplementary material for: A benchmark driven guide to binding site comparison: An exhaustive evaluation using tailor-made data sets (ProSPECCTs)
Source: PLoS Comput Biol. 2018 Nov 8;14(11):e1006483. doi: 10.1371/journal.pcbi.1006483 (PMC6224041; doi:10.1371/journal.pcbi.1006483)
Supplement: S4 Table — (PDF) [file pcbi.1006483.s005.pdf]

S4 Table. Results of the Welch's[1] two-sample t-test for the active and inactive pairs of data set 1.

method (score)	t	degrees of freedom	p-value <	95% confidence interval	mean for inactive pairs	mean for active pairs
Cavbase (similarity score)	-183.48	13,446	2.20E-16	-48.32, -47.30	4.704	52.517
FuzCav (similarity score)	-134.00	13,599	2.20E-16	-0.20, -0.20	0.153	0.353
FuzCav (PDB) (similarity score)	-133.88	13,598	2.20E-16	-0.20, -0.20	0.153	0.352
Pocket-Match ($PMScore_{max}$)	-126.01	13,603	2.20E-16	-0.30, -0.29	0.045	0.339
RAPMAD (distance score)	99.33	27,711	2.20E-16	31.46, 32.73	57.437	25.339
SiteAlign (distance d3)	357.81	15,040	2.20E-16	0.22, 0.23	0.264	0.039
SMAP (RawScore)	-421.05	13,538	2.20E-16	-156.26, -154.81	35.966	191.502
TM-align (TM-score)	-794.49	14,084	2.20E-16	-0.65, -0.64	0.244	0.889
ProBiS (Alignment Score)	-517.08	16,532	2.20E-16	-12.23, -12.14	-0.570	11.618
Shaper (Tanimoto combo)	-203.55	14,691	2.20E-16	-0.46, -0.45	0.694	1.152
Shaper (PDB) (Tanimoto combo)	-206.41	14,538	2.20E-16	-0.46, -0.45	0.700	1.153
SiteEngine (Curvature Score)	-228.66	13,614	2.20E-16	-1,626.11, -1,598.46	306.327	1,918.611
SiteHopper (PatchScore)	-318.29	13,621	2.20E-16	-1.67, -1.65	0.634	2.297
VolSite/Shaper (Tanimoto combo)	-189.34	14,083	2.20E-16	-0.46, -0.45	0.703	1.161
VolSite/Shaper (PDB) (Tanimoto combo)	-189.70	14,066	2.20E-16	-0.46, -0.45	0.704	1.162
Grim (Grscore)	-56.46	16,208	2.20E-16	-0.04, -0.04	0.551	0.593
Grim (PDB) (Grscore)	-11.07	16,041	2.20E-16	-0.01, -0.01	0.553	0.562
IsoMIF (tani)	-75.05	14,823	2.20E-16	-0.14, -0.13	0.396	0.529
KRIPO (similarity score)	-149.46	14,115	2.20E-16	-0.15, -0.14	0.436	0.579
TIFP (Soergel)	-59.79	14,968	2.20E-16	-0.12, -0.11	0.138	0.255
TIFP (PDB) (Soergel)	-26.52	15,162	2.20E-16	-0.05, -0.05	0.109	0.160

REFERENCES

1. Welch BL. The generalization of 'student's' problem when several different population variances are involved. Biometrika. 1947;34(1-2):28–35. doi: 10.1093/biomet/34.1-2.28.
