# Supplementary material for: A benchmark driven guide to binding site comparison: An exhaustive evaluation using tailor-made data sets (ProSPECCTs)
Source: PLoS Comput Biol. 2018 Nov 8;14(11):e1006483. doi: 10.1371/journal.pcbi.1006483 (PMC6224041; doi:10.1371/journal.pcbi.1006483)
Supplement: S6 Table — Mean, standard deviation, minimum, and maximum are given for four descriptors of the binding sites as defined by the ligand. (PDF) [file pcbi.1006483.s007.pdf]

S6 Table. Binding site descriptors for all NMR ensembles of data set 2 as calculated with DoGSite[1]. Mean, standard deviation, minimum, and maximum are given for four descriptors of the binding sites as defined by the ligand.

PDB ID. chain	number of site atoms	number of hydrogen bond acceptor atoms	number of hydrogen bond donor atoms	number of aromatic atoms
1cz2.A	184±27, 146, 238	26±5, 20, 34	21±5, 14, 34	6±2, 2, 10
1diu.A	275±29, 237, 334	43±5, 35, 55	55±5, 49, 66	30±2, 27, 35
1eio.A	281±29, 226, 304	47±7, 35, 55	47±6, 39, 56	54±6, 42, 61
1j5i.A	96±47, 59, 256	22±8, 14, 52	16±7, 10, 46	5±5, 0, 16
1kgl.A	185±21, 150, 233	27±3, 22, 34	26±4, 19, 34	32±3, 26, 37
1mux.A	70±34, 42, 205	9±7, 2, 37	5±5, 2, 24	10±3, 3, 24
1t84.A	163±36, 112, 247	26±7, 16, 44	24±5, 17, 35	22±4, 16, 29
1tvc.A	375±151, 251, 686	57±25, 34, 110	54±26, 32, 109	43±13, 22, 67
1yho.A	344±112, 179, 537	55±18, 25, 87	53±18, 25, 82	48±12, 26, 77
2jt2.A	235±37, 177, 337	41±6, 33, 61	44±7, 32, 63	44±8, 30, 58
2k31.A	142±9, 129, 161	19±2, 14, 23	23±2, 16, 27	23±2, 19, 26
2k5t.A	157±50, 69, 259	22±8, 9, 38	25±9, 10, 41	15±7, 4, 29
2l0x.A	98±40, 33, 165	19±8, 7, 33	19±8, 6, 32	5±3, 0, 11
2l2s.A	245±61, 123, 335	38±10, 18, 57	36±11, 14, 53	42±4, 32, 49
2l8r.A	199±21, 156, 230	32±5, 24, 39	34±4, 27, 43	6±2, 2, 10
2lzg.A	193±73, 108, 321	27±10, 14, 43	27±11, 15, 45	26±9, 14, 42
2z2d.A	171±54, 111, 333	28±10, 19, 58	25±8, 16, 47	29±10, 18, 57

REFERENCES

1. Volkamer A, Kuhn D, Grombacher T, Rippmann F, Rarey M. Combining global and local measures for structure-based druggability predictions. J Chem Inf Model. 2012;52(2):360–72. doi: 10.1021/ci200454v. PubMed PMID: 22148551.
