# Supplementary material for: A benchmark driven guide to binding site comparison: An exhaustive evaluation using tailor-made data sets (ProSPECCTs)
Source: PLoS Comput Biol. 2018 Nov 8;14(11):e1006483. doi: 10.1371/journal.pcbi.1006483 (PMC6224041; doi:10.1371/journal.pcbi.1006483)
Supplement: S7 Table — (PDF) [file pcbi.1006483.s008.pdf]

S7 Table. Overview of the data set of Kahraman and co-workers[1].

PDB ID	chain	ligand-id	PDB ID	chain	ligand-id
12as	A	AMP	1d0c	A	HEM
1amu	A	AMP	1d7c	A	HEM
1c0a	A	AMP	1dk0	A	HEM
1ct9	A	AMP	1eqg	A	HEM
1jp4	A	AMP	1ew0	A	HEM
1kht	B	AMP	1gwe	A	HEM
1qb8	A	AMP	1iqc	A	HEM
1tb7	B	AMP	1naz	A	HEM
8gpb	A	AMP	1np4	A	HEM
1a0i	A	ATP	1po5	A	HEM
1a49	A	ATP	1pp9	C	HEM
1ayl	A	ATP	1qhu	A	HEM
1b8a	A	ATP	1qla	C	HEM
1dv2	A	ATP	1qpa	A	HEM
1dy3	A	ATP	1sox	A	HEM
1e2q	A	ATP	2cpo	A	HEM
1e8x	A	ATP	1ej2	A	NAD
1esq	A	ATP	1hex	A	NAD
1gn8	A	ATP	1ib0	A	NAD
1kvk	A	ATP	1jq5	A	NAD
1o9t	B	ATP	1mew	A	NAD
1rdq	E	ATP	1mi3	A	NAD
1tid	A	ATP	1o04	A	NAD
1e3r	A	AND	1og3	A	NAD
1j99	A	AND	1qax	B	NAD
1fds	A	EST	1rlz	A	NAD
1lhu	A	EST	1s7g	B	NAD
1qkt	A	EST	1t2d	A	NAD
1cqx	A	FAD	1tox	A	NAD
1e8g	A	FAD	2a5f	B	NAD
1evi	A	FAD	2npx	A	NAD
1h69	A	FAD	1a6q	A	PO4
1hsk	A	FAD	1b8o	A	PO4
1jqi	A	FAD	1brw	A	PO4
1jr8	B	FAD	1cqj	B	PO4
1k87	A	FAD	1d1q	B	PO4
1pox	A	FAD	1dak	A	PO4
3grs	A	FAD	1e9g	A	PO4
1dnl	A	FMN	1ejd	B	PO4
1f5v	A	FMN	1euc	A	PO4
1ja1	A	FMN	1ew2	A	PO4
1mvl	A	FMN	1fbt	B	PO4
1p4c	A	FMN	1gyp	A	PO4
1p4m	A	FMN	1h6l	A	PO4
1bdg	A	GLC	1ho5	B	PO4
1cq1	A	GLC	1l5w	B	PO4
1k1w	A	GLC	1l7m	A	PO4
1nf5	B	GLC	1lby	A	PO4
2gbp	A	GLC	1lyv	A	PO4
			1qf5	A	PO4
			1tco	A	PO4

REFERENCES

1. Kahraman A, Morris RJ, Laskowski RA, Favia AD, Thornton JM. On the diversity of physicochemical environments experienced by identical ligands in binding pockets of unrelated proteins. Proteins. 2010;78(5):1120–36. doi: 10.1002/prot.22633. PubMed PMID: 19927322.
