# Supplementary material for: A benchmark driven guide to binding site comparison: An exhaustive evaluation using tailor-made data sets (ProSPECCTs)
Source: PLoS Comput Biol. 2018 Nov 8;14(11):e1006483. doi: 10.1371/journal.pcbi.1006483 (PMC6224041; doi:10.1371/journal.pcbi.1006483)
Supplement: S8 Table — (PDF) [file pcbi.1006483.s009.pdf]

**S8 Table.** Overview of the data set of Barelier *et al.*[1] and the RMSD values obtained by a least-squares fitting of corresponding ligand atoms of both complexes.

| PDB ID 1 | ligand 1 | cofactors 1 | PDB ID 2 | ligand 2 | cofactors 2 | pair class | ligand RMSD [Å] |
|---|---|---|---|---|---|---|---|
| 2rh1 | CAU.A.408 | - | 2q6h | CXX.A.801 | - | A | 3.755 |
| 2b50 | VCA.A.1001 | - | 4bvm | VCA.A.1133 | - | A | 2.815 |
| 1vyg | ACD.A.1134 | - | 1diy | ACD.A.700 | - | A | 2.808 |
| 1mv9 | HXA.A.200 | - | 1fdq | HXA.A.133 | - | A | 2.517 |
| 2ans | 2AN.A.201 | - | 1eyn | 2AN.A.550 | - | A | 1.526 |
| 2wd9 | IBP.A.1570 | - | 3p6h | IBP.A.133 | - | A | 1.442 |
| 4nyq | EIC.A.208 | - | 4ia6 | EIC.A.608 | - | A | 1.424 |
| 3hig | BRN.A.901 | - | 2gby | BRN.A.1110 | - | A | 1.360 |
| 2qre | AMZ.G.1001 | - | 1m9n | AMZ.A.1001 | - | A | 1.302 |
| 1eyn | 2AN.A.550 | - | 1ow4 | 2AN.B.1003 | - | A | 1.043 |
| 3cf9 | AGI.A.161 | - | 4hkk | AGI.A.1204 | - | A | 1.010 |
| 3fty | 3IP.A.710 | - | 1w7h | 3IP.A.1355 | - | A | 0.909 |
| 2ans | 2AN.A.201 | - | 1ow4 | 2AN.B.1003 | - | A | 0.866 |
| 2x8z | X8Z.A.1615 | - | 4dpr | X8Z.A.702 | - | A | 0.854 |
| 2wyd | DAO.B.1552 | - | 2ovd | DAO.A.190 | - | A | 0.849 |
| 3bra | AEF.A.394 | - | 4n7c | AEF.A.202 | - | A | 0.822 |
| 2g0l | FLN.A.114 | - | 4hki | FLN.A.1204 | - | A | 0.784 |
| 1e6q | NTZ.M.999 | - | 1nok | NTZ.A.998 | - | A | 0.266 |
| 2ph9 | GNT.A.301 | - | 1dx6 | GNT.A.1537 | PG4 | A | 0.234 |
| 1tx0 | PT1.A.283 | SO4 | 1br6 | PT1.A.301 | - | B | 2.107 |
| 1rf7 | DHF.A.161 | - | 1lcb | DHF.A.318 | TMP | B | 1.861 |
| 3hbf | MYC.A.901 | - | 2o63 | MYC.A.501 | IMD | B | 1.756 |
| 2hkk | ALE.A.300 | - | 3pah | XDE.A.700 | - | B | 1.730 |
| 1z9y | FUN.A.500 | - | 2xn5 | FUN.A.1356 | - | B | 1.676 |
| 4hcy | 0YN.A.401 | - | 4mhw | 0YN.A.201 | - | B | 1.641 |
| 1ve7 | 4NP.B.1701 | - | 2i6p | 4NP.A.401 | - | B | 1.503 |
| 2v6t | H2B.A.1104 | - | 2fbz | H2B.X.903 | HEM | B | 1.491 |
| 3hlg | LVA.A.500 | - | 1t02 | LVA.A.429 | - | B | 1.366 |
| 1pa9 | CSN.A.737 | - | 1e2s | CSN.P.701 | - | B | 1.231 |
| 2nsl | C2R.A.300 | - | 2gqs | C2R.A.242 | FMT | B | 1.201 |
| 3ln1 | CEL.A.682 | - | 1oq5 | CEL.A.701 | - | B | 1.182 |
| 3wl8 | OCA.A.402 | - | 2wg9 | OCA.A.301 | - | B | 1.143 |
| 2vyv | 1GP.D.1334 | NAD | 3ew5 | 1GP.B.11 | - | B | 1.048 |
| 2yyj | 4HP.A.550 | FAD | 2yaj | 4HP.A.1898 | - | B | 0.878 |
| 4kcx | 1QK.A.201 | - | 4kd1 | 1QK.A.302 | - | B | 0.870 |
| 3hbg | MTE.A.501 | - | 3etr | MTE.C.1326 | MOS | B | 0.796 |
| 3ebp | CPB.A.940 | - | 3blr | CPB.A.940 | - | B | 0.765 |
| 3eau | PDN.A.501 | NDP | 2q1v | PDN.A.248 | - | B | 0.751 |
| 4lzr | LOC.A.201 | - | 4o2b | LOC.B.503 | - | B | 0.673 |
| 1mxh | DHF.A.1278 | NAP | 1rf7 | DHF.A.161 | - | B | 0.667 |
| 1xql | 4AX.B.505 | - | 1pb9 | 4AX.A.901 | - | B | 0.593 |
| 1b66 | BIO.A.700 | - | 1sep | BIO.A.801 | NAP | B | 0.543 |
| 2oyf | IAC.A.1001 | - | 4hm0 | IAC.A.505 | - | B | 0.523 |
| 1eyq | NAR.A.501 | - | 2uxu | NAR.A.1211 | - | B | 0.496 |
| 1mxh | DHF.A.1278 | NAP | 1lcb | DHF.A.318 | TMP | B | 0.188 |
| 3dds | CFF.B.903 | - | 2a3b | CFF.A.1433 | - | B | 0.067 |
| 3ed0 | EMO.C.161 | - | 3bqc | EMO.A.400 | - | B | 0.055 |
| 4cus | ES1.A.2972 | - | 3kpu | ES1.A.290 | - | B | 0.035 |

**S8 Table *(continued)*.** Overview of the data set of Barelier *et al.*[1] and the RMSD values obtained by a least-squares fitting of corresponding ligand atoms of both complexes.

| PDB ID 1 | ligand 1 | cofactors 1 | PDB ID 2 | ligand 2 | cofactors 2 | pair class | ligand RMSD [Å] |
|---|---|---|---|---|---|---|---|
| 3b00 | 16A.A.300 | - | 3p73 | 16A.A.274 | - | C | 2.964 |
| 1qhy | CLM.A.999 | - | 4cla | CLM.A.221 | | C | 2.059 |
| 3tvl | 3PO.A.231 | - | 4odj | 3PO.A.501 | - | C | 1.738 |
| 1s2c | FLF.A.2001 | NAP | 2pix | FLF.A.2386 | - | C | 1.349 |
| 2uy4 | AZM.A.1311 | - | 3hs4 | AZM.A.701 | GOL, ZN | C | 1.003 |
| 2otf | 2TN.A.201 | - | 2nuv | 2TN.A.2001 | - | C | 0.959 |
| 2o73 | 2AL.B.1001 | - | 2fxl | 2AL.A.302 | - | C | 0.955 |
| 3t4k | EMU.A.400 | - | 4jhi | EMU.A.202 | | C | 0.923 |
| 3gcl | AIN.A.609 | - | 1oxr | AIN.A.141 | - | C | 0.613 |
| 2p1q | IAC.B.901 | - | 4hm0 | IAC.A.505 | - | C | 0.539 |
| 2qvd | BER.A.1811 | - | 3d6y | BER.A.964 | | C | 0.536 |
| 2p1q | IAC.B.901 | - | 2oyf | IAC.A.1001 | - | C | 0.433 |
| 2fu7 | PHN.A.410 | - | 2lig | PHN.A.390 | - | C | 0.127 |
| 1s4m | LUM.A.294 | - | 1he5 | LUM.A.1207 | NAP | C | 0.080 |

REFERENCES


1. Barelier S, Sterling T, O'Meara MJ, Shoichet BK. The recognition of identical ligands by unrelated proteins. ACS Chem Biol. 2015;10(12):2772–84. doi: 10.1021/acschembio.5b00683. PubMed PMID: 26421501.
