# Supplementary material for: A benchmark driven guide to binding site comparison: An exhaustive evaluation using tailor-made data sets (ProSPECCTs)
Source: PLoS Comput Biol. 2018 Nov 8;14(11):e1006483. doi: 10.1371/journal.pcbi.1006483 (PMC6224041; doi:10.1371/journal.pcbi.1006483)
Supplement: S9 Table — Structures which are also in the reduced data set 1.2 are highlighted in bold characters. (PDF) [file pcbi.1006483.s010.pdf]

S9 Table. Data set of structures with identical sequences (data set 1). Structures which are also in the reduced data set 1.2 are highlighted in bold characters.

PDB ID	chain	ligand-id	PDB ID	chain	ligand-id	PDB ID	chain	ligand-id
thrombin (*Homo sapiens*)			carbonic anhydrase II (*Homo sapiens*)			heat shock protein 90 (*Homo sapiens*)		
**2zda**	**H**	**32U**	1bnq	A	AL4	**1uye**	**A**	**PU9**
**2zc9**	**H**	**22U**	1bnw	A	TPD	**1uyd**	**A**	**PU8**
3qwc	H	98P	1bnv	A	AL7	1uyg	A	PU2
**3sv2**	**H**	**P05**	1bnu	A	AL3	1uyf	A	PU1
**3si3**	**H**	**B03**	1bnn	A	AL1	1uyc	A	PU7
**3si4**	**H**	**B04**	1bnm	A	AL8	1uyh	A	PU0
1ypl	H	RA8	3oys	A	OYS	2xhr	A	C0P
1ypm	H	RA4	1i91	A	INQ	2qfo	A	A51
1ype	H	UIP	1okl	A	MNS	2xk2	A	ADP
2uuj	B	896	1bn1	A	AL5	2xht	A	C0Y
**2zff**	**H**	**53U**	4bf6	A	X0Q	2h55	A	DZ8
3rmm	H	M32	3k2f	A	NKX	4nh8	A	2LC
2znk	H	31U	3m98	A	E02	2uwd	A	2GG
2zfp	H	19U	**3m96**	**A**	**E38**	3wha	B	WHA
2zfq	H	45U	3m04	A	BE9	3vha	A	VHA
2fes	H	3SP	3bet	A	CTF	**1uy7**	**A**	**PU4**
2feq	H	34P	3n0n	A	P9B	**1uy6**	**A**	**PU3**
**3p17**	**H**	**99P**	**3s9t**	**A**	**E49**	1uyk	A	PUX
3qx5	H	02P	4dz9	A	ID4	4efu	A	EFU
3t5f	H	M34	2h15	A	B19	1uy9	A	PU6
2anm	H	CDO	3d9z	A	D9Z	3r4p	A	FU7
1d6w	A	00R	2hl4	A	BO1	3r4o	B	FU3
3u98	H	BJA	4fpt	A	0VZ	3r4n	B	FU5
2ank	H	N12	3p5l	A	IT5	1osf	A	KOS
**2zf0**	**H**	**51U**	2aw1	A	COX	3inx	A	JZC
2a2x	H	NA9	1cim	A	PTS	3inw	A	JZB
1kts	B	C24	2eu2	A	5DS	3hz5	A	Z64
1ktt	B	C02	4mty	A	SBW	2byh	A	2D7
1o0d	H	163	1xq0	A	4TR	3mnr	P	SD1
3da9	B	44U	1a42	A	BZU	2xab	A	VHD
1nzq	H	162	2wd3	A	MS4	3bmy	A	CXZ
2cf9	H	348	3mhl	A	J71	2vci	A	2GJ
4e7r	G	0NW	3mhm	A	J75	2vcj	A	2EQ
3rm0	H	S54	3mhc	A	ARZ	2yi7	A	BZ8
3rm2	H	S00	2hd6	A	BOS	3rlr	A	3RR
3rlw	H	S28	3dd8	A	2C7	3b28	A	B2X
3rly	H	S29	3mzc	A	S6I	3b25	A	B2K
2jh0	D	701	3ffp	X	LC1	2fwy	A	H64
2jh6	D	894	3f4x	A	KLT	2fwz	A	H71
3rml	H	M31	3l14	A	I7B	4b7p	A	9UN
3u9a	H	S33	3t84	A	SG6	3o0i	A	P54
3rmn	H	M41	4kni	A	E1E	2qg0	A	A94
3rmo	H	S04	4knj	A	E1F	4bqg	A	50Q
1d9i	A	00P	3vbd	A	0FZ	4egk	A	RDC
2v3o	H	I26	1ze8	A	PIU	3t1k	A	ANP
1c4u	2	IH1	1kwq	A	SG1	4bqj	A	XKL
1c4v	2	IH2	3r17	B	5UM	4jql	A	VJ6
1ypk	H	CCR	3m2x	A	BEX	2wi6	A	ZZ6
3shc	H	B01	1z9y	A	FUN	2wi4	A	ZZ4
3sha	H	P97	1i8z	A	INL	3qdd	A	94M
1d4p	B	BPP	4e3f	A	GRE	2byi	A	2DD
2ziq	H	26U	3sax	A	E50	1uy8	A	PU5

S9 Table *(continued)*. Data set of structures with identical sequences (data set 1). Structures which are also in the reduced data set 1.2 are highlighted in bold characters.

PDB ID	chain	ligand-id	PDB ID	chain	ligand-id	PDB ID	chain	ligand-id
thrombin (*Homo sapiens*)			carbonic anhydrase II (*Homo sapiens*)			heat shock protein 90 (*Homo sapiens*)		
3biu	H	10U	1ttm	A	667	3t10	A	ACP
2uuk	B	897	3c7p	A	POF	3k98	B	1RC
2gde	H	SN3	2hoc	A	1CN	3d0b	A	SNX
**3qto**	**H**	**10P**	**3m67**	**A**	**E36**	3k97	A	4CD
**3qtv**	**H**	**06P**	3sbi	A	E90			
1oyt	H	FSN	3igp	A	DT7			
1d3d	B	BZT	4itp	A	1GD			
2zi2	H	24U	3po6	A	DT9			
1d3p	B	BT3	3b4f	A	TUO			
1g30	B	T87						
1g32	B	R11						
dihydrofolate reductase (*Homo sapiens*)			isopenicillin N synthetase (*Homo sapiens*)			macrophage metalloelastase (*Homo sapiens*)		
3nu0	A	3TU	2bu9	A	HFV	**1rmz**	**A**	**NGH**
3ghw	A	GHW	**2y6f**	**A**	**M9F**	3lk8	A	Z79
3ntz	A	3TZ	**1qiq**	**A**	**ACC**	**3nx7**	**A**	**NHK**
1mvs	A	DTM	**1odm**	**A**	**ASV**	3ts4	A	EEG
1pd9	A	CO4	1odn	A	APV	3rtt	A	KLH
4keb	A	1QZ	**1bk0**	**A**	**ACV**	4guy	A	KLJ
1yho	A	TRR	1w3x	A	W2X	4h76	A	10B
3nxt	A	D2E	**1w05**	**A**	**W05**	3n2v	A	JT5
4kd7	A	9DR	1w3v	A	MDZ	4gr0	A	R4B
1hfr	A	MOT	**2vbp**	**A**	**VB1**	4efs	A	E37
1u72	A	MTX	3zku	A	HCV	**3f15**	**A**	**HS1**
**4m6l**	**A**	**21V**	3zky	A	WT4	3f16	A	HS3
1s3w	A	TQT	3zoi	A	M2W	3f17	A	HS4
1s3v	A	TQD	**2y60**	**A**	**M8F**	3f18	A	HS5
4ddr	A	MMV	1qjf	A	ACS	2hu6	A	37A
**2dhf**	**A**	**DZF**	1hb4	A	SCV	3ehx	A	BDL
3nxv	A	D2F	2ivi	B	ACW	3ehy	A	TBL
3nxy	A	D2H	2ivj	A	BCV	3rts	A	KLG
1drf	A	FOL	1hb1	A	OCV	3tsk	A	QEG
4g95	A	OAG	2vcm	A	M11			
1ohk	A	COP	2vbd	A	V10			
1kmv	A	LII	**1uzw**	**A**	**CDH**			
3s7a	A	684	2vau	A	V20			
4kbn	A	25U	4bb3	A	KKA			

S9 Table *(continued)*. Data set of structures with identical sequences (data set 1). Structures which are also in the reduced data set 1.2 are highlighted in bold characters.

PDB ID	chain	ligand-id	PDB ID	chain	ligand-id	PDB ID	chain	ligand-id
leukotriene A4 hydrolase (*Homo sapiens*)			D-alanyl-D-alanine carboxypeptidase (*Streptomyces* sp. R61)			bromodomain-containing protein 4 (*Homo sapiens*)		
3u9w	A	28P	1pwl	A	BFI	**4j0r**	**A**	**1H2**
3b7u	X	KEL	3g5e	A	Q74	**4j0s**	**A**	**1H3**
3b7r	L	BIR	2qxw	A	LDT	4men	A	25K
3chs	A	4BU	2ikh	A	LIT	4meo	A	25V
**3chp**	**A**	**4BO**	**1x97**	**A**	**FIR**	4hxp	A	1A5
2r59	A	PH0	3p2v	A	DOY	4hxr	A	1A4
**3cho**	**A**	**4BG**	3t42	A	3T4	4a9l	A	P9L
3fh8	A	27P	2nvd	A	ITB	4gpj	A	0Q1
3fh7	A	25P	2nvc	A	ITA	4c67	A	L5S
3fh5	A	24P	3u2c	A	SUZ	3u5l	A	08K
**3chq**	**A**	**4BQ**	3dn5	A	53N	3u5j	A	08H
3ful	A	52D	2ikg	A	BTO	4hbx	A	14X
3fum	A	80A	1z3n	A	3NA	4hbv	A	15E
3fun	A	798	**1pwm**	**A**	**FID**			
3fui	A	812	1z89	A	62P			
3fuk	A	58Z	1t41	A	ID5			
3fuf	A	BES	4gca	A	2X9			
tankyrase-2 (*Homo sapiens*)			angiotensin-converting enzyme (*Drosophila melanogaster*)			pantothenate kinase (*Mycobacterium tuberculosis*)		
3u9y	A	09L	4ca7	A	3EF	**2zsa**	**A**	**ADP**
3p0n	C	BPU	2x95	A	X95	2zsd	A	COA
4bud	A	29F	**2x94**	**A**	**X94**	4bft	B	ZVT
3kr8	C	XAV	2x97	A	RX4	4bfv	B	ZVV
4bux	A	F35	2x96	A	RX3	4bfw	B	ZVW
**4buu**	**A**	**F38**	2x91	A	LPR	4bfx	A	ZVX
**4but**	**A**	**31F**	2x90	A	EAL	4bfy	B	ZVY
4buw	A	F33	**2x93**	**A**	**X93**	4bfz	A	ZVZ
4bus	A	32F	**2x92**	**A**	**X92**	**3af2**	**A**	**ACP**
3ua9	B	IWR	2xhm	A	K26	**3af3**	**A**	**GCP**
4j3m	B	AJ8						
4buy	A	F37						
