# Supplementary material for: A benchmark driven guide to binding site comparison: An exhaustive evaluation using tailor-made data sets (ProSPECCTs)
Source: PLoS Comput Biol. 2018 Nov 8;14(11):e1006483. doi: 10.1371/journal.pcbi.1006483 (PMC6224041; doi:10.1371/journal.pcbi.1006483)
Supplement: S10 Table — The column ID holds the first two letters of the model names in our downloadable data set. (PDF) [file pcbi.1006483.s011.pdf]

S10 Table. Overview of the data set of NMR ensemble structures (data set 2). The column ID holds the first two letters of the model names in our downloadable data set.

PDB ID.chain	ID	ligand-id	conformers	protein
1cz2.A	cz	E2P	12	nonspecific lipid transfer protein (*Triticum aestivum*)
1diu.A	di	BDM	18	dihydrofolate reductase (*Lactobacillus casei*)
1eio.A	ei	GCH	5	ileal lipid binding protein (*Sus scrofa*)
1j5i.A	j5	NCZ	44	neocarzinostatin (*Streptomyces carzinostaticus*)
1kgl.A	kg	RTL	20	cellular retinol-binding protein type I (*Rattus norvegicus*)
1mux.A	mu	WW7	30	calmodulin (*Xenopus laevis*)
1t84.A	t8	WSK	20	Wiskott-Aldrich syndrome protein (*Homo sapiens*)
1tvc.A	tv	FDA	10	methane monooxygenase component C (*Methylococcus capsulatus*)
1yho.A	yh	TRR	25	dihydrofolate reductase (*Homo sapiens*)
2jt2.A	jt	C90	25	UDP-3-O-[3-hydroxymyristoyl]-N-acetylglucosamine deacetylase (*Aquifex aeolicus*)
2k31.A	k3	35G	20	cGMP-specific 3’,5’-cyclic phosphodiesterase (*Mus musculus*)
2k5t.A	k5	COA	20	PanD maturation factor (*Escherichia coli*)
2l0x.A	l0	GDP	20	GTP-binding protein Rheb (*Rattus norvegicus*)
2l2s.A	l2	L2S	20	peptidyl-prolyl cis-trans isomerase (*Burkholderia pseudomallei*)
2l8r.A	l8	APR	20	O-acetyl-ADP-ribose deacetylase 1 (*Homo sapiens*)
2lzg.A	lz	13Q	5	E3 ubiquitin-protein ligase Mdm2 (*Homo sapiens*)
2z2d.A	z2	HSI	15	macrophage metalloelastase (*Homo sapiens*)
