# Supplementary material for: A benchmark driven guide to binding site comparison: An exhaustive evaluation using tailor-made data sets (ProSPECCTs)
Source: PLoS Comput Biol. 2018 Nov 8;14(11):e1006483. doi: 10.1371/journal.pcbi.1006483 (PMC6224041; doi:10.1371/journal.pcbi.1006483)
Supplement: S11 Table — (PDF) [file pcbi.1006483.s012.pdf]

S11 Table. Query structure matches used for the data set of successful examples (data set 7).

PDB ID	chain	ligand (name.number.chain)	reference
2ejr	A	F2N.1.A	[1]
2bxr	A	FAD.600.A	
1gos	A	FAD.600.A	
2f6d	A	ACR.995.A	[2]
1k1y	A	ACR.660.A	
3lj3	A	WYE.1109.A	[2]
3ma3	A	01I.313.A	
1rx7	A	FOL.161.A	[2]
3bhr	A	THG.266.A	
1dg5	A	NDP.200.A	[2]
3dl6	A	NDP.606.A	
6cox	A	S58.701.A	[3]
1bn4	A	AL9.555.A	
4tzk	A	641.501.A	[4]
2cl5	A	BIE.1218.A	
1eve	A	E20.2001.A	[5]
3cjg	A	KIM.1167.A	
2oht	A	IP6.601.A	[6]
2ito	A	IRE.2020.A	
1ohr	A	1UN.201.A	[7]
2j6m	A	AEE.2021.A	
3c0z	B	SHH.301.B	[8]
5hfa	A	FP1.607.A	
3a99	A	AGS.800.A	[9]
1aux	A	ANP.501.A	
1m17	A	AQ4.999.A	[10]
1y57	A	MPZ.600.A	
1qpe	A	PP2.1904.A	
2f4j	A	VX6.514.A	
2rew	A	REW.469.A	[11]
3rr3	A	FLR.700.A	
1xpc	A	AIT.600.A	[12]
2agv	A	TG1.1003.A	
1i7d	A	DG.702.B	[13]
5dlj	D	DT.35.H	
2mjp	A	ANP.500.A	[14]
1a49	A	ATP.535.A	
4kam	A	SO4.501.A	[15]
1e5f	A	PLP.405.A	
1xel	A	NAD.340.A	[16]
1e6w	A	NAD.301.A	
1gal	A	FAD.600.A	
1uk4	B	chain H (pentapeptide)	[16]
1cqq	A	AG7.501.A	
1b0u	A	ATP.301.A	[17]
1jb1	A	PO4.400.A	
1gvr	A	FMN.401.A	[18]
1z41	A	FMN.1500.A	
2hs6	A	FMN.401.A	

REFERENCES

1. Willmann D, Lim S, Wetzel S, Metzger E, Jandausch A, Wilk W, et al. Impairment of prostate cancer cell growth by a selective and reversible lysine-specific demethylase 1 inhibitor. Int J Cancer. 2012;131(11):2704–9. doi: 10.1002/ijc.27555. PubMed PMID: 22447389.
2. Haupt VJ, Daminelli S, Schroeder M. Drug promiscuity in PDB: Protein binding site similarity is key. PLoS One. 2013;8(6):e65894. doi: 10.1371/journal.pone.0065894. PubMed PMID: 23805191.
3. Weber A, Casini A, Heine A, Kuhn D, Supuran CT, Scozzafava A, et al. Unexpected nanomolar inhibition of carbonic anhydrase by COX-2-selective celecoxib: new pharmacological opportunities due to related binding site recognition. J Med Chem. 2004;47(3):550–7. doi: 10.1021/jm030912m. PubMed PMID: 14736236.
4. Kinnings SL, Liu N, Buchmeier N, Tonge PJ, Xie L, Bourne PE. Drug discovery using chemical systems biology: repositioning the safe medicine Comtan to treat multi-drug and extensively drug resistant tuberculosis. PLoS Comput Biol. 2009;5(7):e1000423. doi: 10.1371/journal.pcbi.1000423. PubMed PMID: 19578428.
5. Yang Y, Li G, Zhao D, Yu H, Zheng X, Peng X, et al. Computational discovery and experimental verification of tyrosine kinase inhibitor pazopanib for the reversal of memory and cognitive deficits in rat model neurodegeneration. Chem. Sci. 2015;6(5):2812–21. doi: 10.1039/c4sc03416c.
6. Niu M, Hu J, Wu S, Zhang X, Xu H, Zhang Y, et al. Structural bioinformatics-based identification of EGFR inhibitor gefitinib as a putative lead compound for BACE. Chem. Biol. Drug Des. 2014;83(1):81–8. doi: 10.1111/cbdd.12200.
7. Xie L, Evangelidis T, Xie L, Bourne PE. Drug discovery using chemical systems biology: weak inhibition of multiple kinases may contribute to the anti-cancer effect of nelfinavir. PLoS Comput Biol. 2011;7(4):e1002037. doi: 10.1371/journal.pcbi.1002037. PubMed PMID: 21552547.
8. Yang L, Chen J, Shi L, Hudock MP, Wang K, He L. Identifying unexpected therapeutic targets via chemical-protein interactome. PLoS One. 2010;5(3):e9568. doi: 10.1371/journal.pone.0009568. PubMed PMID: 20221449.
9. Defranchi E, Schalon C, Messa M, Onofri F, Benfenati F, Rognan D. Binding of protein kinase inhibitors to synapsin I inferred from pair-wise binding site similarity measurements. PLoS One. 2010;5(8):e12214. doi: 10.1371/journal.pone.0012214. PubMed PMID: 20808948.
10. Kinnings SL, Jackson RM. Binding site similarity analysis for the functional classification of the protein kinase family. J Chem Inf Model. 2009;49(2):318–29. doi: 10.1021/ci800289y. PubMed PMID: 19434833.
11. Cleves AE, Jain AN. Chemical and protein structural basis for biological crosstalk between PPARalpha and COX enzymes. J Comput Aided Mol Des. 2015;29(2):101–12. doi: 10.1007/s10822-014-9815-2. PubMed PMID: 25428568.
12. Xie L, Wang J, Bourne PE. In silico elucidation of the molecular mechanism defining the adverse effect of selective estrogen receptor modulators. PLoS Comput Biol. 2007;3(11):e217. doi: 10.1371/journal.pcbi.0030217. PubMed PMID: 18052534.
13. Babu M, Beloglazova N, Flick R, Graham C, Skarina T, Nocek B, et al. A dual function of the CRISPR-Cas system in bacterial antivirus immunity and DNA repair. Mol

Microbiol. 2011;79(2):484–502. doi: 10.1111/j.1365-2958.2010.07465.x. PubMed PMID: 21219465.
14. Kinoshita K, Nakamura H. Identification of protein biochemical functions by similarity search using the molecular surface database eF-site. Protein Sci. 2003;12(8):1589–95. doi: 10.1110/ps.0368703. PubMed PMID: 12876308.
15. Anand P, Sankaran S, Mukherjee S, Yeturu K, Laskowski R, Bhardwaj A, et al. Structural annotation of Mycobacterium tuberculosis proteome. PLoS One. 2011;6(10):e27044. doi: 10.1371/journal.pone.0027044. PubMed PMID: 22073123.
16. Kuhn D, Weskamp N, Schmitt S, Hüllermeier E, Klebe G. From the similarity analysis of protein cavities to the functional classification of protein families using cavbase. J Mol Biol. 2006;359(4):1023–44. doi: 10.1016/j.jmb.2006.04.024. PubMed PMID: 16697007.
17. Ausiello G, Peluso D, Via A, Helmer-Citterich M. Local comparison of protein structures highlights cases of convergent evolution in analogous functional sites. BMC Bioinformatics. 2007;8 Suppl 1:S24. doi: 10.1186/1471-2105-8-S1-S24. PubMed PMID: 17430569.
18. Tseng YY, Li W-H. Classification of protein functional surfaces using structural characteristics. Proc Natl Acad Sci U S A. 2012;109(4):1170–5. doi: 10.1073/pnas.1119684109. PubMed PMID: 22238424.
