# Supplementary material for: A benchmark driven guide to binding site comparison: An exhaustive evaluation using tailor-made data sets (ProSPECCTs)
Source: PLoS Comput Biol. 2018 Nov 8;14(11):e1006483. doi: 10.1371/journal.pcbi.1006483 (PMC6224041; doi:10.1371/journal.pcbi.1006483)
Supplement: S12 Table — The thresholds were defined separately for each data set based on the corresponding ROC curves. Means and standard deviations for the cut-off values for all data sets are given together with the corresponding score and the score range (whenever applicable). The distance measures of RAPMAD and SiteAlign were transformed to the corresponding similarities. (PDF) [file pcbi.1006483.s013.pdf]

S12 Table. Cut-off values for all comparison methods set defined using the Youden's J statistic[1]. The thresholds were defined separately for each data set based on the corresponding ROC curves. Means and standard deviations for the cut-off values for all data sets are given together with the corresponding score and the score range. The distance measures of RAPMAD and SiteAlign were transformed to the corresponding similarities.

method	data set 1	data set 1.2	data set 2	data set 3	data set 4	data set 5	data set 5.2
Cavbase	15.85	21.75	8.52	35.57	34.15	5.70	5.70
FuzCav (PDB)	0.21	0.28	0.22	0.27	0.36	0.21	0.21
FuzCav	0.21	0.28	0.22	0.27	0.36	0.21	0.22
Grim (PDB)	0.57	0.59	0.57	0.57	0.57	0.57	0.56
Grim	0.57	0.59	0.58	0.56	0.56	0.56	0.56
IsoMIF	0.49	0.62	0.50	0.60	0.62	0.37	0.35
KRIPO	0.50	0.52	0.51	0.56	0.56	0.46	0.44
PocketMatch	0.16	0.17	0.29	0.42	0.40	0.11	0.11
ProBiS	8.03	7.96	8.66	11.42	11.34	0.01	0.01
RAPMAD	0.89	0.80	0.75	0.74	0.73	0.90	0.95
VolSite/Shaper (PDB)	0.89	0.95	0.88	1.20	1.15	0.75	0.70
VolSite/Shaper	0.89	0.95	0.89	1.20	1.15	0.73	0.71
Shaper (PDB)	0.89	0.97	0.90	1.17	1.12	0.71	0.71
Shaper	0.89	0.97	0.90	1.16	1.12	0.74	0.74
SiteAlign	0.85	0.85	0.85	0.97	0.97	0.76	0.77
SiteEngine	875.25	1,444.60	244.00	1,431.71	1,516.61	417.60	417.60
SiteHopper	0.95	1.57	1.11	2.23	2.21	0.64	0.65
SMAP	69.18	98.17	92.21	184.71	218.71	44.83	51.47
TIFP (PDB)	0.20	0.38	0.14	0.10	0.05	0.08	0.03
TIFP	0.23	0.20	0.20	0.05	0.03	0.13	0.09
TM-align	0.49	0.64	0.67	0.85	0.85	0.25	0.25

S12 Table *(continued)*. Cut-off values for all comparison methods set defined using the Youden's J statistic[1]. The thresholds were defined separately for each data set based on the corresponding ROC curves. Means and standard deviations for the cut-off values for all data sets are given together with the corresponding score and the score range. The distance measures of RAPMAD and SiteAlign were transformed to the corresponding similarities.

method	data set 6	data set 6.2	data set 7	mean	standard deviation	score	score range
Cavbase	1.34	1.34	8.33	13.83	12.71	similarity score	n.d.
FuzCav (PDB)	0.10	0.10	0.20	0.22	0.08	similarity score	[0,1]
FuzCav	0.10	0.10	0.20	0.22	0.08	similarity score	[0,1]
Grim (PDB)	0.55	0.55	0.59	0.57	0.01	Grscore	n.d.
Grim	0.27	0.55	0.58	0.54	0.10	Grscore	n.d.
IsoMIF	0.36	0.36	0.46	0.47	0.11	tani	[0,1]
KRIPO	0.44	0.44	0.56	0.50	0.05	similarity score	[0,1]
PocketMatch	0.03	0.03	0.26	0.20	0.14	$PMScore_{max}$	[0,1]
ProBiS	2.70	2.70	2.98	5.58	4.39	Alignment Score	n.d.
RAPMAD	0.81	0.81	0.97	0.83	0.09	1-distance score	[0,1]
VolSite/Shaper (PDB)	0.80	0.22	0.98	0.85	0.27	Tanimoto combo	[0,2]
VolSite/Shaper	0.71	0.70	0.99	0.89	0.18	Tanimoto combo	[0,2]
Shaper (PDB)	0.40	0.38	0.94	0.82	0.27	Tanimoto combo	[0,2]
Shaper	0.40	0.38	0.98	0.83	0.27	Tanimoto combo	[0,2]
SiteAlign	0.69	0.67	0.80	0.82	0.10	1-d3	[0,1]
SiteEngine	164.64	164.64	1,063.70	774.03	558.32	Curvature Score	n.d.
SiteHopper	0.65	0.65	0.87	1.15	0.63	PatchScore	[0,4]
SMAP	38.27	38.27	67.61	90.34	62.73	RawScore	n.d.
TIFP (PDB)	0.11	0.07	0.35	0.15	0.12	Soergel	[0,1]
TIFP	0.15	0.15	0.17	0.14	0.07	Soergel	[0,1]
TM-align	0.20	0.20	0.38	0.48	0.26	TM-score	[0,1]

REFERENCES

1. Youden WJ. Index for rating diagnostic tests. Cancer. 1950;3(1):32–5. doi: 10.1002/1097-0142(1950)3:1<32:AID-CNCR2820030106>3.0.CO;2-3.
