# Supplementary material for: A benchmark driven guide to binding site comparison: An exhaustive evaluation using tailor-made data sets (ProSPECCTs)
Source: PLoS Comput Biol. 2018 Nov 8;14(11):e1006483. doi: 10.1371/journal.pcbi.1006483 (PMC6224041; doi:10.1371/journal.pcbi.1006483)
Supplement: S13 Table — With respect to the applicability toward predicted sites, a plus in brackets means that the predicted binding site has to be given with the corresponding coordinates of a binding site prediction as “artificial” ligand, a simple plus denotes tools that offer a way of binding site prediction. With respect to run time evaluation, “+”, “/”, “-” denote comparison algorithms that require several ns, µs, or s per comparison, respectively. With respect to the scoring, a “+” was assigned to those tools where the intervals of upper and lower whiskers of active and inactive pairs do not overlap. A “/” denotes tools where the upper and lower quartile for the pairs do not overlap. With respect to other factors, tools that were clearly outperformed by many other tools were assigned a “-”. (PDF) [file pcbi.1006483.s014.pdf]

S13 Table. Different criteria of importance for the choice of a suitable binding site comparison method. A plus in brackets means that the predicted binding site has to be given with the corresponding coordinates of a binding site prediction as “artificial” ligand, a simple plus denotes tools that offer a way of binding site prediction. With respect to run time evaluation, “+”, “/”, and “-” denote comparison algorithms that require several ns, µs, or s per comparison, respectively. With respect to the scoring, a “+” was assigned to those tools where the intervals of upper and lower whiskers of active and inactive pairs did not overlap. A “/” denotes tools where the upper and lower quartile for the pairs do not overlap. With respect to other factors, tools that were clearly outperformed by many other tools were assigned a “-”.

method	BS preparation (ease)	BS preparation (completeness)	applicability toward predicted binding sites	run time	binding site definition	binding site definition (ranking)	binding site flexibility	binding site properties (ranking)	lifelike data set	visualization
reference		all data sets (S10 Fig)		Table 6	data set 1	data set 1 (S3-S5 Fig, S4 Table)	data set 2	data sets 3 and 4	data set 7	
Cavbase[1,2]	+	-	+	-	+	+	+	+	+	+
FuzCav[3]	/	+	+	+	+	/	+	+	+	-
Grim[4]	/	-	-	/	-	-	+	-	-	+
IsoMIF[5]	+	+	+	/	-	-	-	-	+	+
KRIPO[6]	+	+	-	+	-	/	+	+	+	+
PocketMatch[7]	-	-	(+)	+	-	/	+	-	+	-
ProBiS[8]	+	+	(+)	+	+	+	+	-	+	+
RAPMAD[9]	+	-	+	+	-	-	-	+	-	-
VolSite/ Shaper[10]	/	-	+	/	+	/	-	+	+	+
SiteAlign[11]	-	+	(+)	-	+	+	+	+	+	+
SiteEngine[12]	+	+	-	-	+	/	+	+	+	+
SiteHopper[13]	+	/	(+)[14]	/	+	+	+	+	+	+
SMAP[15]	+	+	(+)	-	+	+	+	+	+	+
TIFP[4]	/	-	-	/	-	-	+	-	-	-
TM-align[16]	-	+	(+)	/	+	+	+	n.d.	+	+

REFERENCES

1. Schmitt S, Hendlich M, Klebe G. From structure to function: A new approach to detect functional similarity among proteins independent from sequence and fold homology. Angew. Chem. Int. Ed. 2001;40(17):3141–4. doi: 10.1002/1521-3773(20010903)40:17<3141:AID-ANIE3141>3.0.CO;2-X.
2. Schmitt S, Kuhn D, Klebe G. A new method to detect related function among proteins independent of sequence and fold homology. J Mol Biol. 2002;323(2):387–406. PubMed PMID: 12381328.
3. Weill N, Rognan D. Alignment-free ultra-high-throughput comparison of druggable protein-ligand binding sites. J Chem Inf Model. 2010;50(1):123–35. doi: 10.1021/ci900349y. PubMed PMID: 20058856.
4. Desaphy J, Raimbaud E, Ducrot P, Rognan D. Encoding protein-ligand interaction patterns in fingerprints and graphs. J Chem Inf Model. 2013;53(3):623–37. doi: 10.1021/ci300566n. PubMed PMID: 23432543.
5. Chartier M, Najmanovich R. Detection of binding site molecular interaction field similarities. J Chem Inf Model. 2015;55(8):1600–15. doi: 10.1021/acs.jcim.5b00333. PubMed PMID: 26158641.
6. Wood DJ, Vlieg J de, Wagener M, Ritschel T. Pharmacophore fingerprint-based approach to binding site subpocket similarity and its application to bioisostere replacement. J Chem Inf Model. 2012;52(8):2031–43. doi: 10.1021/ci3000776. PubMed PMID: 22830492.
7. Yeturu K, Chandra N. PocketMatch: a new algorithm to compare binding sites in protein structures. BMC Bioinformatics. 2008;9:543. doi: 10.1186/1471-2105-9-543. PubMed PMID: 19091072.
8. Konc J, Janežič D. ProBiS algorithm for detection of structurally similar protein binding sites by local structural alignment. Bioinformatics. 2010;26(9):1160–8. doi: 10.1093/bioinformatics/btq100. PubMed PMID: 20305268.
9. Krotzky T, Grunwald C, Egerland U, Klebe G. Large-scale mining for similar protein binding pockets: with RAPMAD retrieval on the fly becomes real. J Chem Inf Model. 2015;55(1):165–79. doi: 10.1021/ci5005898. PubMed PMID: 25474400.
10. Desaphy J, Azdimousa K, Kellenberger E, Rognan D. Comparison and druggability prediction of protein-ligand binding sites from pharmacophore-annotated cavity shapes. J Chem Inf Model. 2012;52(8):2287–99. doi: 10.1021/ci300184x. PubMed PMID: 22834646.
11. Schalon C, Surgand J-S, Kellenberger E, Rognan D. A simple and fuzzy method to align and compare druggable ligand-binding sites. Proteins. 2008;71(4):1755–78. doi: 10.1002/prot.21858. PubMed PMID: 18175308.
12. Shulman-Peleg A, Nussinov R, Wolfson HJ. SiteEngines: recognition and comparison of binding sites and protein-protein interfaces. Nucleic Acids Res. 2005;33(Web Server issue):W337-41. doi: 10.1093/nar/gki482. PubMed PMID: 15980484.
13. Batista J, Hawkins PCD, Tolbert R, Geballe MT. SiteHopper - a unique tool for binding site comparison. J Cheminform. 2014;6(Suppl 1):P57. doi: 10.1186/1758-2946-6-S1-P57.
14. Meyers J, Brown N, Blagg J. Mapping the 3D structures of small molecule binding sites. J Cheminform. 2016;8(1):235. doi: 10.1186/s13321-016-0180-0.

15. Xie L, Xie L, Bourne PE. A unified statistical model to support local sequence order independent similarity searching for ligand-binding sites and its application to genome-based drug discovery. Bioinformatics. 2009;25(12):i305-12. doi: 10.1093/bioinformatics/btp220. PubMed PMID: 19478004.
16. Zhang Y, Skolnick J. TM-align: a protein structure alignment algorithm based on the TM-score. Nucleic Acids Res. 2005;33(7):2302–9. doi: 10.1093/nar/gki524. PubMed PMID: 15849316.
