# Supplementary material for: A benchmark driven guide to binding site comparison: An exhaustive evaluation using tailor-made data sets (ProSPECCTs)
Source: PLoS Comput Biol. 2018 Nov 8;14(11):e1006483. doi: 10.1371/journal.pcbi.1006483 (PMC6224041; doi:10.1371/journal.pcbi.1006483)
Supplement: S18 Table — (PDF) [file pcbi.1006483.s019.pdf]

S18 Table. AUC and EFs of different binding site comparison methods for data set 2.

method	AUC	$EF_{0.1\%}$	$EF_{0.5\%}$	$EF_{1\%}$	$EF_{2\%}$	$EF_{3\%}$	$EF_{4\%}$	$EF_{5\%}$
Cavbase	0.87	13.97	14.00	14.00	14.00	13.99	13.14	12.26
FuzCav	0.99	13.97	14.00	14.00	14.00	14.00	14.00	13.96
FuzCav (PDB)	0.98	13.97	14.00	14.00	14.00	14.00	13.99	13.94
Grim	0.92	13.97	14.00	14.00	14.00	13.99	13.83	12.58
Grim (PDB)	0.85	13.97	13.82	13.91	13.51	13.36	12.55	11.26
IsoMIF	0.70	13.97	13.92	13.90	13.28	11.81	10.24	8.92
KRIPO	0.96	13.97	14.00	14.00	14.00	13.94	13.61	12.95
PocketMatch	0.96	13.97	14.00	14.00	14.00	14.00	13.94	13.50
ProBiS	1.00	13.97	13.97	13.99	13.99	14.00	14.00	14.00
RAPMAD	0.82	13.97	14.00	13.92	12.93	11.71	10.75	9.88
Shaper	0.93	13.97	14.00	14.00	13.96	13.84	13.16	12.05
Shaper (PDB)	0.93	13.97	14.00	14.00	13.96	13.84	13.22	12.16
VolSite/Shaper	0.78	13.97	14.00	14.00	14.00	13.96	13.61	12.31
VolSite/Shaper (PDB)	0.76	13.97	14.00	14.00	14.00	13.94	13.24	11.64
SiteAlign	1.00	13.97	14.00	14.00	14.00	14.00	14.00	14.00
SiteEngine	1.00	13.97	14.00	14.00	14.00	14.00	14.00	14.00
SiteHopper	1.00	13.97	14.00	14.00	14.00	14.00	14.00	14.00
SMAP	1.00	13.97	14.00	14.00	14.00	14.00	14.00	14.00
TIFP	0.91	13.97	13.64	13.05	11.74	10.80	9.56	8.38
TIFP (PDB)	0.78	5.95	12.39	12.63	11.24	9.85	8.80	7.77
TM-align	1.00	13.97	14.00	14.00	14.00	14.00	14.00	14.00
