# Supplementary material for: A benchmark driven guide to binding site comparison: An exhaustive evaluation using tailor-made data sets (ProSPECCTs)
Source: PLoS Comput Biol. 2018 Nov 8;14(11):e1006483. doi: 10.1371/journal.pcbi.1006483 (PMC6224041; doi:10.1371/journal.pcbi.1006483)
Supplement: S19 Table — P-values below 0.05 are colored green. (PDF) [file pcbi.1006483.s020.pdf]

S19 Table. AUC confidence intervals for the ROC curves of different binding site comparison methods and AUC value differences with the corresponding p-values calculated according to DeLong and co-workers[1] for data set 2. P-values below 0.05 are colored green.

method	Cavbase	FuzCav (PDB)	FuzCav	Grim (PDB)	Grim	IsoMIF	KRIPO	PocketMatch	ProBiS	RAPMAD
CI	0.86 - 0.88	0.98 - 0.99	0.98 - 0.99	0.84 - 0.85	0.91 - 0.92	0.69 - 0.70	0.96 - 0.96	0.96 - 0.96	1.00 - 1.00	0.81 - 0.83
Cavbase	0.00	0.11	0.11	-0.02	0.04	-0.18	0.09	0.09	0.13	-0.05
	1.00	0.00	0.00	0.00	0.00	0.00	0.00	0.00	0.00	0.00
FuzCav (PDB)	-0.11	0.00	0.00	-0.14	-0.07	-0.29	-0.02	-0.03	0.01	-0.17
	0.00	1.00	0.44	0.00	0.00	0.00	0.00	0.00	0.00	0.00
FuzCav	-0.11	0.00	0.00	-0.14	-0.07	-0.29	-0.02	-0.03	0.01	-0.17
	0.00	0.44	1.00	0.00	0.00	0.00	0.00	0.00	0.00	0.00
Grim (PDB)	0.02	0.14	0.14	0.00	0.07	-0.15	0.11	0.11	0.15	-0.03
	0.00	0.00	0.00	1.00	0.00	0.00	0.00	0.00	0.00	0.00
Grim	-0.04	0.07	0.07	-0.07	0.00	-0.22	0.05	0.04	0.08	-0.10
	0.00	0.00	0.00	0.00	1.00	0.00	0.00	0.00	0.00	0.00
IsoMIF	0.18	0.29	0.29	0.15	0.22	0.00	0.27	0.26	0.30	0.12
	0.00	0.00	0.00	0.00	0.00	1.00	0.00	0.00	0.00	0.00
KRIPO	-0.09	0.02	0.02	-0.11	-0.05	-0.27	0.00	0.00	0.04	-0.14
	0.00	0.00	0.00	0.00	0.00	0.00	1.00	0.18	0.00	0.00
PocketMatch	-0.09	0.03	0.03	-0.11	-0.04	-0.26	0.00	0.00	0.04	-0.14
	0.00	0.00	0.00	0.00	0.00	0.00	0.18	1.00	0.00	0.00
ProBiS	-0.13	-0.01	-0.01	-0.15	-0.08	-0.30	-0.04	-0.04	0.00	-0.18
	0.00	0.00	0.00	0.00	0.00	0.00	0.00	0.00	1.00	0.00
RAPMAD	0.05	0.17	0.17	0.03	0.10	-0.12	0.14	0.14	0.18	0.00
	0.00	0.00	0.00	0.00	0.00	0.00	0.00	0.00	0.00	1.00
VolSite/ Shaper (PDB)	0.11	0.23	0.23	0.09	0.16	-0.06	0.20	0.20	0.24	0.06
	0.00	0.00	0.00	0.00	0.00	0.00	0.00	0.00	0.00	0.00
VolSite/ Shaper	0.09	0.20	0.20	0.06	0.13	-0.09	0.18	0.17	0.21	0.03
	0.00	0.00	0.00	0.00	0.00	0.00	0.00	0.00	0.00	0.00
Shaper (PDB)	-0.06	0.05	0.06	-0.08	-0.01	-0.23	0.03	0.03	0.07	-0.11
	0.00	0.00	0.00	0.00	0.00	0.00	0.00	0.00	0.00	0.00
Shaper	-0.06	0.06	0.06	-0.08	-0.01	-0.23	0.03	0.03	0.07	-0.11
	0.00	0.00	0.00	0.00	0.00	0.00	0.00	0.00	0.00	0.00
SiteAlign	-0.13	-0.01	-0.01	-0.15	-0.08	-0.30	-0.04	-0.04	0.00	-0.18
	0.00	0.00	0.00	0.00	0.00	0.00	0.00	0.00	0.29	0.00
SiteEngine	-0.13	-0.01	-0.01	-0.15	-0.08	-0.30	-0.04	-0.04	0.00	-0.18
	0.00	0.00	0.00	0.00	0.00	0.00	0.00	0.00	0.10	0.00
SiteHopper	-0.13	-0.02	-0.01	-0.15	-0.08	-0.30	-0.04	-0.04	0.00	-0.18
	0.00	0.00	0.00	0.00	0.00	0.00	0.00	0.00	0.00	0.00
SMAP	-0.13	-0.02	-0.01	-0.15	-0.08	-0.30	-0.04	-0.04	0.00	-0.18
	0.00	0.00	0.00	0.00	0.00	0.00	0.00	0.00	0.00	0.00
TIFP (PDB)	0.09	0.20	0.20	0.07	0.13	-0.09	0.18	0.18	0.22	0.04
	0.00	0.00	0.00	0.00	0.00	0.00	0.00	0.00	0.00	0.00
TIFP	-0.04	0.08	0.08	-0.06	0.01	-0.21	0.05	0.05	0.09	-0.09
	0.00	0.00	0.00	0.00	0.01	0.00	0.00	0.00	0.00	0.00
TM-align	-0.13	-0.02	-0.01	-0.15	-0.08	-0.30	-0.04	-0.04	0.00	-0.18
	0.00	0.00	0.00	0.00	0.00	0.00	0.00	0.00	0.00	0.00

S19 Table *(continued)*. AUC confidence intervals for the ROC curves of different binding site comparison methods and AUC value differences with the corresponding p-values calculated according to DeLong and co-workers for data set 2. P-values below 0.05 are colored green.

method	VolSite/ Shaper (PDB)	VolSite/ Shaper	Shaper (PDB)	Shaper	SiteAlign	SiteEngine	SiteHopper	SMAP	TIFP (PDB)	TIFP	TM-align
CI	0.75 - 0.76	0.78 - 0.79	0.93 - 0.93	0.92 - 0.93	1.00 - 1.00	1.00 - 1.00	1.00 - 1.00	1.00 - 1.00	0.78 - 0.79	0.90 - 0.91	1.00 - 1.00
Cavbase	-0.11	-0.09	0.06	0.06	0.13	0.13	0.13	0.13	-0.09	0.04	0.13
	0.00	0.00	0.00	0.00	0.00	0.00	0.00	0.00	0.00	0.00	0.00
FuzCav (PDB)	-0.23	-0.20	-0.05	-0.06	0.01	0.01	0.02	0.02	-0.20	-0.08	0.02
	0.00	0.00	0.00	0.00	0.00	0.00	0.00	0.00	0.00	0.00	0.00
FuzCav	-0.23	-0.20	-0.06	-0.06	0.01	0.01	0.01	0.01	-0.20	-0.08	0.01
	0.00	0.00	0.00	0.00	0.00	0.00	0.00	0.00	0.00	0.00	0.00
Grim (PDB)	-0.09	-0.06	0.08	0.08	0.15	0.15	0.15	0.15	-0.07	0.06	0.15
	0.00	0.00	0.00	0.00	0.00	0.00	0.00	0.00	0.00	0.00	0.00
Grim	-0.16	-0.13	0.01	0.01	0.08	0.08	0.08	0.08	-0.13	-0.01	0.08
	0.00	0.00	0.00	0.00	0.00	0.00	0.00	0.00	0.00	0.01	0.00
IsoMIF	0.06	0.09	0.23	0.23	0.30	0.30	0.30	0.30	0.09	0.21	0.30
	0.00	0.00	0.00	0.00	0.00	0.00	0.00	0.00	0.00	0.00	0.00
KRIPO	-0.20	-0.18	-0.03	-0.03	0.04	0.04	0.04	0.04	-0.18	-0.05	0.04
	0.00	0.00	0.00	0.00	0.00	0.00	0.00	0.00	0.00	0.00	0.00
PocketMatch	-0.20	-0.17	-0.03	-0.03	0.04	0.04	0.04	0.04	-0.18	-0.05	0.04
	0.00	0.00	0.00	0.00	0.00	0.00	0.00	0.00	0.00	0.00	0.00
ProBiS	-0.24	-0.21	-0.07	-0.07	0.00	0.00	0.00	0.00	-0.22	-0.09	0.00
	0.00	0.00	0.00	0.00	0.29	0.10	0.00	0.00	0.00	0.00	0.00
RAPMAD	-0.06	-0.03	0.11	0.11	0.18	0.18	0.18	0.18	-0.04	0.09	0.18
	0.00	0.00	0.00	0.00	0.00	0.00	0.00	0.00	0.00	0.00	0.00
VolSite/ Shaper (PDB)	0.00	0.03	0.17	0.17	0.24	0.24	0.24	0.24	0.03	0.15	0.24
	1.00	0.00	0.00	0.00	0.00	0.00	0.00	0.00	0.00	0.00	0.00
VolSite/ Shaper	-0.03	0.00	0.15	0.14	0.21	0.21	0.22	0.22	0.00	0.12	0.22
	0.00	1.00	0.00	0.00	0.00	0.00	0.00	0.00	0.70	0.00	0.00
Shaper (PDB)	-0.17	-0.15	0.00	0.00	0.07	0.07	0.07	0.07	-0.15	-0.02	0.07
	0.00	0.00	1.00	0.76	0.00	0.00	0.00	0.00	0.00	0.00	0.00
Shaper	-0.17	-0.14	0.00	0.00	0.07	0.07	0.07	0.07	-0.15	-0.02	0.07
	0.00	0.00	0.76	1.00	0.00	0.00	0.00	0.00	0.00	0.00	0.00
SiteAlign	-0.24	-0.21	-0.07	-0.07	0.00	0.00	0.00	0.00	-0.22	-0.09	0.00
	0.00	0.00	0.00	0.00	1.00	0.00	0.00	0.00	0.00	0.00	0.00
SiteEngine	-0.24	-0.21	-0.07	-0.07	0.00	0.00	0.00	0.00	-0.22	-0.09	0.00
	0.00	0.00	0.00	0.00	0.00	1.00	0.00	0.00	0.00	0.00	0.00
SiteHopper	-0.24	-0.22	-0.07	-0.07	0.00	0.00	0.00	0.00	-0.22	-0.09	0.00
	0.00	0.00	0.00	0.00	0.00	0.00	1.00	0.07	0.00	0.00	0.00
SMAP	-0.24	-0.22	-0.07	-0.07	0.00	0.00	0.00	0.00	-0.22	-0.09	0.00
	0.00	0.00	0.00	0.00	0.00	0.00	0.07	1.00	0.00	0.00	0.00
TIFP (PDB)	-0.03	0.00	0.15	0.15	0.22	0.22	0.22	0.22	0.00	0.13	0.22
	0.00	0.70	0.00	0.00	0.00	0.00	0.00	0.00	1.00	0.00	0.00
TIFP	-0.15	-0.12	0.02	0.02	0.09	0.09	0.09	0.09	-0.13	0.00	0.09
	0.00	0.00	0.00	0.00	0.00	0.00	0.00	0.00	0.00	1.00	0.00
TM-align	-0.24	-0.22	-0.07	-0.07	0.00	0.00	0.00	0.00	-0.22	-0.09	0.00
	0.00	0.00	0.00	0.00	0.00	0.00	0.00	0.00	0.00	0.00	1.00

REFERENCES

1. DeLong ER, DeLong DM, Clarke-Pearson DL. Comparing the areas under two or more correlated receiver operating characteristic curves: A nonparametric approach. Biometrics. 1988;44(3):837–45. PubMed PMID: 3203132.
