# Supplementary material for: A benchmark driven guide to binding site comparison: An exhaustive evaluation using tailor-made data sets (ProSPECCTs)
Source: PLoS Comput Biol. 2018 Nov 8;14(11):e1006483. doi: 10.1371/journal.pcbi.1006483 (PMC6224041; doi:10.1371/journal.pcbi.1006483)
Supplement: S24 Table — (PDF) [file pcbi.1006483.s025.pdf]

**S24 Table.** AUC and EFs of different binding site comparison methods for data set 5.

| method | AUC | $EF_{0.1\%}$ | $EF_{0.5\%}$ | $EF_{1\%}$ | $EF_{2\%}$ | $EF_{3\%}$ | $EF_{4\%}$ | $EF_{5\%}$ |
|---|---|---|---|---|---|---|---|---|
| Cavbase | 0.60 | 6.52 | 6.96 | 6.96 | 4.89 | 3.51 | 3.23 | 2.89 |
| FuzCav | 0.55 | 6.52 | 6.96 | 6.96 | 4.89 | 3.33 | 2.83 | 2.35 |
| FuzCav (PDB) | 0.56 | 6.52 | 6.96 | 6.96 | 4.89 | 3.33 | 2.88 | 2.43 |
| Grim | 0.69 | 6.52 | 6.96 | 6.20 | 5.87 | 5.36 | 4.73 | 4.39 |
| Grim (PDB) | 0.61 | 0.00 | 2.39 | 4.67 | 3.75 | 3.44 | 2.66 | 2.13 |
| IsoMIF | 0.75 | 6.52 | 6.96 | 6.96 | 5.43 | 5.18 | 5.14 | 4.76 |
| KRIPO | 0.76 | 6.52 | 6.96 | 6.96 | 5.87 | 5.07 | 4.62 | 4.22 |
| PocketMatch | 0.66 | 6.52 | 6.96 | 6.96 | 5.33 | 4.20 | 3.75 | 3.57 |
| ProBiS | 0.54 | 6.52 | 6.96 | 6.96 | 5.05 | 3.73 | 3.34 | 2.91 |
| RAPMAD | 0.55 | 6.52 | 6.96 | 6.96 | 4.89 | 3.48 | 2.72 | 2.35 |
| Shaper | 0.65 | 6.52 | 6.96 | 6.96 | 5.11 | 4.35 | 3.94 | 3.48 |
| Shaper (PDB) | 0.66 | 6.52 | 6.96 | 6.96 | 5.22 | 4.49 | 4.02 | 3.70 |
| VolSite/Shaper | 0.56 | 6.52 | 6.96 | 6.52 | 4.13 | 3.15 | 2.74 | 2.41 |
| VolSite/Shaper (PDB) | 0.57 | 6.52 | 6.96 | 6.09 | 4.02 | 3.12 | 2.77 | 2.50 |
| SiteAlign | 0.59 | 6.52 | 6.96 | 6.96 | 4.89 | 3.80 | 3.10 | 2.70 |
| SiteEngine | 0.64 | 6.52 | 6.96 | 6.96 | 5.38 | 4.35 | 3.64 | 3.17 |
| SiteHopper | 0.72 | 6.52 | 6.96 | 6.96 | 5.82 | 4.82 | 4.21 | 3.76 |
| SMAP | 0.62 | 6.52 | 6.96 | 6.96 | 5.11 | 3.91 | 3.07 | 2.78 |
| TIFP | 0.71 | 6.52 | 6.96 | 6.96 | 6.03 | 5.04 | 4.29 | 4.02 |
| TIFP (PDB) | 0.54 | 0.00 | 3.91 | 5.33 | 3.32 | 2.57 | 2.53 | 2.54 |
| TM-align | 0.66 | 6.52 | 6.96 | 6.96 | 5.76 | 4.64 | 3.89 | 3.57 |
