# Supplementary material for: A benchmark driven guide to binding site comparison: An exhaustive evaluation using tailor-made data sets (ProSPECCTs)
Source: PLoS Comput Biol. 2018 Nov 8;14(11):e1006483. doi: 10.1371/journal.pcbi.1006483 (PMC6224041; doi:10.1371/journal.pcbi.1006483)
Supplement: S25 Table — P-values below 0.05 are colored green. (PDF) [file pcbi.1006483.s026.pdf]

S25 Table. AUC confidence intervals for the ROC curves of different binding site comparison methods and AUC value differences with the corresponding p-values calculated according to DeLong and co-workers[1] for data set 5. P-values below 0.05 are colored green.

method	Cavbase	FuzCav (PDB)	FuzCav	Grim (PDB)	Grim	IsoMIF	KRIPO	PocketMatch	ProBiS	RAPMAD
CI	0.58 - 0.63	0.54 - 0.58	0.53 - 0.58	0.59 - 0.63	0.67 - 0.71	0.73 - 0.78	0.75 - 0.78	0.64 - 0.68	0.53 - 0.56	0.53 - 0.57
Cavbase	0.00	-0.05	-0.05	0.00	0.08	0.15	0.16	0.06	-0.06	-0.05
	1.00	0.00	0.00	0.91	0.00	0.00	0.00	0.00	0.00	0.00
FuzCav (PDB)	0.05	0.00	0.00	0.05	0.13	0.20	0.21	0.11	-0.01	-0.01
	0.00	1.00	0.94	0.00	0.00	0.00	0.00	0.00	0.33	0.67
FuzCav	0.05	0.00	0.00	0.05	0.13	0.20	0.21	0.11	-0.01	-0.01
	0.00	0.94	1.00	0.00	0.00	0.00	0.00	0.00	0.38	0.73
Grim (PDB)	0.00	-0.05	-0.05	0.00	0.08	0.15	0.16	0.06	-0.06	-0.06
	0.91	0.00	0.00	1.00	0.00	0.00	0.00	0.00	0.00	0.00
Grim	-0.08	-0.13	-0.13	-0.08	0.00	0.07	0.08	-0.03	-0.15	-0.14
	0.00	0.00	0.00	0.00	1.00	0.00	0.00	0.09	0.00	0.00
IsoMIF	-0.15	-0.20	-0.20	-0.15	-0.07	0.00	0.01	-0.09	-0.21	-0.20
	0.00	0.00	0.00	0.00	0.00	1.00	0.49	0.00	0.00	0.00
KRIPO	-0.16	-0.21	-0.21	-0.16	-0.08	-0.01	0.00	-0.10	-0.22	-0.21
	0.00	0.00	0.00	0.00	0.00	0.49	1.00	0.00	0.00	0.00
PocketMatch	-0.06	-0.11	-0.11	-0.06	0.03	0.09	0.10	0.00	-0.12	-0.11
	0.00	0.00	0.00	0.00	0.09	0.00	0.00	1.00	0.00	0.00
ProBiS	0.06	0.01	0.01	0.06	0.15	0.21	0.22	0.12	0.00	0.01
	0.00	0.33	0.38	0.00	0.00	0.00	0.00	0.00	1.00	0.63
RAPMAD	0.05	0.01	0.01	0.06	0.14	0.20	0.21	0.11	-0.01	0.00
	0.00	0.67	0.73	0.00	0.00	0.00	0.00	0.00	0.63	1.00
VolSite/ Shaper (PDB)	0.04	-0.01	-0.01	0.04	0.12	0.19	0.20	0.09	-0.03	-0.02
	0.02	0.40	0.36	0.01	0.00	0.00	0.00	0.00	0.05	0.21
VolSite/ Shaper	0.04	0.00	-0.01	0.05	0.13	0.19	0.20	0.10	-0.02	-0.01
	0.00	0.77	0.71	0.00	0.00	0.00	0.00	0.00	0.19	0.48
Shaper (PDB)	-0.06	-0.11	-0.11	-0.06	0.02	0.09	0.10	0.00	-0.12	-0.11
	0.00	0.00	0.00	0.00	0.12	0.00	0.00	0.93	0.00	0.00
Shaper	-0.05	-0.10	-0.10	-0.05	0.03	0.10	0.11	0.01	-0.11	-0.10
	0.00	0.00	0.00	0.00	0.02	0.00	0.00	0.52	0.00	0.00
SiteAlign	0.02	-0.03	-0.03	0.02	0.10	0.17	0.18	0.08	-0.04	-0.04
	0.26	0.04	0.03	0.20	0.00	0.00	0.00	0.00	0.00	0.01
SiteEngine	-0.03	-0.08	-0.08	-0.03	0.05	0.12	0.13	0.03	-0.09	-0.09
	0.04	0.00	0.00	0.05	0.00	0.00	0.00	0.07	0.00	0.00
SiteHopper	-0.12	-0.17	-0.17	-0.12	-0.04	0.03	0.04	-0.06	-0.18	-0.17
	0.00	0.00	0.00	0.00	0.01	0.03	0.00	0.00	0.00	0.00
SMAP	-0.01	-0.06	-0.06	-0.01	0.07	0.14	0.15	0.04	-0.08	-0.07
	0.34	0.00	0.00	0.39	0.00	0.00	0.00	0.00	0.00	0.00
TIFP (PDB)	0.07	0.02	0.02	0.07	0.15	0.22	0.23	0.12	0.00	0.01
	0.00	0.21	0.24	0.00	0.00	0.00	0.00	0.00	0.71	0.43
TIFP	-0.10	-0.15	-0.15	-0.10	-0.02	0.05	0.06	-0.04	-0.16	-0.16
	0.00	0.00	0.00	0.00	0.19	0.00	0.00	0.00	0.00	0.00
TM-align	-0.05	-0.10	-0.10	-0.05	0.03	0.10	0.10	0.00	-0.12	-0.11
	0.00	0.00	0.00	0.00	0.05	0.00	0.00	0.80	0.00	0.00

S25 Table *(continued)*. AUC confidence intervals for the ROC curves of different binding site comparison methods and AUC value differences with the corresponding p-values calculated according to DeLong and co-workers[1] for data set 5. P-values below 0.05 are colored green.

method	VolSite/ Shaper (PDB)	VolSite/ Shaper	Shaper (PDB)	Shaper	SiteAlign	SiteEngine	SiteHopper	SMAP	TIFP (PDB)	TIFP	TM-align
CI	0.55 - 0.59	0.54 - 0.58	0.64 - 0.69	0.63 - 0.67	0.57 - 0.61	0.62 - 0.66	0.71 - 0.74	0.60 - 0.64	0.52 - 0.56	0.69 - 0.73	0.64 - 0.68
Cavbase	-0.04	-0.04	0.06	0.05	-0.02	0.03	0.12	0.01	-0.07	0.10	0.05
	0.02	0.00	0.00	0.00	0.26	0.04	0.00	0.34	0.00	0.00	0.00
FuzCav (PDB)	0.01	0.00	0.11	0.10	0.03	0.08	0.17	0.06	-0.02	0.15	0.10
	0.40	0.77	0.00	0.00	0.04	0.00	0.00	0.00	0.21	0.00	0.00
FuzCav	0.01	0.01	0.11	0.10	0.03	0.08	0.17	0.06	-0.02	0.15	0.10
	0.36	0.71	0.00	0.00	0.03	0.00	0.00	0.00	0.24	0.00	0.00
Grim (PDB)	-0.04	-0.05	0.06	0.05	-0.02	0.03	0.12	0.01	-0.07	0.10	0.05
	0.01	0.00	0.00	0.00	0.20	0.05	0.00	0.39	0.00	0.00	0.00
Grim	-0.12	-0.13	-0.02	-0.03	-0.10	-0.05	0.04	-0.07	-0.15	0.02	-0.03
	0.00	0.00	0.12	0.02	0.00	0.00	0.01	0.00	0.00	0.19	0.05
IsoMIF	-0.19	-0.19	-0.09	-0.10	-0.17	-0.12	-0.03	-0.14	-0.22	-0.05	-0.10
	0.00	0.00	0.00	0.00	0.00	0.00	0.03	0.00	0.00	0.00	0.00
KRIPO	-0.20	-0.20	-0.10	-0.11	-0.18	-0.13	-0.04	-0.15	-0.23	-0.06	-0.10
	0.00	0.00	0.00	0.00	0.00	0.00	0.00	0.00	0.00	0.00	0.00
PocketMatch	-0.09	-0.10	0.00	-0.01	-0.08	-0.03	0.06	-0.04	-0.12	0.04	0.00
	0.00	0.00	0.93	0.52	0.00	0.07	0.00	0.00	0.00	0.00	0.80
ProBiS	0.03	0.02	0.12	0.11	0.04	0.09	0.18	0.08	0.00	0.16	0.12
	0.05	0.19	0.00	0.00	0.00	0.00	0.00	0.00	0.71	0.00	0.00
RAPMAD	0.02	0.01	0.11	0.10	0.04	0.09	0.17	0.07	-0.01	0.16	0.11
	0.21	0.48	0.00	0.00	0.01	0.00	0.00	0.00	0.43	0.00	0.00
VolSite/ Shaper (PDB)	0.00	-0.01	0.10	0.08	0.02	0.07	0.16	0.05	-0.03	0.14	0.09
	1.00	0.58	0.00	0.00	0.21	0.00	0.00	0.00	0.03	0.00	0.00
VolSite/ Shaper	0.01	0.00	0.10	0.09	0.03	0.08	0.16	0.06	-0.02	0.15	0.10
	0.58	1.00	0.00	0.00	0.07	0.00	0.00	0.00	0.11	0.00	0.00
Shaper (PDB)	-0.10	-0.10	0.00	-0.01	-0.08	-0.03	0.06	-0.05	-0.13	0.04	-0.01
	0.00	0.00	1.00	0.48	0.00	0.06	0.00	0.00	0.00	0.00	0.74
Shaper	-0.08	-0.09	0.01	0.00	-0.07	-0.02	0.07	-0.03	-0.12	0.05	0.01
	0.00	0.00	0.48	1.00	0.00	0.25	0.00	0.02	0.00	0.00	0.70
SiteAlign	-0.02	-0.03	0.08	0.07	0.00	0.05	0.14	0.03	-0.05	0.12	0.07
	0.21	0.07	0.00	0.00	1.00	0.00	0.00	0.03	0.00	0.00	0.00
SiteEngine	-0.07	-0.08	0.03	0.02	-0.05	0.00	0.09	-0.02	-0.10	0.07	0.02
	0.00	0.00	0.06	0.25	0.00	1.00	0.00	0.24	0.00	0.00	0.11
SiteHopper	-0.16	-0.16	-0.06	-0.07	-0.14	-0.09	0.00	-0.11	-0.19	-0.02	-0.06
	0.00	0.00	0.00	0.00	0.00	0.00	1.00	0.00	0.00	0.23	0.00
SMAP	-0.05	-0.06	0.05	0.03	-0.03	0.02	0.11	0.00	-0.08	0.09	0.04
	0.00	0.00	0.00	0.02	0.03	0.24	0.00	1.00	0.00	0.00	0.01
TIFP (PDB)	0.03	0.02	0.13	0.12	0.05	0.10	0.19	0.08	0.00	0.17	0.12
	0.03	0.11	0.00	0.00	0.00	0.00	0.00	0.00	1.00	0.00	0.00
TIFP	-0.14	-0.15	-0.04	-0.05	-0.12	-0.07	0.02	-0.09	-0.17	0.00	-0.05
	0.00	0.00	0.00	0.00	0.00	0.00	0.23	0.00	0.00	1.00	0.00
TM-align	-0.09	-0.10	0.01	-0.01	-0.07	-0.02	0.06	-0.04	-0.12	0.05	0.00
	0.00	0.00	0.74	0.70	0.00	0.11	0.00	0.01	0.00	0.00	1.00

REFERENCES

1. DeLong ER, DeLong DM, Clarke-Pearson DL. Comparing the areas under two or more correlated receiver operating characteristic curves: A nonparametric approach. Biometrics. 1988;44(3):837–45. PubMed PMID: 3203132.
