# Supplementary material for: A benchmark driven guide to binding site comparison: An exhaustive evaluation using tailor-made data sets (ProSPECCTs)
Source: PLoS Comput Biol. 2018 Nov 8;14(11):e1006483. doi: 10.1371/journal.pcbi.1006483 (PMC6224041; doi:10.1371/journal.pcbi.1006483)
Supplement: S27 Table — P-values below 0.05 are colored green. (PDF) [file pcbi.1006483.s028.pdf]

S27 Table. AUC confidence intervals for the ROC curves of different binding site comparison methods and AUC value differences with the corresponding p-values calculated according to DeLong and co-workers[1] for data set 5.2. P-values below 0.05 are colored green.

method	Cavbase	FuzCav (PDB)	FuzCav	Grim (PDB)	Grim	IsoMIF	KRIPO	PocketMatch	ProBiS	RAPMAD
CI	0.55 - 0.58	0.52 - 0.56	0.52 - 0.55	0.56 - 0.59	0.60 - 0.63	0.79 - 0.83	0.76 - 0.79	0.58 - 0.62	0.53 - 0.56	0.50 - 0.53
Cavbase	0.00	-0.03	-0.03	0.01	0.05	0.25	0.21	0.03	-0.02	-0.05
	1.00	0.04	0.03	0.34	0.00	0.00	0.00	0.01	0.11	0.00
FuzCav (PDB)	0.03	0.00	0.00	0.04	0.08	0.27	0.24	0.06	0.01	-0.02
	0.04	1.00	0.95	0.00	0.00	0.00	0.00	0.00	0.46	0.09
FuzCav	0.03	0.00	0.00	0.04	0.08	0.27	0.24	0.06	0.01	-0.02
	0.03	0.95	1.00	0.00	0.00	0.00	0.00	0.00	0.42	0.10
Grim (PDB)	-0.01	-0.04	-0.04	0.00	0.04	0.23	0.20	0.02	-0.03	-0.06
	0.34	0.00	0.00	1.00	0.00	0.00	0.00	0.08	0.00	0.00
Grim	-0.05	-0.08	-0.08	-0.04	0.00	0.20	0.16	-0.01	-0.07	-0.10
	0.00	0.00	0.00	0.00	1.00	0.00	0.00	0.26	0.00	0.00
IsoMIF	-0.25	-0.27	-0.27	-0.23	-0.20	0.00	-0.04	-0.21	-0.26	-0.30
	0.00	0.00	0.00	0.00	0.00	1.00	0.00	0.00	0.00	0.00
KRIPO	-0.21	-0.24	-0.24	-0.20	-0.16	0.04	0.00	-0.18	-0.23	-0.26
	0.00	0.00	0.00	0.00	0.00	0.00	1.00	0.00	0.00	0.00
PocketMatch	-0.03	-0.06	-0.06	-0.02	0.01	0.21	0.18	0.00	-0.05	-0.08
	0.01	0.00	0.00	0.08	0.26	0.00	0.00	1.00	0.00	0.00
ProBiS	0.02	-0.01	-0.01	0.03	0.07	0.26	0.23	0.05	0.00	-0.03
	0.11	0.46	0.42	0.00	0.00	0.00	0.00	0.00	1.00	0.01
RAPMAD	0.05	0.02	0.02	0.06	0.10	0.30	0.26	0.08	0.03	0.00
	0.00	0.09	0.10	0.00	0.00	0.00	0.00	0.00	0.01	1.00
VolSite/ Shaper (PDB)	0.01	-0.02	-0.02	0.02	0.06	0.26	0.22	0.04	-0.01	-0.04
	0.44	0.13	0.12	0.06	0.00	0.00	0.00	0.00	0.36	0.00
VolSite/ Shaper	-0.02	-0.04	-0.04	0.00	0.03	0.23	0.19	0.02	-0.03	-0.06
	0.25	0.00	0.00	0.79	0.01	0.00	0.00	0.15	0.00	0.00
Shaper (PDB)	-0.08	-0.11	-0.11	-0.07	-0.03	0.17	0.13	-0.04	-0.10	-0.13
	0.00	0.00	0.00	0.00	0.02	0.00	0.00	0.00	0.00	0.00
Shaper	-0.08	-0.11	-0.11	-0.07	-0.04	0.16	0.13	-0.05	-0.10	-0.13
	0.00	0.00	0.00	0.00	0.01	0.00	0.00	0.00	0.00	0.00
SiteAlign	0.00	-0.03	-0.03	0.01	0.05	0.24	0.21	0.03	-0.02	-0.05
	0.81	0.02	0.01	0.47	0.00	0.00	0.00	0.02	0.05	0.00
SiteEngine	-0.01	-0.03	-0.03	0.01	0.04	0.24	0.20	0.03	-0.03	-0.06
	0.65	0.01	0.01	0.65	0.00	0.00	0.00	0.04	0.04	0.00
SiteHopper	-0.25	-0.27	-0.28	-0.24	-0.20	0.00	-0.04	-0.21	-0.27	-0.30
	0.00	0.00	0.00	0.00	0.00	0.92	0.00	0.00	0.00	0.00
SMAP	0.03	0.00	0.00	0.04	0.07	0.27	0.24	0.06	0.01	-0.02
	0.05	0.94	0.90	0.00	0.00	0.00	0.00	0.00	0.52	0.08
TIFP (PDB)	0.03	0.01	0.01	0.05	0.08	0.28	0.24	0.07	0.02	-0.01
	0.00	0.51	0.55	0.00	0.00	0.00	0.00	0.00	0.10	0.19
TIFP	-0.06	-0.09	-0.09	-0.05	-0.01	0.18	0.15	-0.03	-0.08	-0.11
	0.00	0.00	0.00	0.00	0.30	0.00	0.00	0.03	0.00	0.00
TM-align	-0.06	-0.09	-0.09	-0.05	-0.01	0.19	0.15	-0.02	-0.08	-0.11
	0.00	0.00	0.00	0.00	0.44	0.00	0.00	0.06	0.00	0.00

S27 Table *(continued)*. AUC confidence intervals for the ROC curves of different binding site comparison methods and AUC value differences with the corresponding p-values calculated according to DeLong and co-workers[1] for data set 5.2. P-values below 0.05 are colored green.

method	VolSite/ Shaper (PDB)	VolSite/ Shaper	Shaper (PDB)	Shaper	SiteAlign	SiteEngine	SiteHopper	SMAP	TIFP (PDB)	TIFP	TM-align
CI	0.54 - 0.57	0.56 - 0.60	0.63 - 0.66	0.63 - 0.67	0.55 - 0.59	0.55 - 0.59	0.80 - 0.82	0.52 - 0.56	0.52 - 0.54	0.61 - 0.65	0.61 - 0.64
Cavbase	-0.01	0.02	0.08	0.08	0.00	0.01	0.25	-0.03	-0.03	0.06	0.06
	0.44	0.25	0.00	0.00	0.81	0.65	0.00	0.05	0.00	0.00	0.00
FuzCav (PDB)	0.02	0.04	0.11	0.11	0.03	0.03	0.27	0.00	-0.01	0.09	0.09
	0.13	0.00	0.00	0.00	0.02	0.01	0.00	0.94	0.51	0.00	0.00
FuzCav	0.02	0.04	0.11	0.11	0.03	0.03	0.28	0.00	-0.01	0.09	0.09
	0.12	0.00	0.00	0.00	0.01	0.01	0.00	0.90	0.55	0.00	0.00
Grim (PDB)	-0.02	0.00	0.07	0.07	-0.01	-0.01	0.24	-0.04	-0.05	0.05	0.05
	0.06	0.79	0.00	0.00	0.47	0.65	0.00	0.00	0.00	0.00	0.00
Grim	-0.06	-0.03	0.03	0.04	-0.05	-0.04	0.20	-0.07	-0.08	0.01	0.01
	0.00	0.01	0.02	0.01	0.00	0.00	0.00	0.00	0.00	0.30	0.44
IsoMIF	-0.26	-0.23	-0.17	-0.16	-0.24	-0.24	0.00	-0.27	-0.28	-0.18	-0.19
	0.00	0.00	0.00	0.00	0.00	0.00	0.92	0.00	0.00	0.00	0.00
KRIPO	-0.22	-0.19	-0.13	-0.13	-0.21	-0.20	0.04	-0.24	-0.24	-0.15	-0.15
	0.00	0.00	0.00	0.00	0.00	0.00	0.00	0.00	0.00	0.00	0.00
PocketMatch	-0.04	-0.02	0.04	0.05	-0.03	-0.03	0.21	-0.06	-0.07	0.03	0.02
	0.00	0.15	0.00	0.00	0.02	0.04	0.00	0.00	0.00	0.03	0.06
ProBiS	0.01	0.03	0.10	0.10	0.02	0.03	0.27	-0.01	-0.02	0.08	0.08
	0.36	0.00	0.00	0.00	0.05	0.04	0.00	0.52	0.10	0.00	0.00
RAPMAD	0.04	0.06	0.13	0.13	0.05	0.06	0.30	0.02	0.01	0.11	0.11
	0.00	0.00	0.00	0.00	0.00	0.00	0.00	0.08	0.19	0.00	0.00
VolSite/ Shaper (PDB)	0.00	0.02	0.09	0.09	0.01	0.02	0.26	-0.02	-0.03	0.07	0.07
	1.00	0.04	0.00	0.00	0.29	0.21	0.00	0.17	0.01	0.00	0.00
VolSite/ Shaper	-0.02	0.00	0.06	0.07	-0.01	-0.01	0.23	-0.04	-0.05	0.05	0.04
	0.04	1.00	0.00	0.00	0.34	0.50	0.00	0.00	0.00	0.00	0.00
Shaper (PDB)	-0.09	-0.06	0.00	0.01	-0.07	-0.07	0.17	-0.10	-0.11	-0.02	-0.02
	0.00	0.00	1.00	0.67	0.00	0.00	0.00	0.00	0.00	0.22	0.13
Shaper	-0.09	-0.07	-0.01	0.00	-0.08	-0.08	0.16	-0.11	-0.12	-0.02	-0.03
	0.00	0.00	0.67	1.00	0.00	0.00	0.00	0.00	0.00	0.09	0.05
SiteAlign	-0.01	0.01	0.07	0.08	0.00	0.00	0.24	-0.03	-0.04	0.06	0.06
	0.29	0.34	0.00	0.00	1.00	0.82	0.00	0.02	0.00	0.00	0.00
SiteEngine	-0.02	0.01	0.07	0.08	0.00	0.00	0.24	-0.03	-0.04	0.06	0.05
	0.21	0.50	0.00	0.00	0.82	1.00	0.00	0.02	0.00	0.00	0.00
SiteHopper	-0.26	-0.23	-0.17	-0.16	-0.24	-0.24	0.00	-0.27	-0.28	-0.19	-0.19
	0.00	0.00	0.00	0.00	0.00	0.00	1.00	0.00	0.00	0.00	0.00
SMAP	0.02	0.04	0.10	0.11	0.03	0.03	0.27	0.00	-0.01	0.09	0.08
	0.17	0.00	0.00	0.00	0.02	0.02	0.00	1.00	0.47	0.00	0.00
TIFP (PDB)	0.03	0.05	0.11	0.12	0.04	0.04	0.28	0.01	0.00	0.10	0.09
	0.01	0.00	0.00	0.00	0.00	0.00	0.00	0.47	1.00	0.00	0.00
TIFP	-0.07	-0.05	0.02	0.02	-0.06	-0.06	0.19	-0.09	-0.10	0.00	0.00
	0.00	0.00	0.22	0.09	0.00	0.00	0.00	0.00	0.00	1.00	0.78
TM-align	-0.07	-0.04	0.02	0.03	-0.06	-0.05	0.19	-0.08	-0.09	0.00	0.00
	0.00	0.00	0.13	0.05	0.00	0.00	0.00	0.00	0.00	0.78	1.00

REFERENCES

1. DeLong ER, DeLong DM, Clarke-Pearson DL. Comparing the areas under two or more correlated receiver operating characteristic curves: A nonparametric approach. Biometrics. 1988;44(3):837–45. PubMed PMID: 3203132.
