# Supplementary figures and images for: A benchmark driven guide to binding site comparison: An exhaustive evaluation using tailor-made data sets (ProSPECCTs)
Source: PLoS Comput Biol. 2018 Nov 8;14(11):e1006483. doi: 10.1371/journal.pcbi.1006483 (PMC6224041; doi:10.1371/journal.pcbi.1006483)

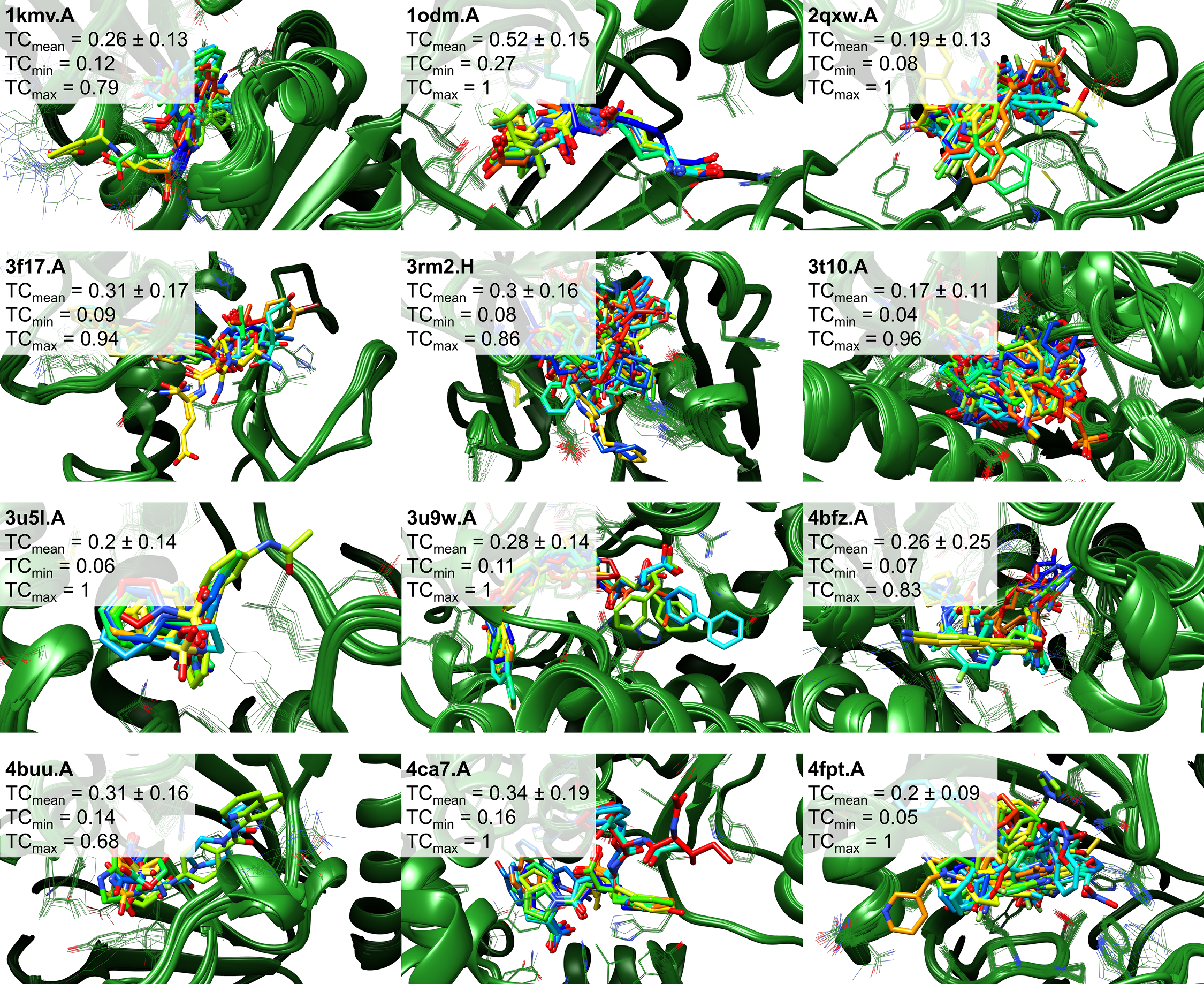

Supplement: S1 Fig — All figures were generated using UCSF Chimera [60]. (PNG) [file pcbi.1006483.s035.png]

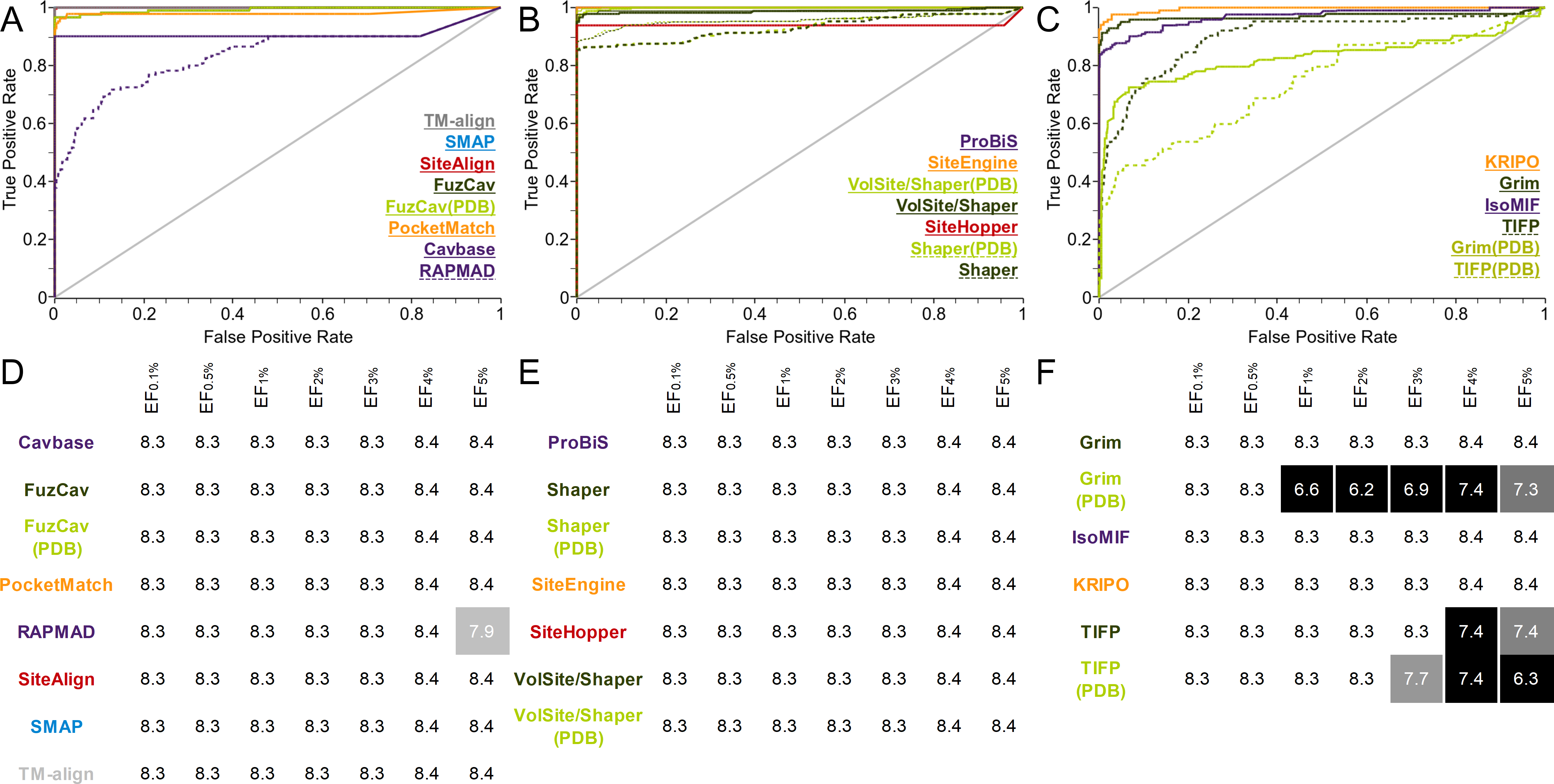

Supplement: S2 Fig — A-C) The ROC curves for residue- (A), surface- (B), and interaction-based (C) comparison methods. The name of the tool is colored according to its corresponding ROC curve. The binding site comparison tools are sorted in descending order with respect to their AUC. (B) The score RefTversky (color) led to the highest AUC values for Shaper, Shaper(PDB), VolSite/Shaper, and VolSite/Shaper(PDB). D-F) EFs for residue- (D), surface- (E), and interaction-based (F) comparison methods. A linear color gradient ranging from white for the highest value to gray to black for the lowest value was applied for the EFs at different percentages of screened data set. (PNG) [file pcbi.1006483.s036.png]

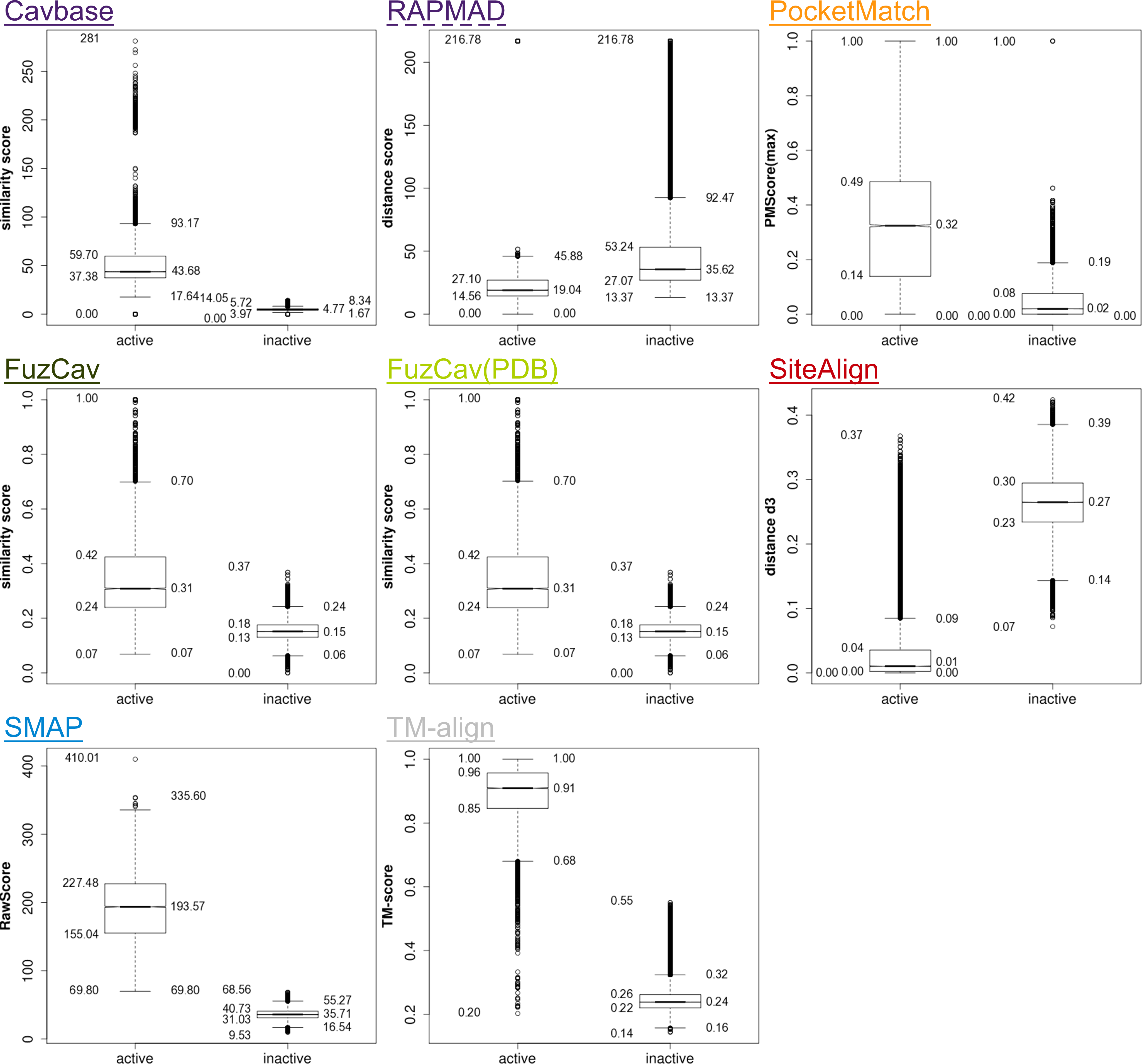

Supplement: S3 Fig — The plots were created based on the data set of structures with identical sequences. The usage of RAPMAD and SiteAlign results in distances for the binding site pairs. The application of the remaining tools results in similarity scores. The values given in the plots correspond to the maximum, the upper whisker, the upper quartile, the median, the lower quartile, the lower whisker, and the minimum. (PNG) [file pcbi.1006483.s037.png]

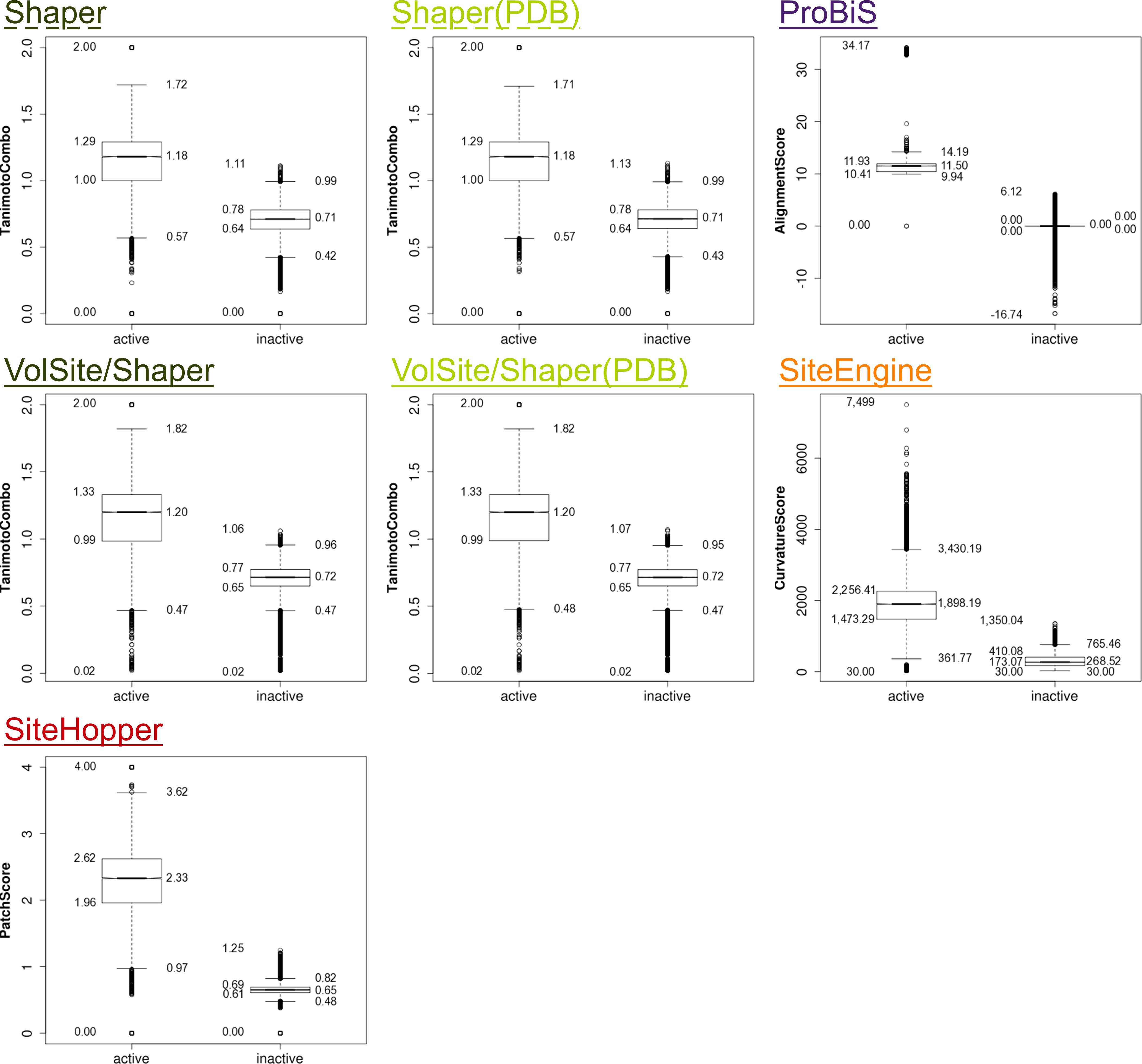

Supplement: S4 Fig — The plots were created based on the data set of structures with identical sequences. The values given in the plots correspond to the maximum, the upper whisker, the upper quartile, the median, the lower quartile, the lower whisker, and the minimum. (PNG) [file pcbi.1006483.s038.png]

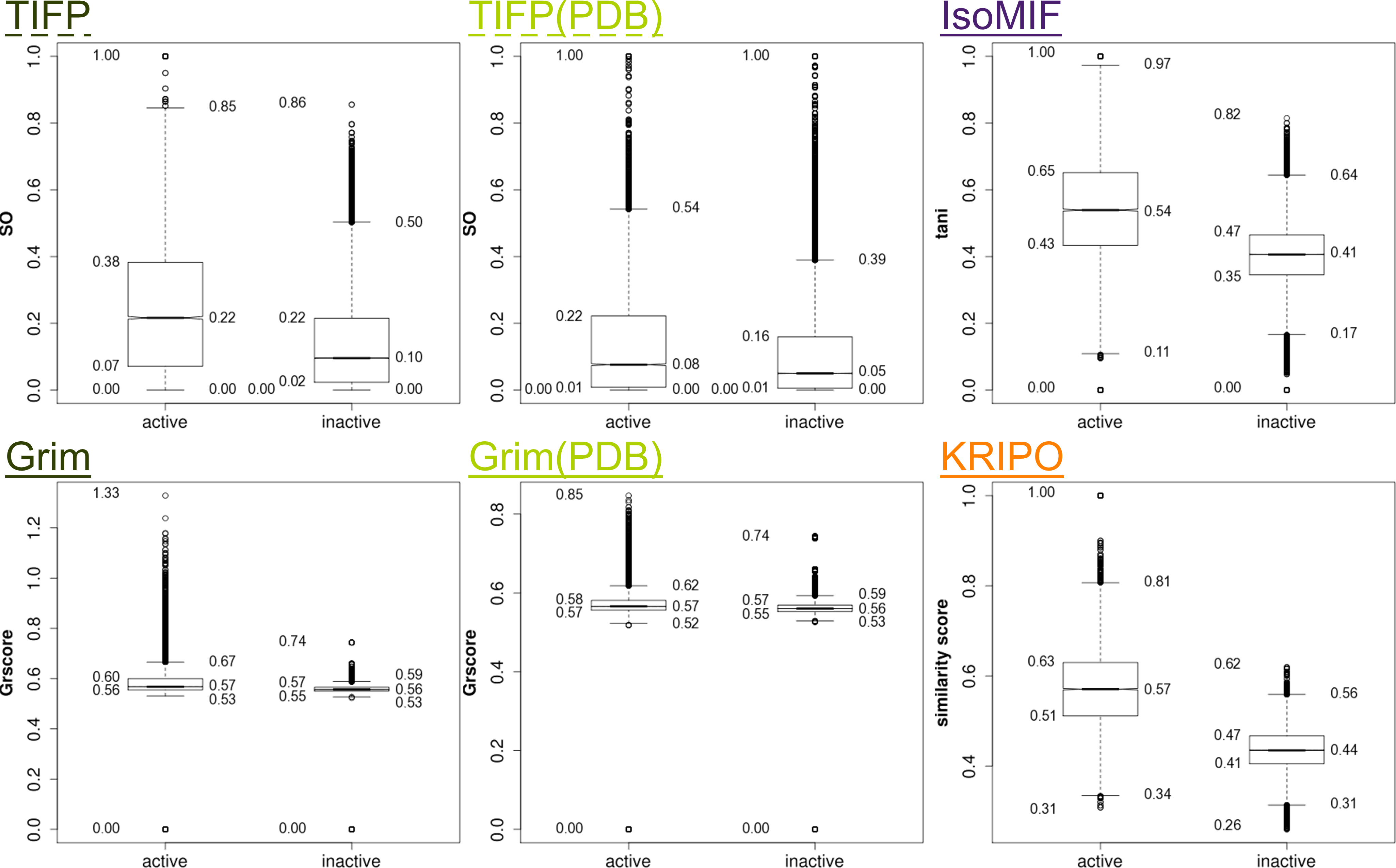

Supplement: S5 Fig — The plots were created based on the data set of structures with identical sequences. The values given in the plots correspond to the maximum, the upper whisker, the upper quartile, the median, the lower quartile, the lower whisker, and the minimum. (PNG) [file pcbi.1006483.s039.png]

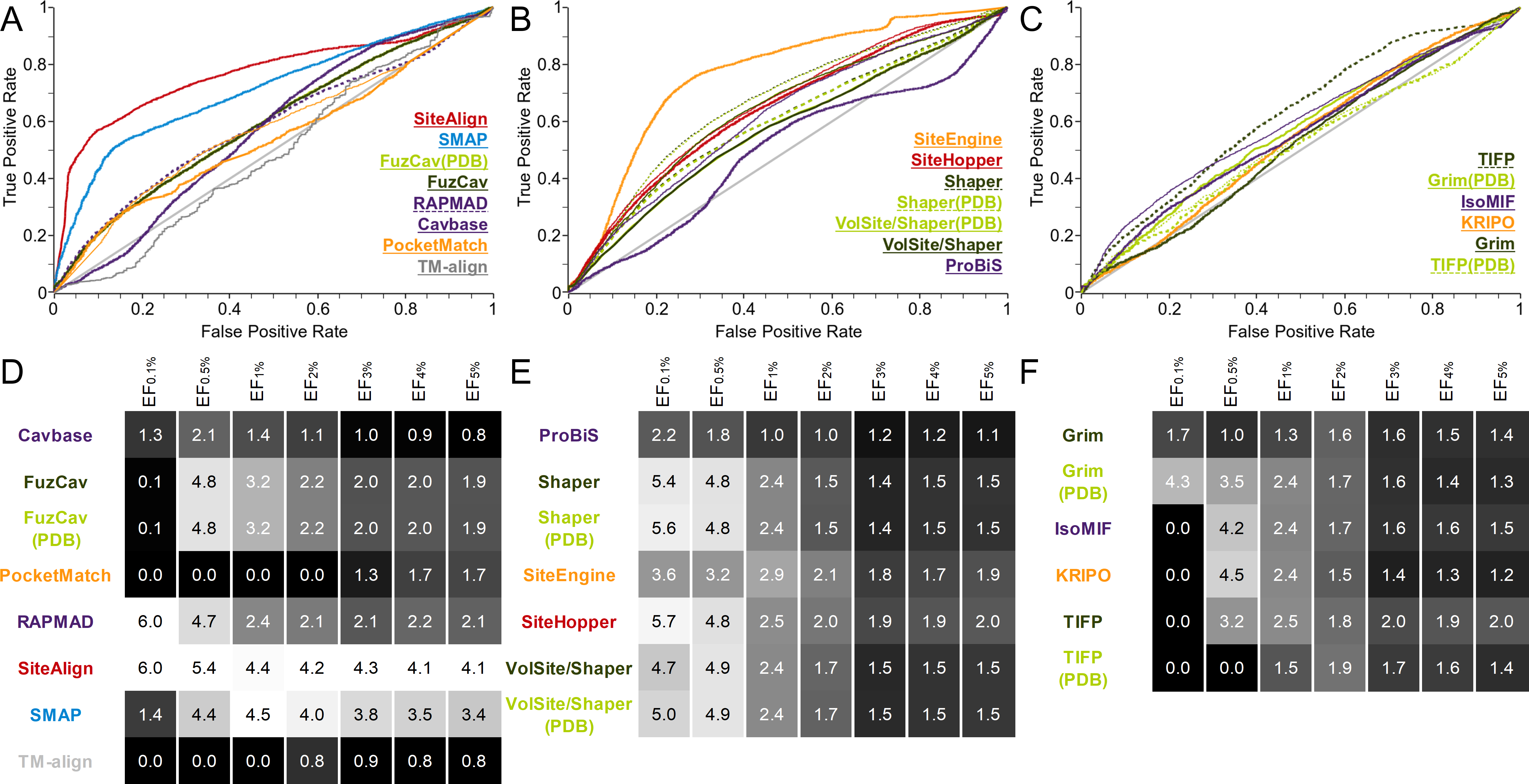

Supplement: S6 Fig — A-C) The ROC curves for residue- (A), surface- (B), and interaction-based (C) comparison methods. The name of the tool is colored according to its corresponding ROC curve. The binding site comparison tools are sorted in descending order with respect to their AUC. (A) PocketMatch showed the best AUC for the score PMScoremin (thin orange line). (B) The scores SVA, RefTversky (color), RefTversky (color), RefTversky (color), RefTversky (color), and ColorTanimoto led to the highest AUC values for ProBiS, Shaper, Shaper(PDB), VolSite/Shaper, VolSite/Shaper(PDB), and SiteHopper, respectively (thin lines). (C) The highest AUC was obtained for IsoMIF and TIFP(PDB) when using taniM and the Tanimoto coefficient, respectively (thin lines). D-F) EFs for residue- (D), surface- (E), and interaction-based (F) comparison methods. A linear color gradient ranging from white for the highest value to gray to black for the lowest value was applied for the EFs at different percentages of screened data set. (PNG) [file pcbi.1006483.s040.png]

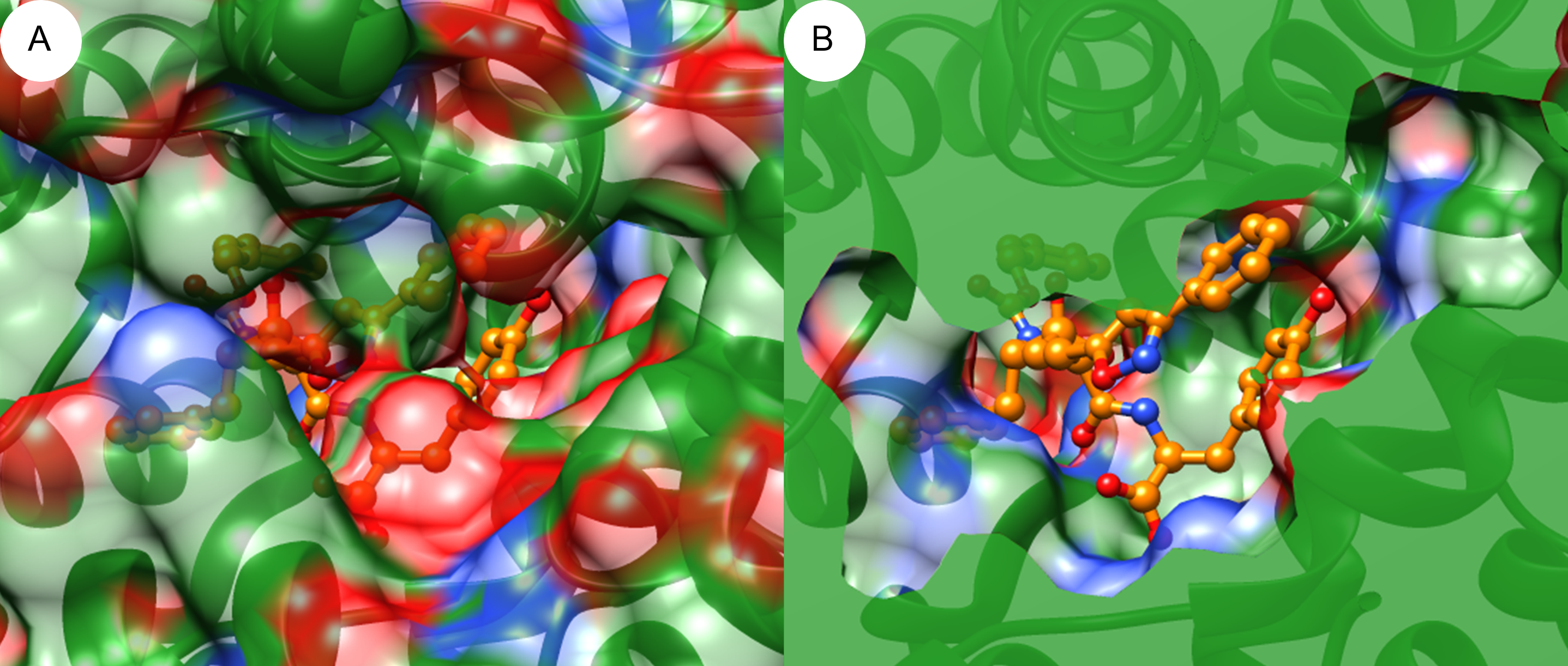

Supplement: S7 Fig — The structure is depicted in green in the surface representation (A) and the clipped surface representation (B). The ligand with the id 3EF is shown as an orange ball-and-stick model. The figure was generated using UCSF Chimera. (PNG) [file pcbi.1006483.s041.png]

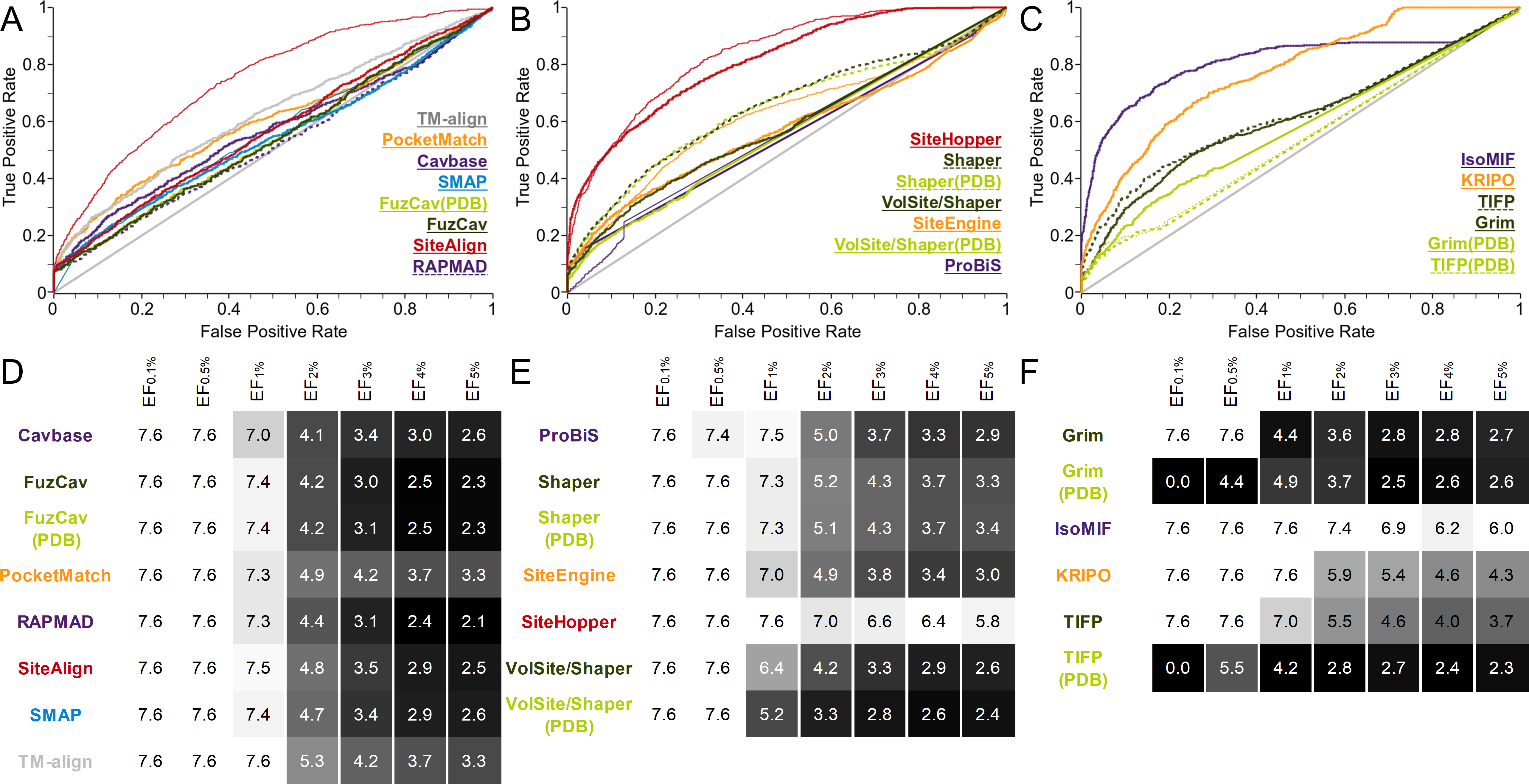

Supplement: S8 Fig — A-C) The ROC curves for residue- (A), surface- (B), and interaction-based (C) comparison methods. The name of the tool is colored according to its corresponding ROC curve. The binding site comparison tools are sorted in descending order with respect to the AUC. (A) The best AUC for SiteAlign resulted from the d1 distance (thin red line). (B) For ProBiS, SiteEngine, and SiteHopper the scores SVA, LowResolutionScore, and ShapeTanimoto yielded the best AUC values (thin lines). (C) For TIFP(PDB), the use of the Hamming distance led to the best results with respect to AUC (thin line). D-F) EFs for residue- (D), surface- (E), and interaction-based (F) comparison methods. A linear color gradient ranging from white for the highest value to gray to black for the lowest value was applied for the EFs at different percentages of screened data set. (PNG) [file pcbi.1006483.s042.png]

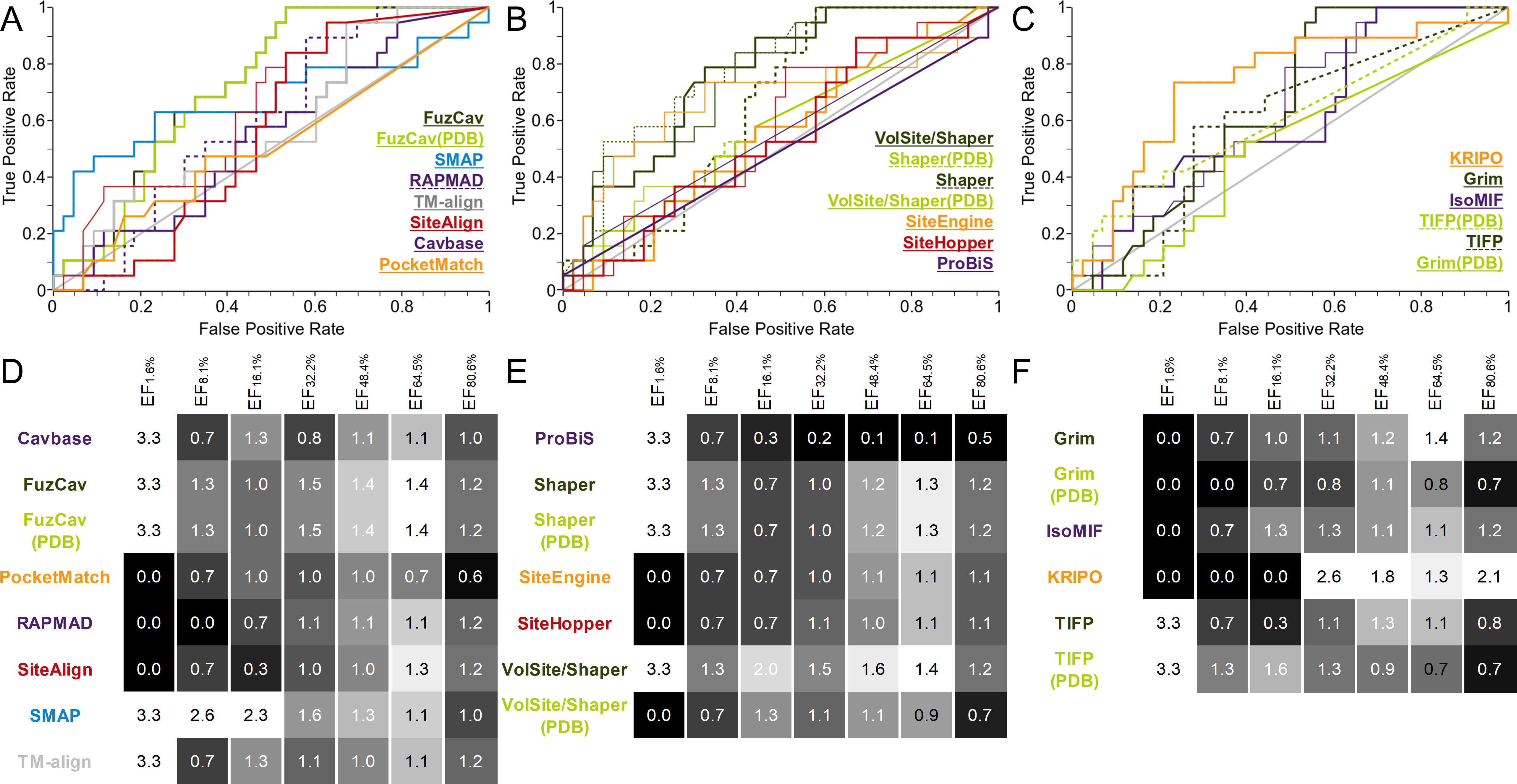

Supplement: S9 Fig — A-C) The ROC curves for residue- (A), surface- (B), and interaction-based (C) comparison methods. The name of the tool is colored according to its corresponding ROC curve. The binding site comparison tools are sorted in descending order with respect to the AUC. (A) The thin red line represents the resulting ROC curve for SiteAlign when using the distance d1. (B) Thin lines represent the ROC curves for ProBiS, Shaper, Shaper(PDB), VolSite/Shaper, VolSite/Shaper(PDB), SiteEngine and SiteHopper when using the scoring schemes SVA, FitTversky (color), FitTversky (color), RefTversky (color), Tanimoto (fit), DistanceScore, and ShapeTanimoto, respectively. (C) The thin line represents the resulting ROC curve for IsoMIF and the taniMW score. D-F) EFs for residue- (D), surface- (E), and interaction-based (F) comparison methods. A linear color gradient ranging from white for the highest value to gray to black for the lowest value was applied for the EFs at different percentages of screened data set. (PNG) [file pcbi.1006483.s043.png]

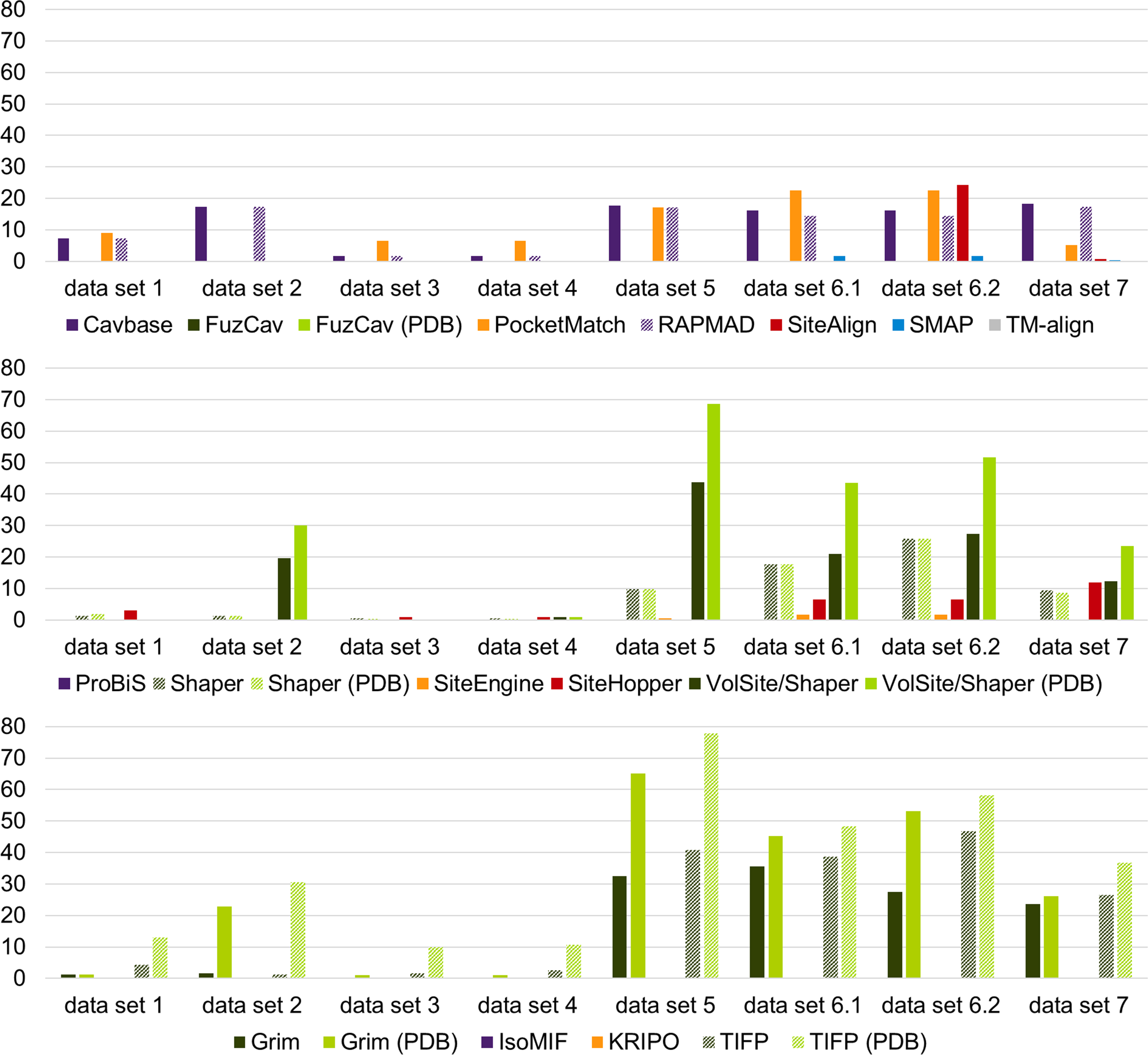

Supplement: S10 Fig — (PNG) [file pcbi.1006483.s044.png]
